# Electronic structure engineering of Zn-based catalysts *via* anionic regulation for polysulfide adsorption–catalysis in Li–S batteries

**DOI:** 10.1039/d6sc03397k

**Published:** 2026-06-04

**Authors:** Cheng He, Lingfeng Zhu, Jia Fu, Xiangzeng Meng, Hongfu Liu, Youliang Wang, Zhenfang Zhang, Xiaoning Li, Hui Li, Tianyi Ma, Sheng Liu, Yuhai Dou, Ji Yu, Jianxin Cai, Ze Zhang

**Affiliations:** a School of Chemistry and Chemical Engineering, Key Laboratory Foundation of Jiangxi Province for Environment and Energy Catalysis, Nanchang University Nanchang 330031 China zhangze@ncu.edu.cn; b Centre for Atomaterials and Nanomanufacturing (CAN), School of Science, RMIT University Melbourne VIC 3000 Australia lingfeng.zhu@rmit.edu.au tianyi.ma@rmit.edu.au; c ARC Industrial Transformation Research Hub for Intelligent Energy Efficiency in Future Protected Cropping (E2Crop) Melbourne VIC 3000 Australia; d School of Materials Science and Engineering, Nankai University Tianjin 300350 China; e Centre for Catalysis and Clean Energy, Gold Coast Campus, Griffith University Gold Coast 4222 Australia

## Abstract

Sluggish sulfur redox kinetics and polysulfide shuttling significantly compromise the cycling stability and sulfur utilization of lithium–sulfur batteries (LSBs). Efficient catalytic conversion of polysulfides is a pivotal strategy to address these issues, yet remains challenged by ambiguous electronic structure–performance relationships and limited catalytic efficacy. We rationally designed zinc-based compounds encapsulated in N-doped porous carbon (ZnX@PC, X = O/S/Se) to modulate the orbital electronic structure of zinc centers, serving as catalysts for accelerating sulfur redox kinetics and suppressing polysulfide migration in LSBs. The battery performance with these zinc-based catalysts was systematically evaluated, with a particular focus on how p-band characteristics of anions (O/S/Se) govern d–p orbital hybridization between Zn center and S in polysulfides. Compared to O and S, the Se anion optimizes the Zn d-band center closer to the Fermi level in ZnSe compounds. The ZnSe@PC catalyst exhibits balanced polysulfide adsorption and the most favorable d-band proximity to the Fermi level, demonstrating optimal adsorption–catalysis synergy for polysulfides. Electrochemical testing revealed an initial specific capacity of 1526 mAh g^−1^ at 0.1C for ZnSe@PC-based cells, confirming high sulfur utilization efficiency. Under challenging conditions (E/S ratio = 4.0 µL mg^−1^, sulfur loading = 7.0 mg cm^−2^), the pouch cell system delivered an exceptional capacity of 953.3 mAh g^−1^ at 0.1C, representing one of the best performances reported for high loading sulfur cathodes. Consequently, this work deepens the mechanistic understanding of LSBs and offers empirical validation for the conventional theory regarding the influence of the d-band center on battery performance.

## Abstract 2

This work designs zinc-based compounds encapsulated in carbon to modulate the electronic structure of Zn centers, serving as catalysts for Li–S batteries. ZnSe@PC achieves balanced adsorption and efficient catalytic conversion of polysulfides.

## Graphical abstract

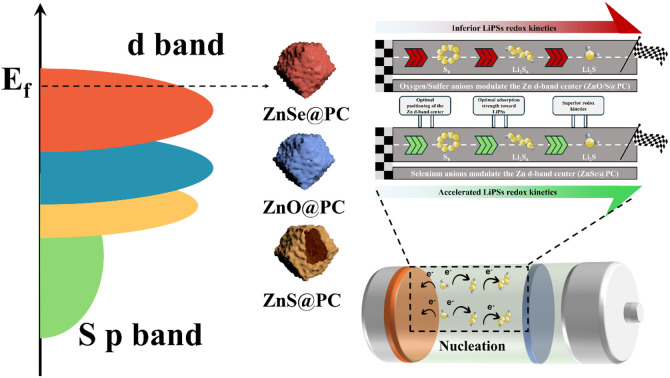

## 1 Introduction

Although lithium-ion batteries (LIBs) currently dominate the markets for portable electronics, electric vehicles and stationary storage, their limited theoretical specific energy (≈350 Wh kg^−1^) falls short of meeting the growing energy demands.^1–7^ Consequently, developing novel energy storage systems to replace LIBs is imperative. Among various alternatives, lithium–sulfur (Li–S) batteries have emerged as a focus of extensive research for next-generation high-energy systems due to the key advantages including high theoretical energy density (2600 Wh kg^−1^), abundant sulfur resources and low cost.^8–11^ However, the practical implementation of Li–S batteries faces significant challenges related to the inherent dissolution–deposition mechanism of sulfur cathodes, including the insulating nature of sulfur, the shuttle effect caused by the dissolved lithium polysulfides (LiPSs), and the sluggish reaction kinetics.^12–15^ To address these challenges, researchers have developed various host materials to suppress the shuttle effect through physical confinement or chemical adsorption of LiPSs.^16–18^ However, both physical confinement and chemical adsorption passively retain LiPSs at the cathode side without addressing the root cause of the shuttle effect.^19^ Thus, under prolonged cycling or high sulfur-loading conditions, these materials lose effectiveness due to adsorption saturation and suffer the degradation of energy density and cycle life.^20^

Recent research has integrated catalytic strategies into Li–S batteries, and the adopted catalysts anchor LiPSs through strong adsorption, effectively suppressing the shuttle effect.^21^ Concurrently, they can effectively reduce the energy barriers for LiPSs reduction and Li_2_S oxidation, thereby accelerating reaction kinetics to enhance capacity retention and cycle life.^22^ Extensive research has focused on transition metal compounds (TMCs)—including oxides, sulfides, nitrides, and phosphides—as catalysts to enhance sulfur redox kinetics.^23–32^ They exhibit strong chemisorption toward LiPSs through metal–sulfur (M–S) bonding interactions, as widely documented in the literature.^33^ This interaction weakens Li–S and S–S bonds in LiPSs, facilitating the conversion of long-chain LiPSs to short-chain LiPSs and finally Li_2_S. However, excessively strong chemisorption induces premature LiPSs decomposition and catalyst passivation, while weak adsorption fails to suppress polysulfide shuttling.^34–37^ Selecting materials with optimal adsorption strength is therefore critical, as dictated by the Sabatier principle.^38^ Besides, focusing solely on the chemisorption of LiPSs by TMCs fails to account for the performance variations among catalysts. Resolving these challenges necessitates probing the intrinsic catalytic mechanisms of TMCs to identify key descriptors of the catalytic activity. Although systematic investigations remain limited, pioneering studies have demonstrated the feasibility of electronic structure modulation in catalytic systems.^39^ For instance, Lu *et al.* combined experimental and theoretical approaches to engineer the d-band center of Co_9_S_8_@MoS_2_ heterostructures, revealing its critical role in regulating polysulfide adsorption behavior. This electronic restructuring significantly enhanced the conversion kinetics of LiPSs, accelerating redox reactions and improving sulfur utilization in Li–S batteries.^36^ Zhou *et al.* systematically investigated the influence of nonmetal anions (*e.g.*, P, S, N) in cobalt-based compounds on sulfur reaction kinetics, revealing that anion selection fundamentally modulates electronic structures through p–d orbital hybridization. Their analysis demonstrated that phosphorus induces upward shifting of the cobalt d-band center while positioning its p-band closer to the Fermi level. This synergistic electronic configuration in cobalt phosphide (CoP) enhances charge transfer efficiency, reducing the Li_2_S decomposition energy barrier to 0.35 eV and improving sulfur utilization by 7% compared to cobalt sulfide counterparts.^40^ Yu *et al.* revealed that iron phosphide (FeP) exhibits superior catalytic activity to magnetite (Fe_3_O_4_) due to optimized d-band positioning and enhanced charge transfer efficiency.^41^

In recent years, zinc-based compounds have demonstrated unique advantages as cathode materials for LSBs.^42^ The fully occupied 3d^10^ electronic configuration of Zn^2+^ facilitates hybridization with the p-orbitals of sulfur, inducing the formation of abundant unsaturated coordination sites. Compared to conventional transition metals, the fully occupied d-orbitals of Zn result in a lower-lying d-band center. This leads to a moderately attenuated adsorption strength for LiPSs, effectively avoiding active site poisoning caused by overly strong adsorption and thereby ensuring stability during catalytic cycling.^43^ On the other hand, although the fully occupied d-orbitals of Zn do not participate in strong covalent bonding, they can donate electrons to the σ* antibonding orbitals of LiPSs during discharge. This significantly weakens the S–S bonds and promotes their cleavage.^44^ This mechanism facilitates the desorption of Li_2_S from the zinc-based catalyst surface and lowers the energy barrier for LiPSs conversion. Consequently, the distinctive electronic structure of zinc-based materials achieves an optimal balance between “moderate anchoring” and “efficient catalysis” of polysulfides, leading to a significant enhancement in the overall reaction kinetics.^45^ The electronic structure and catalytic behavior exhibited by zinc-based compounds can be essentially explained by the universal d–p orbital hybridization mechanism between transition metals and sulfur species.^46–48^ This electronic coupling governs both the adsorption strength of sulfur intermediates and the energy barriers for their conversion reactions. Transition metal centers with partially filled d-orbitals serve as catalytic active sites, where d-band center positioning modulates electron transfer efficiency during redox processes.^49–51^ The adsorption strength between catalytic surfaces and reaction intermediates can be quantitatively correlated with two key electronic parameters: the energy position of the d-band center relative to the Fermi level (*E*_F_), and the occupancy of antibonding orbitals formed during surface adsorption. An upward shift in the d-band center position enhances the adsorption strength between metals and LiPSs by optimizing electron transfer at the interface.^52^

Guided by this principle, we engineered zinc-based compounds wrapped in nitrogen-doped porous carbon (ZnX@PC) with O, S, and Se anions to modulate the 3d orbital electronic structure of zinc centers. The Li–S battery performance with these zinc compounds as catalytic hosts was evaluated to investigate the effect of p-band characteristics of anions (O/S/Se) on the d–p orbital hybridization between Zn in ZnX and S in LiPSs. Density functional theory (DFT) results demonstrate that, compared to O and S, Se anion causes an upward shift in the d-band center of Zn toward the Fermi level in ZnSe compounds, leading to the best catalytic acitivity of ZnSe in Li–S batteries. Systematic X-ray absorption spectroscopy analyses confirm that anion modification (with O, S or Se) effectively regulates the electronic structure and local coordination environment of Zn-based host materials (ZnX@PC). Meanwhile, the rational designed hierarchical porous carbon matrix with highly active ZnX catalysts creates abundant anchoring sites for LiPSs, and simultaneously accelerates Li-ion diffusion kinetics. Thus, the battery with ZnSe@PC catalyst shows the reduced LiPSs decomposition/conversion energy barrier, and rapid Li_2_S deposition with nucleation rate up. Electrochemical testing revealed that the battery with ZnSe@PC host achieves a high initial specific capacity of 1526 mAh g^−1^ at 0.1C, demonstrating an efficient sulfur utilization. Notably, under challenging operational parameters of an electrolyte/sulfur ratio of 4.0 µL mg^−1^ and a high sulfur loading of 7.0 mg cm^−2^, the S/ZnSe@PC pouch cell delivers an exceptional capacity of 953.3 mAh g^−1^ at 0.1C.

## 2 Results and discussion

**Fig. 1:**
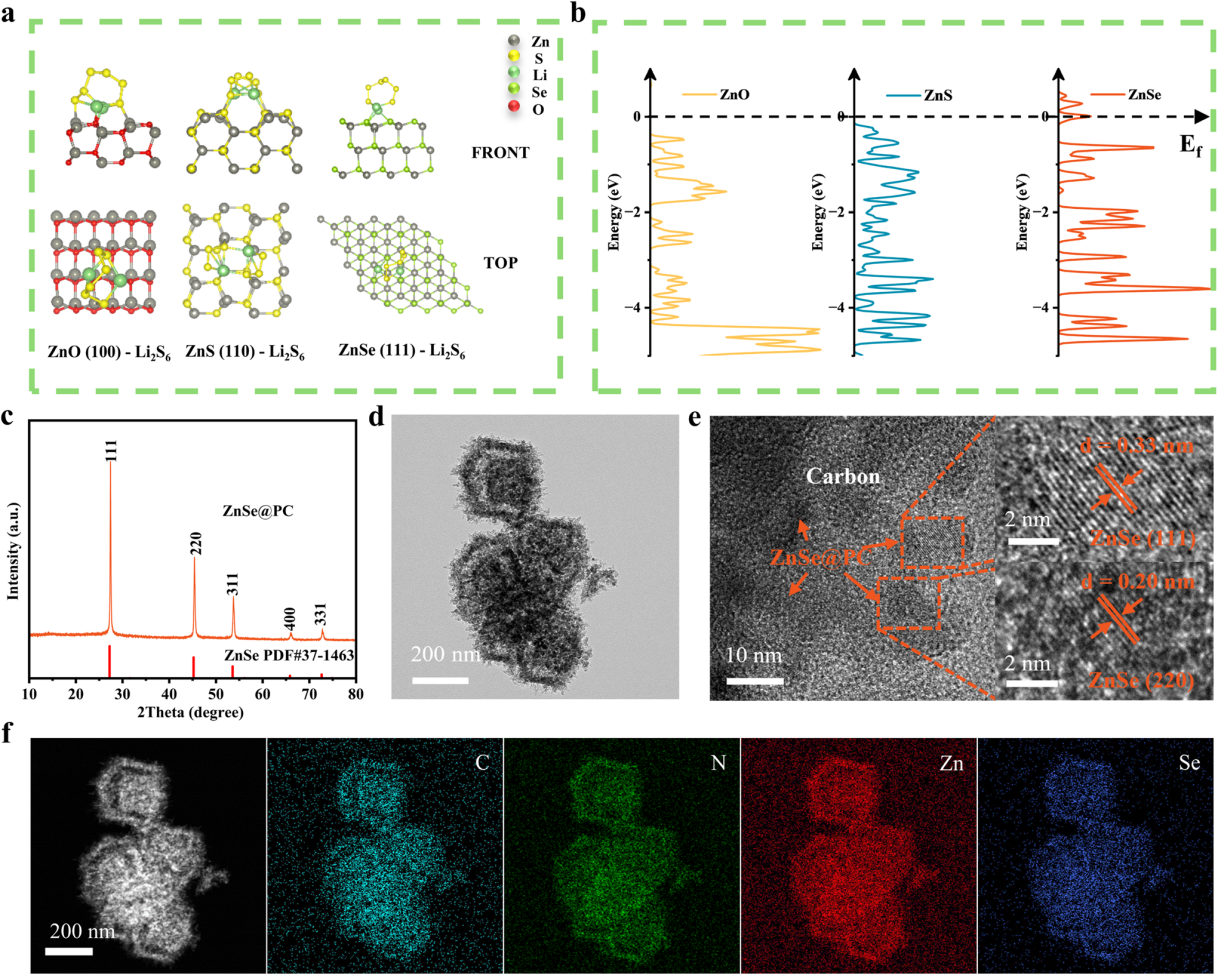
(a) The adsorption energy and differential charge density of the Li_2_S_6_ on ZnO, ZnS and ZnSe systems. (b) Electronic structures of metal sites in different ZnX catalysts. (c) XRD pattern, (d) TEM image, (e) HRTEM image, and (f) STEM image and EDX images of the as-prepared ZnSe@PC sample.

We first established and optimized the structural models of three ZnX (X = O/S/Se) (Fig. S1, SI). Subsequently, an in-depth investigation into the relationship between the d-band center and catalytic activity was conducted by using DFT calculations. The adsorption capability of Li_2_S_6_ on the ZnX (X = O/S/Se) surface was initially investigated. DFT results (Fig. 1a) show that the adsorption energy of Li_2_S_6_ are calculated as −2.31 eV, −1.24 eV, and −1.86 eV on ZnO (100), ZnS (110), and ZnSe (111) surface, respectively. The moderate Li_2_S_6_ adsorption energy of ZnSe forebodes its best redox catalytic ability in Li–S batteries. Meanwhile, the Zn d-band position in ZnX are further investigated in Fig. 1b, and Se anion-induces Zn d-band center in ZnSe upshifting toward the Fermi level, compared with O and S. Thus, the Zn d-band properties in ZnX compounds are effectively regulated by the p orbitals of O/S/Se, further indicating the optimal LiPSs adsorption–catalysis in Li–S batteries.

As for the material synthesis, ZIF-8 was adopted as the precursors to yield ZnX compounds encapsulated in N-doped porous carbon (ZnX@PC), as illustrated in Fig. S2 (SI). X-ray diffraction (XRD) patterns of ZIF-8 (Fig. S3, SI) exhibits characteristic peaks consistent with those reported for ZIF-8 in the literatures, confirming its successful formation.^53^ Scanning electron microscopy (SEM) and transmission electron microscopy (TEM) images (Fig. S4, SI) further revealed the characteristic uniform polyhedral morphology of the synthesized ZIF-8 precursor, with particle sizes of approximately 500 nm. Fig. 1c displays the XRD pattern of ZnSe@PC composite. Distinct diffraction peaks of the crystal planes of ZnSe are observed, confirming the successful synthesis of a pure-phase ZnSe@PC composite matching the reference pattern (PDF#37-1463). Similarly, distinct crystal planes were observed in both ZnO@PC and ZnS@PC composites (Fig. S5, SI).

X-ray photoelectron spectroscopy (XPS) analysis was subsequently performed to elucidate the elemental composition and chemical bonding states. Full XPS spectra in Fig. S6 suggests the existence of characteristic elements in ZnX@PC materials. The Se 3d spectrum of ZnSe@PC (Fig. S7c, SI) exhibits peaks at binding energies of 53.79 eV and 54.71 eV, assigned to Se 3d_5/2_ and Se 3d_3/2_ respectively, corresponding to Zn–Se interactions in ZnSe. An additional peak at 59.17 eV is attributed to Se–O species, likely originating from surface-oxidized SeO_*x*_.^54^ Fig. S7a and b presents the O 1s region for the ZnO@PC sample and the S 2p region for the ZnS@PC sample. High-resolution N 1s spectra of the ZnX@PC series (Fig. S8, SI) reveal three characteristic peaks at approximately 398 eV, 400 eV, and 401 eV, assigned to pyridinic-N, pyrrolic-N, and graphitic-N configurations, respectively.^55^ This confirms effective nitrogen doping feature of porous carbon substrates in ZnX@PC series. The lone-pair electrons of N atoms can form electrostatic interactions with the vacant orbitals of terminal Li^+^ ions in polysulfides, enabling effective chemisorption of polysulfide species. Furthermore, optimal nitrogen doping enhances the electronic conductivity of the material. Recent studies reveal that nitrogen doping, particularly pyridinic-N configuration, confers intrinsic electrocatalytic activity to carbon matrices, accelerating sulfur redox kinetics and reducing the energy barrier for Li_2_S decomposition.^56,57^

Raman spectroscopy was employed to evaluate the graphitization degree and defect characteristics of the samples. The broad peak at 1426 cm^−1^ is attributed to N–N stretching modes in nitrogen-doped carbon.^58^ Besides, distinct peaks observed at 1335 cm^−1^ (D-band) and 1575 cm^−1^ (G-band) in Fig. S9 (SI) correspond to disordered carbon and graphitic structures, respectively.^59,60^ Disordered carbon domains provide abundant active sites, while graphitic regions enhance electrical conductivity, and an optimized *I*_D_/*I*_G_ ratio thus contributes to enhanced electrocatalytic performance. ZnSe@PC demonstrates the most favorable *I*_D_/*I*_G_ ratio (0.85), indicating the availability of superior catalytic site.^61^ TEM image (Fig. 1d) and SEM image (Fig. S10, SI) reveal ZnSe nanoparticles uniformly distributed on the inner walls of nitrogen-doped porous carbon structures. High-resolution TEM images (Fig. 1e) display distinct lattice fringes with interplanar spacings of 0.33 nm and 0.20 nm, corresponding to the (111) and (220) planes of ZnSe phase, respectively. The corresponding energy dispersive X-ray spectroscopy (EDX) elemental mapping (Fig. 1f) demonstrates the uniform elemental distribution of Zn, Se, C and N throughout the ZnSe@PC composite. These results confirm the successful synthesis of ZnSe@PC and the well inherited morphology and porous structure through the pyrolysis and selenization of ZIF-8. TEM observations in Fig. S11 and S12 (SI) demonstrate the analogous morphology and distinct lattice fringes for ZnO@PC and ZnS@PC materials with uniform elemental distribution.

Carbon content of ZnX@PC samples were measured by thermogravimetric analysis (TGA) with detailed quantitative data provided in Fig. S13. The carbon content in the ZnSe@PC composite was determined to be 62.8 wt%. The porous structures of ZnX@PC samples were also characterized through N_2_ adsorption–desorption isotherms (Fig. S14, SI). ZnSe@PC possesses the highest specific surface area, significantly exceeding those of ZnO@PC and ZnS@PC. This enhanced surface area facilitates continuous electrolyte infiltration, ensuring thorough wetting of catalytic active sites. After the accommodation of sulfur on ZnX@PC samples, the sepcific surface areas greatly reduced (Fig. S15, SI), demonstrating the effective encapsulation of active sulfur within the porous framework. Comprehensive parameters are provided in Table S1 (SI). XRD patterns (Fig. S16, SI) of S/ZnX@PC composites also reveal the distinct diffraction signatures of crystalline sulfur. Typically, TEM images (Fig. S17, SI) revealed that the S/ZnSe@PC sample maintains its original morphology after loading with sulfur, and EDX elemental maps confirm the uniform distribution of sulfur on the composite. TGA results (Fig. S18, SI) quantify the sulfur contents in S/ZnX@PC at approximately 75%, closely matching the initial feeding ratio.

**Fig. 2:**
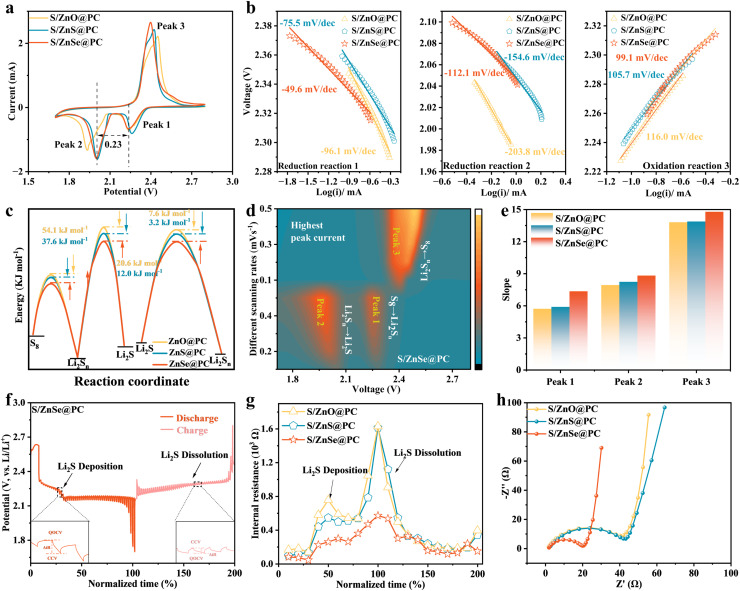
Redox reaction kinetics characterization. (a) CV profiles. (b) Tafel slope from the CV curve. (c) Difference in activation energies (Δ*E*_a_) during different conversion processes on these electrodes. (d) Contour plots of CV patterns for S/ZnSe@PC. (e) The linear relationship between *I*_p_ and *ν*^0.5^. (f) GITT curve of S/ZnSe@PC. (g) Variation of internal resistance during charging and discharging of S/ZnX@PC during normalization time. (h) EIS curves for S/ZnX@PC.

Electrochemical performance of Li–S batteries of S/ZnX@PC composites are firstly evaluated by cyclic voltammetry (CV) tests. In Fig. 2a, all CV curves exhibit two distinct cathodic peaks at about 2.24 V (peak 1) and 2.05 V (peak 2), corresponding to the two-step conversion of S_8_ to soluble long-chain LiPSs, and insoluble Li_2_S_2_/Li_2_S, respectively. Meanwhile, the anodic peak at about 2.36 V (peak 3) reflects the inverse transformation of insoluble Li_2_S_2_/Li_2_S into long-chain LiPSs and eventual reconversion to S_8_. Notably, S/ZnSe@PC demonstrates highest redox peak currents and smallest polarization potential (Δ*E* = 0.23 V), indicating superior catalytic efficiency for polysulfide conversion reactions.^62^ Tafel slopes derived from CV curves serve as reliable indicators of charge transfer kinetics, where lower values signify accelerated LiPSs redox kinetics. As shown in Fig. 2b, S/ZnSe@PC displays significantly lower Tafel slopes for the peaks compared to other electrodes. This confirms ZnSe@PC's superior catalytic efficiency in reducing redox energy barriers and enhancing charge transfer during sulfur species conversion. Combining Tafel analysis with relevant kinetic equations reveals activation energy variations in LiPSs conversion pathways, as illustrated in Fig. 2c.^63,64^ Calculations reveal that in the reduction peaks 1, the activation energy differences for S_8_-to-LiPSs conversion are 54.1 kJ mol^−1^ and 37.6 kJ mol^−1^ compared to S/ZnO@PC and S/ZnS@PC electrodes, respectively. Correspondingly, the LiPSs-to-Li_2_S transformation exhibits differences of 20.6 kJ mol^−1^ and 12.0 kJ mol^−1^. The activation energy differences for Li_2_S to Li_2_S_4_ conversion at the oxidation peak 3 are 7.6 kJ mol^−1^ and 3.2 kJ mol^−1^, respectively. These results confirm that the ZnSe@PC host enhances LiPSs conversion kinetics by reducing activation energy barriers in redox processes.

Fig. 2d displays CV contour plots of S/ZnSe@PC, and Fig. S19 (SI) displays the CV contour plots of S/ZnX@PC (X = O, S). Notably, the peak 3 region in S/ZnSe@PC exhibits enhanced contour intensity and higher current density. The increased peak current and reduced polarization voltage signify improved conversion kinetics from S_8_ to soluble LiPSs and ultimately to insoluble Li_2_S, confirming ZnSe@PC's superior catalytic capability in facilitating LiPSs-to-S_8_ reconversion. Furthermore, Li^+^ diffusion kinetics constitute another critical parameter governing LiPSs redox kinetics. CV measurements reveal a strong linear correlation between peak current (*I*_p_) and the square root of scan rate (*v*^0.5^) in Fig. S20 (computational details are provided in Table S2, SI),^65^ and the S/ZnSe@PC cathode exhibits a substantially steeper slope than the other two cathodes (Fig. 2e). This enhanced slope signifies superior Li^+^ diffusion kinetics and accelerated conversion rates according to Randles–Sevcik equation, thereby facilitating more efficient lithium polysulfide transformation.^66,67^

Galvanostatic intermittent titration technique (GITT) was additionally performed. As shown in Fig. 2f and S21 (SI), all three cathodes exhibit characteristic charge/discharge voltage plateaus, and the S/ZnSe@PC cathode demonstrates significantly reduced polarization plateaus during both processes compared to counterparts. Furthermore, Fig. 2g shows that the S/ZnSe@PC cathode exhibits significantly reduced internal resistance during both Li_2_S nucleation and Li_2_S oxidation. This demonstrates that its porous architecture facilitates Li-ion diffusion pathways while effectively suppressing the shuttle effect. Electrochemical impedance spectroscopy (EIS) of batteries assembled with S/ZnX@PC reveals Nyquist plots comprising semicircles and sloped lines (Fig. 2h), corresponding to interface resistance and Warburg impedance respectively. The high-frequency intercept of the semicircle represents the intrinsic internal resistance. S/ZnSe@PC cathode exhibits the lowest values for the resistances. Fitting analysis of the Warburg impedance (Fig. S22, SI) confirms a reduced *σ* value for S/ZnSe@PC, indicating enhanced Li-ion migration kinetics.

**Fig. 3:**
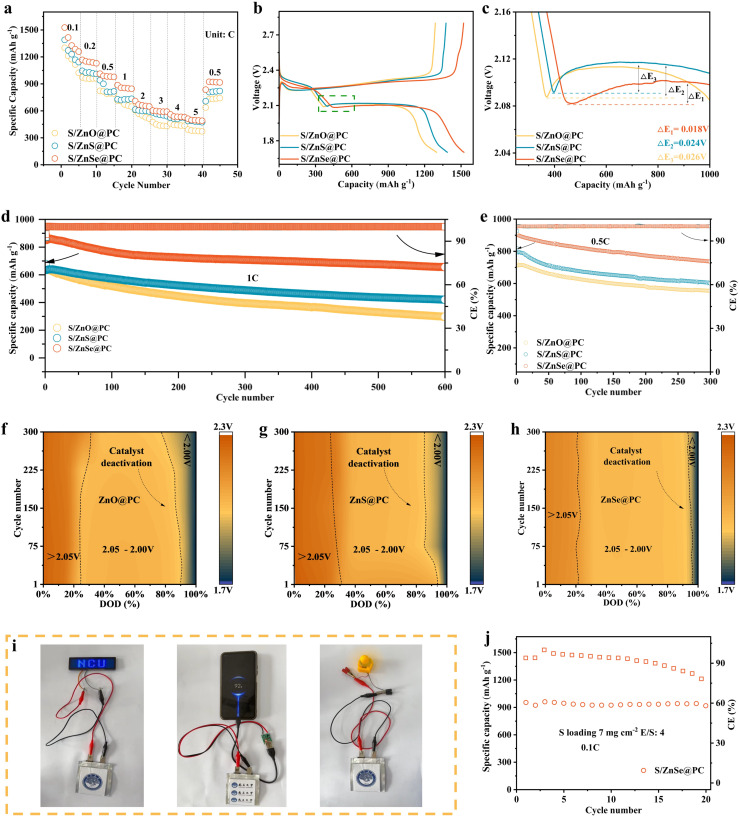
(a) Rate performance comparison of S/ZnX@PC cathodes. (b) Charge/discharge curves at 0.1C, and (c) magnified view of the charge/discharge curves. Cycling performance at (d) 1C, and (e) 0.5C. (f–h) Contour plots of discharge profiles during 300 cycles using cathodes based on different catalysts at 0.5C. (i) Demonstrating the utility of pouch cells. (j) Cycling performance of the S/ZnSe@PC pouch cell at 0.1C.

Fig. 3a shows the electrochemical rate capability of S/ZnX@PC cathodes and S/ZnSe@PC cathode demonstrates superior rate capability, delivering discharge capacities of 1526,1166, 1012, 884, 708 and 559 mAh g^−1^ at 0.1C, 0.2C, 0.5C, 1C, 2C and 5C respectively. When the current density returned to 0.5C, it maintains notable electrochemical reversibility with a reversible capacity of 834 mAh g^−1^. In contrast, other cathodes exhibit lower capacities under identical current rates. In the galvanostatic charge/discharge (GCD) profiles (Fig. 3b), the discharge curves exhibit two voltage plateaus at 2.3 V and 2.1 V, corresponding to the conversion of S_8_ to LiPSs and their reduction to Li_2_S_2_/Li_2_S, respectively. Fig. 3c shows the distinct trough at the transition zone between discharge plateaus indicates the Li_2_S nucleation point. The potential difference between the trough minimum and the tangent to the second plateau quantifies the nucleation barrier, serving as a kinetic indicator. S/ZnSe@PC cells demonstrate the lowest nucleation overpotential, substantially outperforming other systems. This confirms S/ZnSe@PC enhances Li_2_S nucleation and deposition processes. Further analysis on the capacity contribution of two discharge plateau, defined as *Q*_1_ and *Q*_2_, provides a key catalyst evaluation parameter, and a *Q*_1_/*Q*_2_ ratio approaching 25% indicates optimal catalytic efficiency for liquid-to-solid transition, enabling higher capacity output during solid-phase deposition and accelerating sulfur reduction kinetics. S/ZnSe@PC cathodes exhibit *Q*_1_/*Q*_2_ ratios closest to the theoretical optimum (Fig. S23, SI), validating the superior electrocatalytic activity of ZnSe compared to ZnO and ZnS. In-depth analysis of the rate capability, as evidenced by the charge/discharge profiles (Fig. S24, SI), reveals that the S/ZnSe@PC cathod exhibits significantly lower polarization voltages across all C-rates compared to S/ZnO@PC and S/ZnS@PC.

As for the long-term cycling stability in Fig. 3d, the S/ZnSe@PC cathode provides an initial capacity of 860.5 mAh g^−1^ at 1C, and maintains a high reversible capacity of 497.4 mAh g^−1^ after 600 cycles, suggesting a low capacity decay rate of 0.043% per cycle, whereas S/ZnO@PC and S/ZnS@PC exhibit 0.091% and 0.066%, respectively. Fig. 3e demonstrates that at 0.5C, the S/ZnSe@PC cathode achieves an initial capacity of 904.3 mAh g^−1^ and retains 735.6 mAh g^−1^ after 300 cycles, corresponding to a capacity retention of 81.3%. Post-cycling SEM characterization of the cathodes revealed that different modifying materials exert markedly different effects on electrode structural stability. As shown in Fig. S25a and b (SI) the S/ZnO@PC and S/ZnS@PC electrodes display rough, porous surfaces with evident cracks, which can be attributed to structural damage caused by volume changes of the active material during cycling. Such structural deterioration not only aggravates the polysulfide shuttle effect but also leads to the loss of active sulfur species. In contrast, the S/ZnSe@PC electrode (Fig. S25c, SI) exhibits a smoother, denser morphology and maintains good structural integrity. These results demonstrate that ZnSe@PC effectively suppresses the shuttle effect and buffers volume expansion, thereby imparting excellent structural stability to the electrode, which is in strong agreement with its superior electrochemical performance. Furthermore, SEM characterization of the cycled separator and lithium metal anode, presented in Fig. S26 (SI), confirms that the ZnSe@PC host effectively suppresses the polysulfide shuttle effect. As shown in Fig. 3f–h, the three batteries were subjected to a 300-cycle test at 0.5C to investigate the evolution of polarization. Throughout the initial 200 cycles, all batteries employing zinc-based compounds exhibited low polarization, with the voltage gap maintained below 2.05 V. We attribute this performance to the unique fully occupied d^10^ electronic configuration of zinc-based compounds. This configuration results in a relatively low d-band center position for Zn, which mitigates excessive adsorption of LiPSs. This moderate, weakened adsorption effectively prevents the poisoning of active sites associated with overly strong adsorption, ensuring the long-term continuity of the catalytic cycles. Consequently, it endows the batteries with excellent long-term cycling stability. However, prolonged cycling inevitably triggers the deactivation of catalytic active sites due to “dead sulfur” accumulation, accelerating performance degradation. By contrast, S/ZnSe@PC maintains minimal polarization throughout 300 cycles owing to the enhanced sulfur conversion efficiency and the suppressed catalyst passivation.

Increasing sulfur loading and minimizing electrolyte usage are essential to achieve high energy density Li–S batteries. As shown in Fig. S27 (SI), with an E/S ratio of 6 µL mg^−1^ and sulfur loading of ∼4 mg cm^−2^, the S/ZnSe@PC cathode demonstrates higher capacity and superior cycling stability compared with S/ZnO@PC and S/ZnS@PC cathodes at 0.2C. The corresponding GCD curves of three cathodes in Fig. S28 (SI) also showing that S/ZnSe@PC cathode has good stability and a small polarization voltage. Further test with a higher sulfur loading of 5.2 mg cm^−2^ is conducted on the S/ZnSe@PC cathode (Fig. S29, SI). The cathode delivers an initial discharge capacity of 895.2 mAh g^−1^, and maintains stable cycling over 160 cycles with an E/S ratio of 5 µL mg^−1^ at 0.1C. Besides, the cathodes show average coulombic efficiencies above 95% during the long-term cycle. Moreover, pouch cells are fabricated with a 7 mg cm^−2^ loading S/ZnSe@PC electrode (50 mm × 80 mm in size) using double-sided coating, and E/S ratio is controlled as 4 µL mg^−1^. Fig. 3i demonstrates its ulitity in the powering illuminated signage, sustaining portable device charging, and activating micro-fan arrays (Videos S1–S3). Post-activation cycling at 0.1C demonstrates a high specific capacity of 953.3 mAh g^−1^, and a capacity retention of 96.2% after 20 cycles (Fig. 3j). Galvanostatic profiles (Fig. S30, SI) show the maintained representative discharge/charge plateau under the harsh operation conditions, suggesting the great potential in pratical Li–S battery application. Overall, the above results vividly demonstrates the great superiority of ZnSe@PC catalyst in fabricating high-performance Li–S batteries with excellent electrochemical performance for both coin cells and pouch cells compared with the repored promising catalysts (details can be seen from Tables S3–S4, SI). To clearly distinguish the respective roles of the carbon carrier and the active sites, we evaluated the electrochemical performance of a control sample, N-doped porous carbon (PC), which lacks ZnX modification (Fig. S31, SI). The results indicate that the S/PC electrode, devoid of O/S/Se-induced electronic structure modulation, exhibited significantly inferior rate performance. It delivered a capacity of only 980.1 mAh g^−1^ at 0.1C, which plummeted to 250 mAh g^−1^ at a high current density of 5C, showing a considerable gap compared to the S/ZnO@PC, S/ZnS@PC, and S/ZnSe@PC electrodes (Fig. S31a). Further long-term cycling tests at 1C in Fig. S31b (SI) revealed that S/PC had an initial capacity of merely 323.5 mAh g^−1^, which rapidly decayed to below 100 mAh g^−1^ by the 200th cycle. This outcome confirms that although the N-doped porous carbon substrate provides physical confinement and electron conduction, the bare Zn^2+^ sites lack effective electronic structure modulation. Consequently, they cannot supply sufficient catalytic activity to drive the sluggish polysulfide conversion reaction.

To elucidate the mechanistic enhancement of material design on Li–S batteries, the polysulfide adsorption–catalysis abilities of ZnSe@PC catalysts were systematically studied. Adsorption tests for LiPSs were conducted by immering equal masses of ZnX@PC powders in Li_2_S_6_ solution for 6 hours (Fig. S32, SI). The solution containing ZnSe@PC exhibits the visibly lightest coloration among the samples, and UV-vis analysis of the supernatant reveals the lowest absorbance intensity for ZnSe@PC, indicating its superior LiPSs adsorption capacity. Subsequently, XPS was employed on the dried powder samples to investigate interfacial interactions between LiPSs and ZnX@PC. As shown in Fig. S33 (SI), the Zn 2p peaks of all three samples shift toward lower binding energy (BE), indicating the increased electron density around Zn atoms after interacting with LiPSs. Notably, ZnSe@PC exhibits the most pronounced BE shift after LiPSs adsorption compared to counterparts, demonstrating stronger interfacial interactions between ZnSe@PC and LiPSs species. Moreover, Raman spectra in Fig. S34 (SI) displays distinct Zn–S bond signals for ZnSe@PC after LiPSs adsorption, confirming its superior affinity toward sulfur species. Such enhanced adsorption capability critically determines the electrochemical performance of Li–S batteries. To further investigate the suppression of polysulfide shuttling by ZnX@PC, shuttle current tests were conducted using electrolyte without LiNO_3_ additive, which effectively validates the material's capability to immobilize soluble intermediates. As shown in Fig. S35 (SI), ZnSe@PC exhibits significantly lower shuttle currents (<0.01 mA cm^−2^), attributed to its superior binding affinity for LiPSs species. This confirms ZnSe@PC's efficacy in suppressing shuttling during redox reactions.

**Fig. 4:**
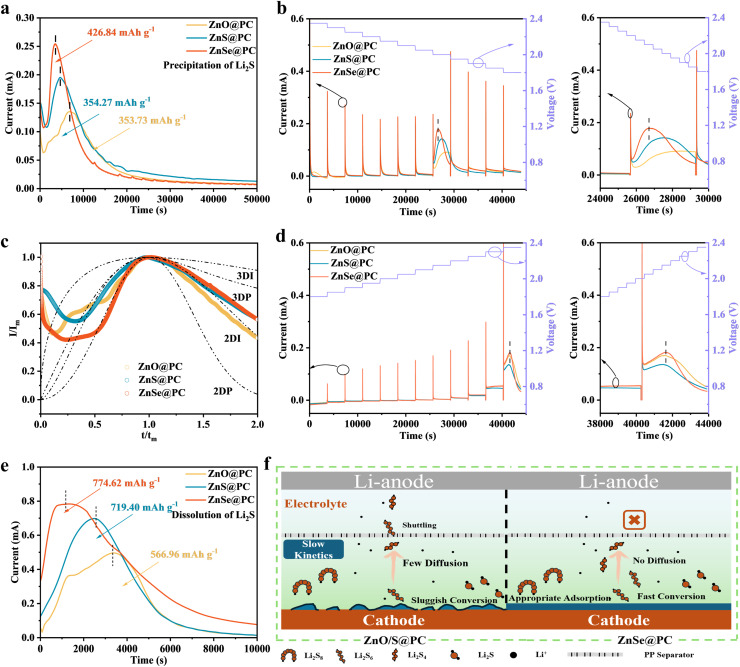
(a) The nucleation of Li_2_S on different electrodes. (b) PITT curves at 2.35–1.8 V of ZnX@PC and the local magnification. (c) Dimensionless current–time transients for Li_2_S deposition on ZnX@PC. (d) PITT curves at 1.8–2.35 V of ZnX@PC and the local magnification. (e) The dissolution of Li_2_S on different electrodes. (f) Schematic representation of the adsorption–diffusion–conversion process of LiPSs on ZnX@PC.

Liquid–liquid phase transtion kinetics was investigated by using symmetric cells assembled using ZnX@PC-loaded graphite felt electrodes at the presence of Li_2_S_6_ solution. CV curves recorded between −0.8 and 0.8 V (Fig. S36a, SI) exhibit symmetrical spindle shapes for all configurations. The ZnSe@PC-based cell demonstrates the highest redox current density and largest integrated CV area. This indicates superior catalytic activity of ZnSe@PC in accelerating liquid–liquid conversion kinetics. Furthermore, Tafel analysis (Fig. S36b, SI) reveals an exchange current density of 109.65 µA cm^−2^ for ZnSe@PC, confirming significantly enhanced electrochemical conversion kinetics. Furthermore, the Nyquist plots obtained from EIS of Li_2_S_6_ symmetric cells, shown in Fig. S37 (SI), indicate that the ZnSe@PC-based cell has the lowest internal resistance and interfacial charge transfer impedance. As for the solid–liquid phase transition process, Li_2_S deposition and dissolution behaviors were examined. During the deposition of Li_2_S, additional Li_2_S generated from the disproportionation of LiPSs tends to deposit onto pre-existing Li_2_S particles. This preference is driven by the strong affinity between similar species, which is crucial for achieving a higher Li_2_S deposition capacity. Effective d–p hybridization between ZnX@PC and LiPSs significantly weakens the Li–S bond, thereby reducing the energy barrier for the reduction of LiPSs. As shown in the Li_2_S nucleation profiles (Fig. 4a and S38, SI), The tests revealed nucleation capacities of 353.73, 354.27, and 426.84 mAh g^−1^ for ZnO@PC, ZnS@PC, and ZnSe@PC, respectively. The corresponding peak appearance times were 6827, 4777, and 3458 seconds. ZnSe@PC exhibits the shortest response time and highest peak current, revealing superior electrocatalytic activity toward LiPSs. ZnSe@PC exhibits a nucleation capacity of 426.84 mAh g^−1^, significantly exceeding those of ZnO@PC and ZnS@PC. This demonstrates that ZnSe@PC is more favorable for facilitating Li_2_S deposition and substantially enhancing the nucleation kinetics of Li_2_S. To understand the differences in Li_2_S growth behavior on various surfaces, the current–time transients obtained from Li_2_S nucleation tests were analyzed using dimensionless diagnostic plots based on the Scharifker–Hills model (Fig. 4c).^68^ In this model, 3DI and 3DP correspond to three-dimensional instantaneous and progressive nucleation under diffusion control, respectively, while 2DI and 2DP represent two-dimensional nucleation governed by lattice incorporation. For ZnX@PC, Li_2_S growth follows a mixed 2DI/3DP mode, indicating that the abundant, monodisperse ZnX@PC sites promote instantaneous nucleation and dense deposition. The rapid precipitation of Li_2_S also helps maintain a sufficient concentration of dissolved active species in the electrolyte, thereby enabling subsequent three-dimensional growth. Fig. S39 (SI) reveals the post-deposition morphology of carbon-based materials. ZnO@PC and ZnS@PC electrodes exhibit irregular Li_2_S deposition with significant agglomeration. In contrast, ZnSe@PC demonstrates uniform and dense Li_2_S nucleation across its surface.

To further evaluate the redox catalytic activity of ZnX@PC, potentiostatic intermittent titration technique (PITT) was performed on coin cells. As shown in Fig. 4b, stepwise discharge tests were conducted at 50 mV intervals within the 2.35–1.8 V window, with current response monitored during each voltage step. ZnSe@PC consistently exhibits higher current transients during voltage transitions, indicating enhanced electrochemical reaction kinetics. Notably at 2.05 V, detectable current responses confirmed Li_2_S_8_ nucleation initiation, while significantly amplified currents at 2.0 V demonstrate superior catalytic capability for LiPSs-to-Li_2_S conversion. Parallel observations during the charging process (Fig. 4d) further corroborate these findings. Collectively, PITT analysis reveals that ZnSe@PC boosts redox kinetics, and promotes rapid solid–liquid phase transitions.

Moreover, the ZnSe@PC electrode demonstrates a high Li_2_S dissolution capacity of 774.62 mAh g^−1^ and the fastest current response, as evidenced in Fig. 4e and S40 (SI), indicating the promoted conversion from solid Li_2_S to soluble LiPSs by ZnSe@PC catalyst. A comprehensive and visually accessible comparison of the nucleation and dissolution capacities for various carrier materials is presented in Table S5. Fig. S41 (SI) further confirmed complete dissolution of Li_2_S on the ZnSe@PC electrode, whereas ZnO@PC and ZnS@PC electrodes exhibited varying degrees of Li_2_S aggregation. This indicates superior redox kinetics in the ZnSe@PC electrode.

The schematic diagram in Fig. 4f depicts the adsorption–diffusion–conversion process of LiPSs on the surfaces of ZnX@PC. ZnSe@PC combines the advantages including appropriate adsorption, excellent catalytic activity, and good conductivity, thus enabling rapid kinetics for the sulfur cathode. Fig. S42 (SI) shows the *Ak*^2^ values calculated using the formula *Ak*^2^ = 2/(π*tm*^3^) to compare the relative decomposition rates across samples. ZnSe@PC exhibits a substantially higher *Ak*^2^ value than both ZnO@PC and ZnS@PC, indicateing that the hierarchical porous structure with uniformly embedded ZnX nanoparticles provides abundant active sites, facilitating rapid Li_2_S deposition/dissolution and enhancined redox reaction kinetics.^69^

**Fig. 5:**
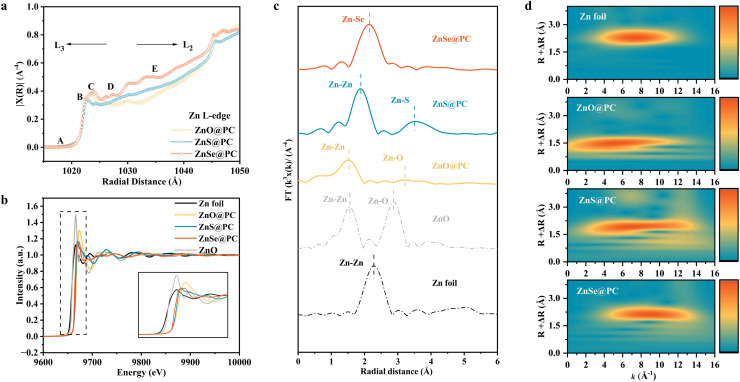
(a) Zn L-edge XAS spectra of ZnO@PC, ZnS@PC and ZnSe@PC. (b) Zn K-edge XANES spectra of Zn foil, ZnO@PC, ZnS@PC and ZnSe@PC. (c) FT of Zn foil, and ZnX@PC. (d) WT of Zn foil, and ZnX@PC weighted EXAFS signals.

As known, the chemical adsorption of LiPSs on metal-based hosts arises from d–p orbital hybridization in M–S bonds. The energy position of the Zn d-band center would be affected by the p-band characteristics of anions (O/S/Se) in ZnX, thus leading to the distinct LiPSs adsorption–catalysis behaviors in Li–S batteries. We have employed X-ray absorption spectroscopy (XAS) to investigate the electronic structure of anion-modified ZnX@PC materials, with particular focus on their valence states, symmetry variations, and local coordination environments (Fig. 5a). In Zn-based compounds, the 3d orbitals are fully occupied. Consequently, in accordance with Mott's selection rules, the Zn L_3_-edge exhibits pronounced sensitivity to electronic transitions from 2p to 4s and 4d states. The Zn L_3,2_-edge XAS spectra of various Zn-based materials exhibit characteristic features in the L_3_-edge (1012–1030 eV) and L_2_-edge (1030–1045 eV) regions. The L_3_-edge spectrum rises at 1014 eV, displaying four distinct peaks at ∼1017.9 eV (A), 1022.3 eV (B), 1023.9 eV (C), and 1027 eV (D). The L_2_ absorption edge follows the L_3_-edge, with its main peak (E) appearing at ∼1034.6 eV. Peak A, assigned to the pre-edge region, reflects crystal disorder and dipole-forbidden electronic transitions. Peaks B and C in the main-edge region correspond to transitions of 2p electrons to Zn 4p states, with their intensities indicating the density of available unoccupied states. Peak D (∼1027 eV, post-edge) suggests weak d–d interactions between the source and neighboring Zn 4d states at higher energy levels. Spectral analysis reveals that ZnSe@PC exhibits the most pronounced intensity for features C and D, while feature A remains invariant across modifications, indicating its insensitivity to anion substitution. Zn's 3d orbitals are fully occupied (3d^10^ configuration), making the 4s orbital the lowest unoccupied state, followed by 4p and 4d orbitals. Due to the localized nature of 3d orbitals relative to 4s states, ligand field splitting enhances the probability of Zn 2p → 3d transitions over Zn 2p → 4s transitions. Among anion-modified samples, ZnSe@PC shows enhanced C-peak intensity, with Se anions inducing asymmetric ligand field strengths at Zn 3d_t_2g__ and 3d_e_g__ orbitals.

Elucidating the coordination environment of catalysts is critical for understanding catalytic mechanisms, particularly regarding bond characteristics between central metal atoms and ligands, spatial arrangements of coordinating atoms, and electron transfer dynamics among active sites. X-ray absorption near edge structure (XANES) and extended X-ray absorption fine structure (EXAFS) spectroscopy were employed to analyze the coordination environments and chemical states of Zn atoms in ZnX@PC (Fig. 5b–d). The absorption edges of ZnSe@PC and ZnS@PC lie between those of metallic Zn foil (Zn^0^) and ZnO (Zn^2+^), indicating partial oxidation of Zn atoms with valence states between 0 and +2. ZnSe@PC's edge position nearest to metallic Zn suggests the enhanced metallicity and superior electrical conductivity. Magnified views reveal intensified pre-edge features in ZnSe@PC compared to ZnO@PC and ZnS@PC, reflecting distinct coordination environments. These pre-edge K-shell transitions, sensitive to anion-dependent coordination geometries, correlate with observed electrochemical enhancements. Specifically, Se anion modification elevates catalytic activity through optimized electronic configurations. This energy modulation reduces the activation barrier for sulfur redox reactions, thereby accelerating reaction kinetics. Fourier-transformed EXAFS spectra (*k*^3^-weighted, *R*-space) in Fig. 5c elucidates structural details. ZnSe@PC exhibits a primary Zn–Se peak at 2.14 Å, while ZnS@PC and ZnO@PC show Zn–S (3.49 Å) and Zn–O (3.1 Å) coordination shells, respectively. Wavelet-transform EXAFS contour plots (Fig. 5d) show maximum intensities at: 9.2 Å^−1^ (ZnSe), 7.69 Å^−1^ (ZnS), and 5.0 Å^−1^ (ZnO), demonstrating increasing *k*-space resolution along the O → S → Se series. These variations correspond directly to decreasing electronegativity from oxygen (3.44) to sulfur (2.58) and selenium (2.55). These above results clearly demonstrate the precise modulation on the electronic structure of Zn 3d orbital by anion components (X = O, S, Se), which finally affects the polysulfide adsorption–catalysis ability of ZnX@PC catalysts.

**Fig. 6:**
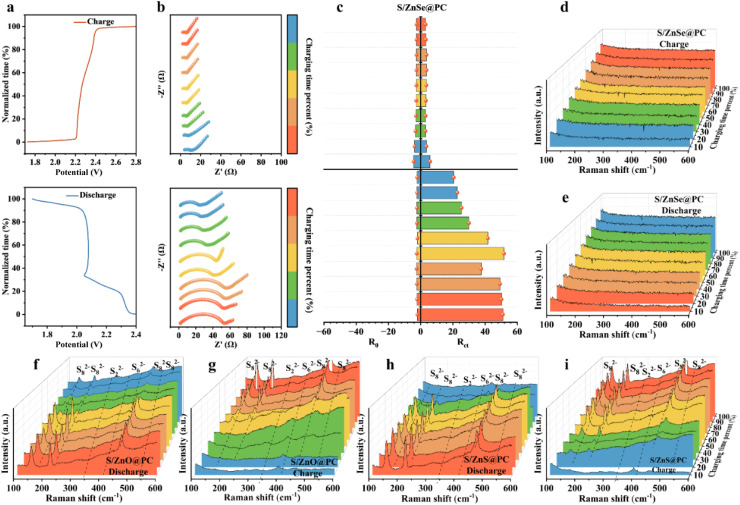
(a) Corresponding charge/discharge curves, and (b) *in situ* EIS measurements of S/ZnSe@PC cathode. (c) Variation in *R*_o_ and *R*_ct_ during discharging and charging process. *In situ* Raman spectroscopy analysis of (d and e) S/ZnSe@PC, (f and g) S/ZnO@PC, and (h and i) S/ZnS@PC.

Understanding impedance evolution during charge/discharge cycles is crucial for developing high-performance Li–S batteries. To analyze impedance evolution in S/ZnX@PC cells, *in situ* EIS was performed during constant-current cycling at 0.5C. Fig. 6a presents the galvanostatic charge–discharge profile corresponding to the *in situ* EIS test of the ZnSe@PC composite. Nyquist plots of S/ZnX@PC cells were presented in Fig. 6b, S43 and S44, and fitted by using the equivalent circuits (Fig. S45, SI). The results reveal the progressively increasing values of ohmic resistance (*R*_0_) and charge-transfer resistance (*R*_ct_) in cells with ZnO@PC and ZnS@PC cathode. In contrast, ZnSe@PC-based cell exhibits minimal resistance variations (Fig. 6c), demonstrating superior cycling reversibility. More specifically, S/ZnSe@PC cell maintains consistently low *R*_0_ values throughout both charge and discharge processes. This indicates enhanced electronic conductivity at the cathode–electrolyte interface and effective suppression of polysulfide dissolution into the electrolyte in ZnSe@PC-based cells. Meanwhile, ZnSe@PC-based cells reach their maximum *R*_ct_ at ≈50% depth of discharge (DOD), with subsequent decrease as cycling progresses. Comprehensive parameters are provided in Tables S6 and S7 (SI). Notably during discharge, the volume expansion from S to Li_2_S formation substantially degrades conductive interfaces. This induces a progressive increase in charge-transfer resistance (*R*_ct_). However, ZnSe@PC-based cells exhibit declining *R*_ct_ after reaching peak values. This demonstrates ZnSe@PC's efficacy in regulating Li_2_S deposition/growth, thereby suppressing volume expansion and enabling rapid charge transfer.

To directly monitor polysulfides during cycling, as illustrated in Fig. S46, *in situ* Raman spectroscopy was performed on S/ZnX@PC (X = O, S, Se) batteries, with spectra shown in Fig. 6d–i and S47–S49 (SI). Distinct peaks at 151, 218, and 471 cm^−1^ are detected in ZnO@PC and ZnS@PC-based cells. During discharge, S_8_^2−^ characteristic peaks are progressively replaced by S_6_^2−^/S_4_^2−^ signatures near 400 cm^−1^. Persistent polysulfide signals throughout discharging suggest insufficient adsorption–catalytic capabilities of ZnO@PC and ZnS@PC. By contrast, ZnSe@PC cell shows no detectable polysulfide peaks during cycling, indicating enhanced active material utilization. This confirms ZnSe@PC significantly promotes LiPSs conversion kinetics and active material utilization.

## 3 Conclusions

In summary, this study designed and synthesized zinc-based compounds encapsulated in nitrogen-doped porous carbon (ZnX@PC) using ZIF-8 as the precursor. Synchrotron radiation analysis and theoretical calculations confirm that Se anions induce a significant upward shift of the Zn d-band center toward the Fermi level in ZnSe, compared to ZnO and ZnS. Systematic analysis have revealed how p-band characteristics of anions influence the 3d orbital electronic structure of Zn active centers, thus modulating the d–p orbital hybridization between Zn sites in ZnX and S site of polysulfides. Besides, the hierarchical porous carbon matrix synergized with polar ZnX catalytic sites, provides abundant chemical anchoring sites for polysulfides, and optimizes Li^+^ diffusion kinetics. These advantages endowed ZnSe@PC with reduced energy barriers for LiPSs decomposition/conversion, accelerating Li_2_S deposition rates. *In situ* Raman spectroscopy further verified the moderate LiPSs adsorption strength and efficient catalytic conversion capability of ZnSe. Electrochemical tests demonstrated that the pouch cell based S/ZnSe@PC cathode delivers a reversible areal capacity of 953.3 mAh g^−1^ at 0.1C with sulfur loading of 7.0 mg cm^−2^ and electrolyte/sulfur ratio of 4.0 µL mg^−1^. This work propose an in-depth insight into the modulation of the electronic structure and catalytic ability of active metal sites *via* anion engineering in advanced Li–S batteries.

## Author contributions

Z. Z., C. H. and L. F. Z. conceived and designed the experiments. C. H., L. F. Z. and J. F. performed the measurement, collected data and analyzed data. X. Z. M., H. F. L., and Y. L. W. participated in the DFT calculations. Z. F. Z., Y. H. D., X. N. L., H. L., S. L., J. Y. and J. X. C. disscussed data. C. H., L. F. Z. and Z. Z. wrote the initial manuscript. L. F. Z., T. Y. M., and Z. Z. contributed to the revision of the manuscript. Z. Z. funding acquisition, supervision, project administration.

## Conflicts of interest

There are no conflicts to declare.

## Supplementary Material

SC-017-D6SC03397K-s001

SC-017-D6SC03397K-s002

SC-017-D6SC03397K-s003

SC-017-D6SC03397K-s004

## Data Availability

The data supporting this article have been included as part of the supplementary information (SI). Supplementary information is available. See DOI: https://doi.org/10.1039/d6sc03397k.
