# Supplementary material for: Electronic structure engineering of Zn-based catalysts via anionic regulation for polysulfide adsorption–catalysis in Li–S batteries
Source: Chem Sci. 2026 Jun 4;17(28):14041–53. doi: 10.1039/d6sc03397k (PMC13256313; doi:10.1039/d6sc03397k)
Supplement: SC-017-D6SC03397K-s001 [file SC-017-D6SC03397K-s001.pdf]

Supplementary Information (SI) for Chemical Science.
This journal is © The Royal Society of Chemistry 2026

Supporting Information

Electronic structure engineering of Zn-based catalysts via anionic regulation for polysulfide adsorption-catalysis in Li-S batteries

Cheng He[1], Lingfeng Zhu[2,3,], Jia Fu[1], Xiangzeng Meng[1], Hongfu Liu[1], Youliang Wang[1], Zhenfang Zhang[2,3], Xiaoning Li[2,3], Hui Li[2,3], Tianyi Ma[2,3,*], Sheng Liu[4], Yuhai Dou[5], Ji Yu[1], Jianxin Cai[1], Ze Zhang[1,*]*

[1]School of Chemistry and Chemical Engineering, Key Laboratory Foundation of Jiangxi Province for Environment and Energy Catalysis, Nanchang University, Nanchang 330031, China

[2]Centre for Atomaterials and Nanomanufacturing (CAN), School of Science, RMIT University, Melbourne, VIC 3000, Australia.

[3]ARC Industrial Transformation Research Hub for Intelligent Energy Efficiency in Future Protected Cropping (E2Crop), Melbourne, VIC 3000, Australia

[4]School of Materials Science and Engineering, Nankai University, Tianjin 300350, China.

[5]Centre for Catalysis and Clean Energy, Gold Coast Campus, Griffith University, Gold Coast 4222, Australia

* Corresponding authors: lingfeng.zhu@rmit.edu.au (L. Zhu); tianyi.ma@rmit.edu.au (T. Ma) and zhangze@ncu.edu.cn (Z. Zhang)

1 Experimental Section

1.1 Preparation of ZnSe@PC, ZnS@PC, and ZnO@PC

2.23 g of zinc nitrate and 2.11 g of dimethylimidazole were separately dissolved in 100 mL of methanol, and the two solution were then mixed and stirred until the color is uniform. The above mixture was kept for 24 hours. After centrifugation, the precipitate was dried in a 60°C oven to obtain ZIF-8. ZIF-8 was firsly heated in a nitrogen atmosphere to 600°C at a rate of 3°C min^{-1} and hold for 2 hours to obtain Zn-C precursor. Then, the powdered Zn-C was mixed with Se powder in a mass ratio of 1:2 with Se powder placing in the upstream position and Zn-C in the downstream position in the furnace. The mixture was heated in a nitrogen atmosphere to 700°C at a rate of 3°C min^{-1} and hold for 2 hours to obtain ZnSe@PC. By replacing the Se powder with sulfur powder, ZnS@PC was abtained via the same procedure. For ZnO@PC sample, Zn-C precursor was placed in the furnace, heated in air at a rate of 3°C min^{-1} to 400°C, and hold for 2 hours to obtain ZnO@PC.

1.2 Preparation of S/ZnSe@PC, S/ZnS@PC, and S/ZnO@PC

The prepared ZnX@PC (X= O, S, Se) was mixed with sublimated sulfur in a mass ratio of 1:3, ground thoroughly in a mortar, and then heated in a nitrogen atmosphere to 155°C at a rate of 3°C min^{-1} and hold for 12 hours to obtain S/ZnX@PC composites.

1.3 Preparation of composite cathode sheets

The S/ZnX@PC composites, conductive agent (Super P), and binder (PVDF) were

mixed in a mass ratio of 7:2:1. An appropriate amount of N-methyl-2-pyrrolidone (NMP) was added and thoroughly ball-milled for 6 hours to obtain a homogeneous, viscous slurry. Subsequently, the slurry was uniformly coated onto a carbon-coated aluminum foil using a spatula and dried in vacuum at 60°C for 12 hours. Finally, a punch machine was applied to cut the cathode sheets into disks with a diameter of 12 mm. The active sulfur content was approximately of 1.0-1.2 mg cm^{-2}.

1.4 Preparation of lithium-sulfur electrolyte

The electrolyte preparation was carried out entirely inside an argon-filled glovebox (with water and oxygen levels both below 0.5 ppm). Accurately weigh 2.870 g of lithium bis(trifluoromethanesulfonyl)imide (LiTFSI) and 0.200 g of $LiNO_3$ and place them in a 20 mL transparent glass bottle. Then, add 5 mL each of 1,3-dioxolane (DOL) and 1,2-dimethoxyethane (DME). Seal the bottle with Parafilm and stir it on a constant-temperature shaker at 30°C for 12 hours until a clear electrolyte is obtained (LiTFSI concentration of 1 mol L^{-1}).

1.5 Assembly of the battery

The assembly was conducted entirely within an argon-filled glovebox. The prepared cathode, separator, electrolyte, lithium metal foil, nickel foam and anode/cathode shells were assembled into CR2025 or CR2032 coin cells and sealed using a battery crimping machine. Allow the battery to stand for 5 to 6 hours before conducting subsequent electrochemical tests to ensure that the electrolyte fully wets the electrodes.

1.6 Structural Characterizations

The morphology and elemental composition of the samples were analyzed using scanning electron microscopy (SEM, ZEISS SUORA®55) equipped with energy dispersive spectroscopy (EDS) and transmission electron microscopy (TEM, Tecnai G2 F20). X-ray photoelectron spectroscopy (XPS, ThermoFisher Scientific, ESCALAB250 Xi) was carried out to characterize the chemical compositions of the materials. X-ray diffraction (XRD, Bruker D8 ADVANCE) was conducted to analyze the phase purity of the samples in the 10°-80° (2θ) range at a scan speed of 4° min^{-1}. The nitrogen adsorption-desorption isotherms were demonstrated using a surface area analyzer (JW-BK100C). The absorbance of Li_2S_6 solutions was measured using a UV-Vis spectrophotometer (Agilent, G6860A03040425).

1.7 Constant-current discharge/charge testing

The assembled batteries were subjected to constant-current discharge/charge tests using the Neware CT-3008 battery testing system. The electrochemical performance, including rate capability, cycle stability, polarization behavior, and Coulombic efficiency at different current densities (0.1 to 5 C, 1 C = 1675 mA g^{-1}), was evaluated. The testing conditions were: a voltage range of 1.7 to 2.8 V and an ambient temperature of 25°C.

1.8 Cyclic voltammetry (CV)

Using a Donghua DH7000C electrochemical workstation, current-voltage curves were

recorded in the voltage range of 1.7 to 2.8 V at different scan rates (0.1 to 0.5 mV s^{-1}). The cyclic voltammetry (CV) curves record the various electrochemical reactions occurring within the battery system and are used to analyze the mechanisms of these electrochemical reactions.

1.9 Electrochemical Impedance Spectroscopy (EIS)

EIS is a technique that involves applying a small amplitude alternating voltage or current to perturb the electrode for electrochemical testing. The obtained impedance data can be used to calculate the corresponding electrode reaction parameters based on a model of the electrode's equivalent circuit. This study used a PARSTAT-2273 electrochemical workstation from Princeton Applied Research. The frequency range for testing was 100 mHz to 100 kHz, with an amplitude of 5 mV.

1.10 Nucleation and dissolution of Li_2S

The dispersion of the above samples (1 mg) in ethyl alcohol (1 mL) was dropped onto a piece of carbon paper (diameter in 12 mm). The material-loaded carbon paper was used as the cathode to build coin cells coupled with Li anode. The catholyte was 20 μL of Li_2S_8/tetraglyme solution (0.5 M of S) and the anolyte was 20 μL of 1 M LiTFSI/tetraglyme. The cells were galvanostatically discharged to 2.06 V at 0.112 mA current and then potentiostatically held at 2.05 V until the discharge current was less than 0.01 mA for full Li_2S nucleation. In order to investigate the process of Li_2S dissolution, the cells were first discharged to 1.8 V at 0.112 mA and further to 1.7 V at 0.01 mA for full conversion of LiPSs into solid Li_2S. The cells were subsequently

potentiostatically charged at 2.4V until the charge current was less than 0.01 mA to dissolve Li_2S into LiPSs.

1.11 Adsorption and soaking tests

For the adsorption test, 20 mg samples were soaked in 3 mL Li_2S_6 solution (10 mmol L^{-1}). The Li_2S_6 solution were prepared according to the reaction Equation S1:

$$5S + Li_2S \leftrightarrow Li_2S_6 \qquad (S1)$$

Using a solvent mixture of 1,3-dioxolane (DOL) and dimethoxyethane (DME) (1:1 in volume). UV/Vis spectra of the above solutions (diluted 5 times before testing) were recorded by using a UV2550 instrument (Shimadzu, Japan). The concentration variations in these solutions was detected by the UV/Vis spectrocopy.

1.12 In-Situ Raman Measurements

The assembly process of the cell for in-situ Raman testing was similar to that of the cells used for electrochemical testing, except for the use of a Li foil with a small hole as the anode and an anode shell with a mica plate serving as the laser window. Raman spectroscopy analysis was conducted using Renishaw inVia equipment, while galvanostatic charge–discharge measurements were performed on a Neware battery test system. A laser emitting at 633 nm wavelength and equipped with a 50x objective lens (Leica) was employed. Raman spectra were collected at 2.30, 2.10, 2.05 and 1.70 V, with each spectrum acquisition lasting for 10 s within the range of 100-600 cm^{-1}.

2. Calculations

All the Density functional theory (DFT) calculations were conducted based on the Vienna Ab initio Simulation Package (VASP).[1, 2] The exchange-correlation potential was described by the Perdew−Burke−Ernzerhof (PBE) generalized gradient approach (GGA).[3] The electron-ion interactions were accounted by the projector augmented wave (PAW).[4] All DFT calculations were performed with a cut-off energy of 400 eV and the 3×3×1 Monkhorst-Pack grid k-points were selected to sample the Brillouin zone integration. The energy and force convergence criteria of the self-consistent iteration were set to 10-4 eV and -0.02 eV $Å^{-1}$, respectively. DFT-D3 method is adopted to describe the van der Waals interaction.[5]

3. Supplementary figures

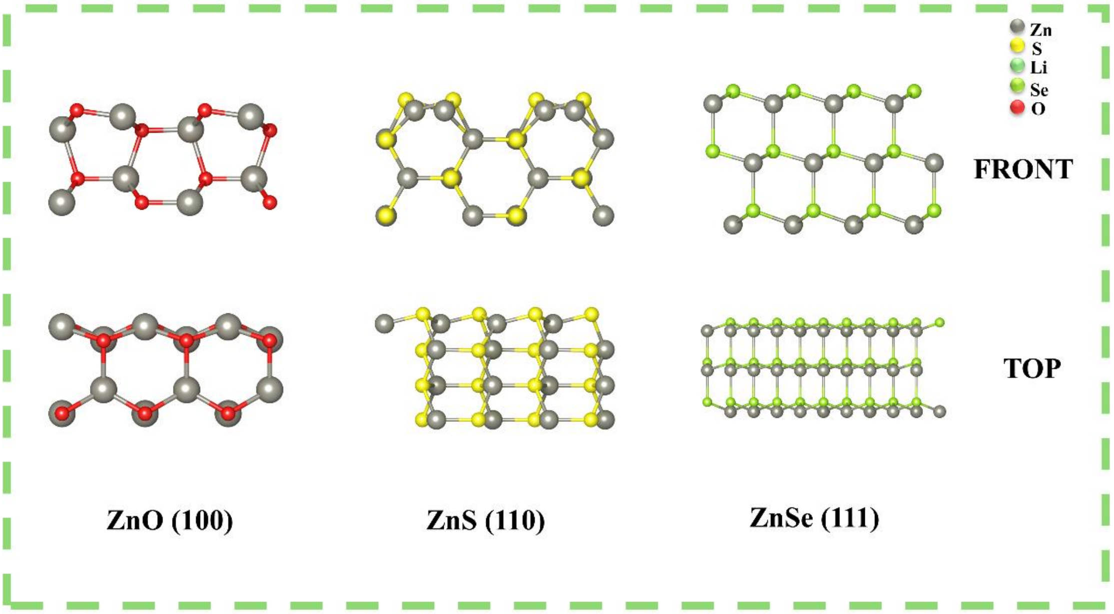

Figure S1. Structural models of the ZnO (100), ZnS (110), and ZnSe (111) surfaces from top and front perspectives. Color scheme: Zn (gray), O (red), S (yellow), Se (light green).

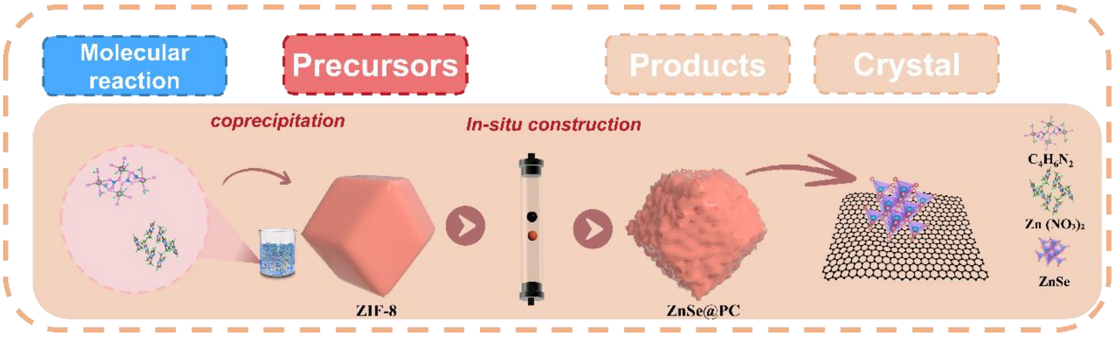

Figure S2. Schematic representation of the synthesis process of ZnSe@PC.

As shown in **Figure S2**, ZIF-8 powder was successfully synthesized via a classic mixed-and-resting method. Subsequent in-situ calcination and selenization yielded ZnSe@PC materials, which retained well-defined dodecahedral morphology and inherited the high surface area and porous structure characteristic of MOF-based materials.

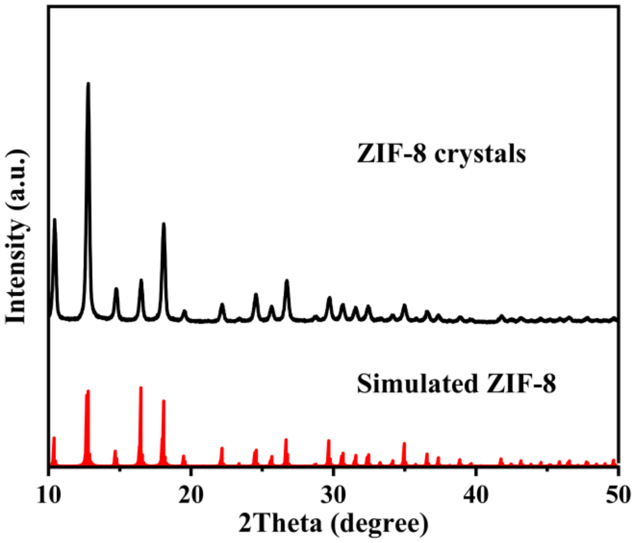

Figure S3. XRD pattern of ZIF-8 precursor.

The powder XRD patterns of all samples are shown in **Figure S3**. The XRD pattern of ZIF-8 shows that there are strong diffraction peaks at $2\theta = 7.4$, 10.4, and 12.7°, corresponding to multiple planes such as (011), (002), and (112), respectively, exhibits characteristic peaks consistent with those reported for ZIF-8 in the literatures. The crystal structure of ZIF-8 shows a body-centered cubic crystal lattice of the space group $I\bar{4}3m$.[6]

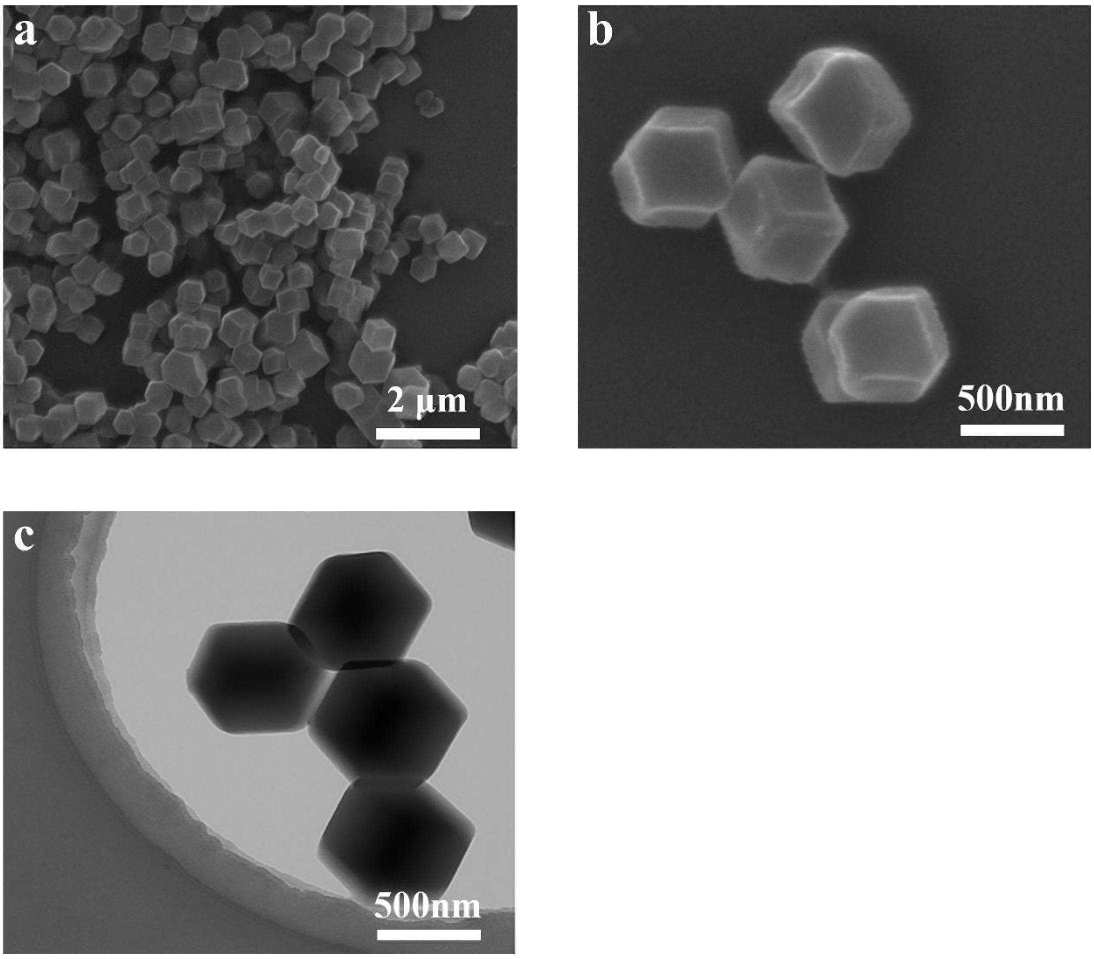

Figure S4. (a-b) SEM images of ZIF-8 with different magnifications. (c) TEM image of carbon cubic shell ZIF-8.

The SEM image (**Figure S4a**) and TEM image (**Figure S4b, c**) show the typical uniform polyhedron morphology of the as-synthesized ZIF-8 precursor, and the size is ~500 nm.

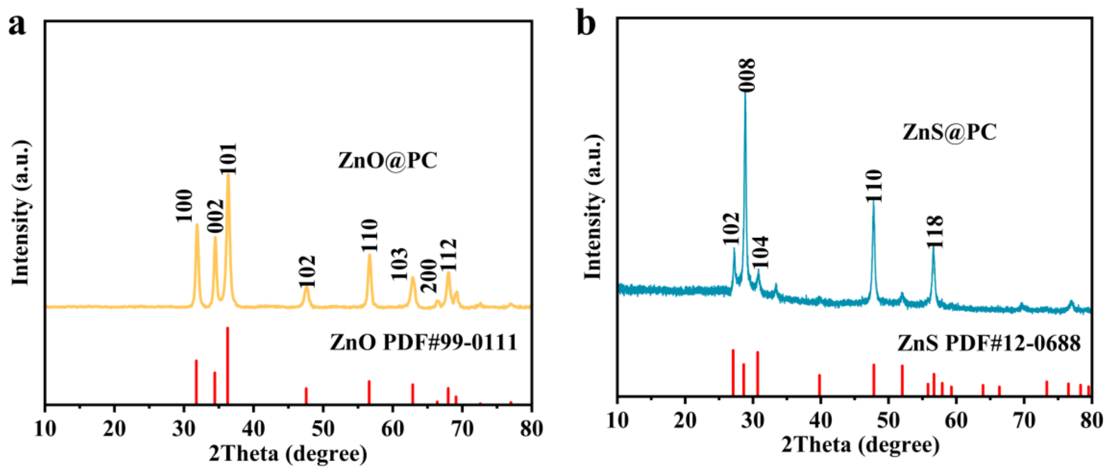

Figure S5. XRD patterns of (a) ZnO@PC and (b) ZnS@PC.

Figure S5a presents the X-ray diffraction (XRD) pattern of the ZnO@PC composite, all the obvious diffraction peaks could be indexed to the ZnO (PDF#99-0111). The X-ray diffraction pattern reveals well-defined peaks indexed to the (200) and (100) crystallographic planes, demonstrating a precise match with the corresponding standard powder diffraction file (PDF).[7] The XRD patterns of ZnS@PC are shown in **Figure S5b**. The clear diffraction peaks located at 27.2°, 29.0°, 30.68°, 47.9° and 56.5° can be indexed well to the (102), (008), (104), (110) and (118) crystal planes of standard cubic ZnS (PDF#12-0688).[8]

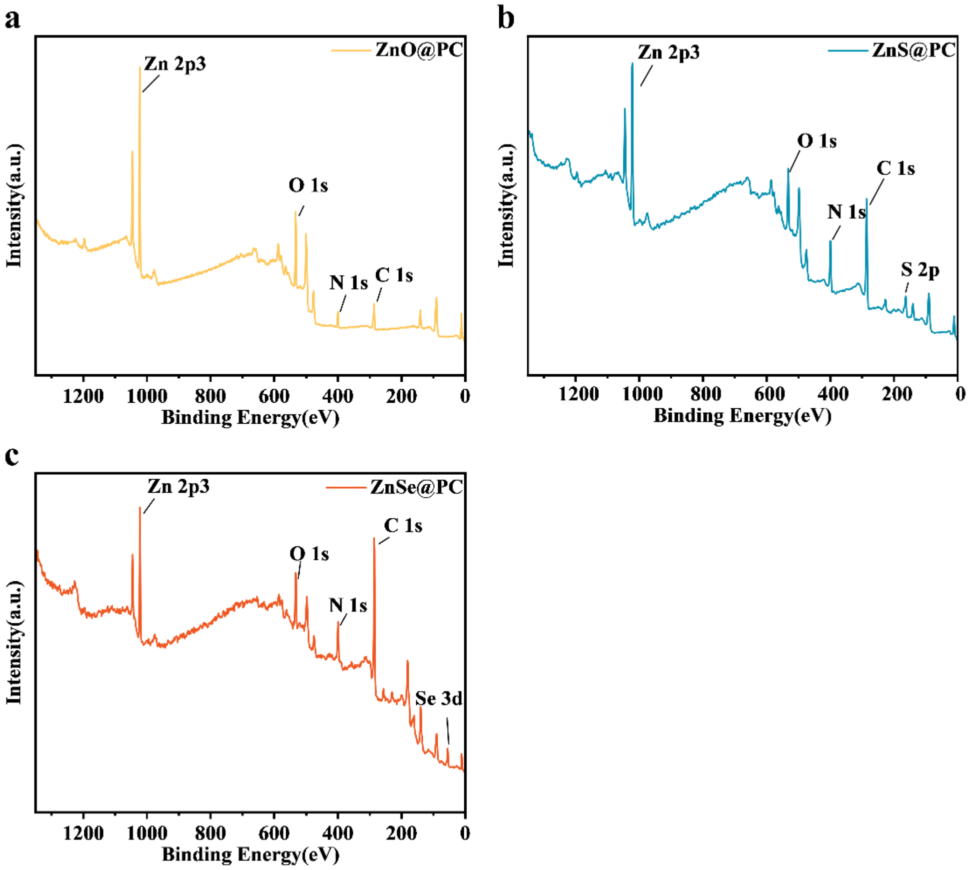

Figure S6. XPS surveys of (a) ZnO@PC, (b)ZnS@PC and (c) ZnSe@PC.

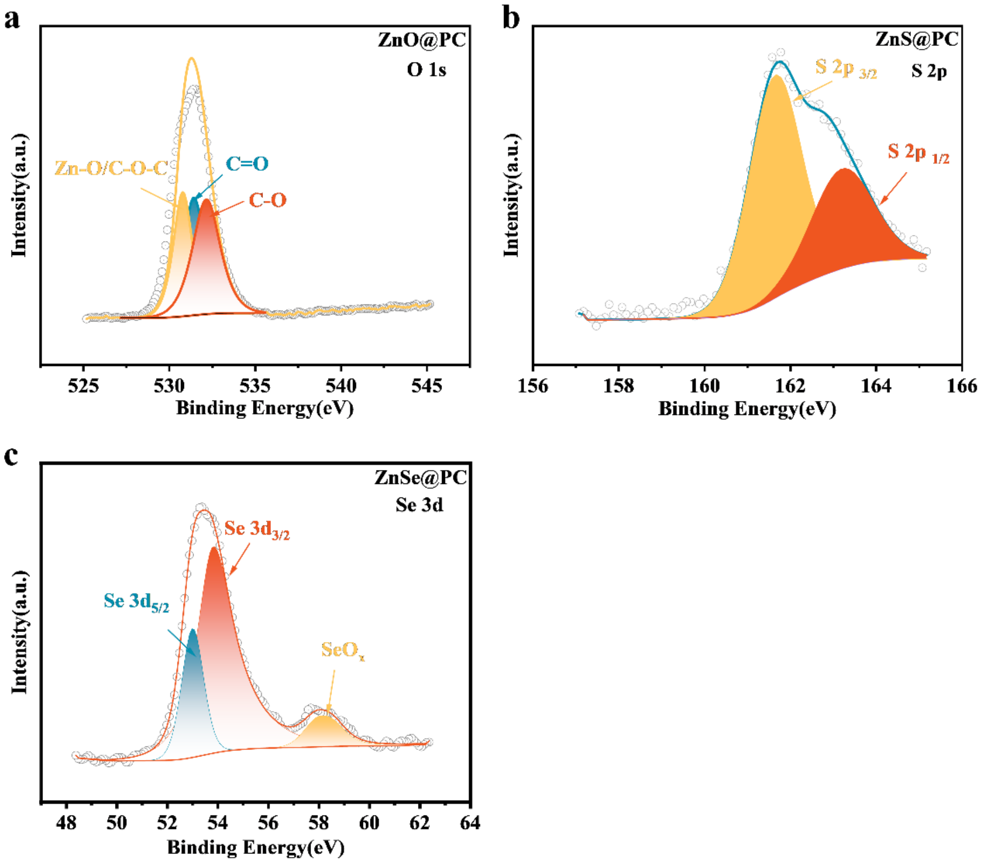

Figure S7. XPS surveys of (a) ZnO@PC'O 1s, (b) ZnS@PC'S 2p and (c) ZnSe@PC' Se 3d.

X-ray photoelectron spectroscopy (XPS) was employed to further elucidate the elemental composition and chemical bonding states of the samples. The high-resolution spectra in **Figure S6a-c** confirm the presence of characteristic elements, displaying the O 1s, S 2p and Se 3d regions for the different samples. **Figure S7a, b** presents the high-resolution O 1s and S 2p spectra. The O 1s spectrum for the ZnO@PC composite (**Figure S7a**) was deconvoluted into three peaks at 530.7 eV, 531.9 eV and 532.2 eV, corresponding to Zn-O, C=O and C-O bonds, respectively.[9] This provides strong evidence for the successful preparation of the material. The S 2p spectrum for ZnS@PC (**Figure S7b**) shows binding energies at approximately 161.1 eV and 163.5 eV, which are assigned to the S $2p_{3/2}$ and S $2p_{1/2}$ orbitals of ZnS, confirming the Zn-S interaction.[10]

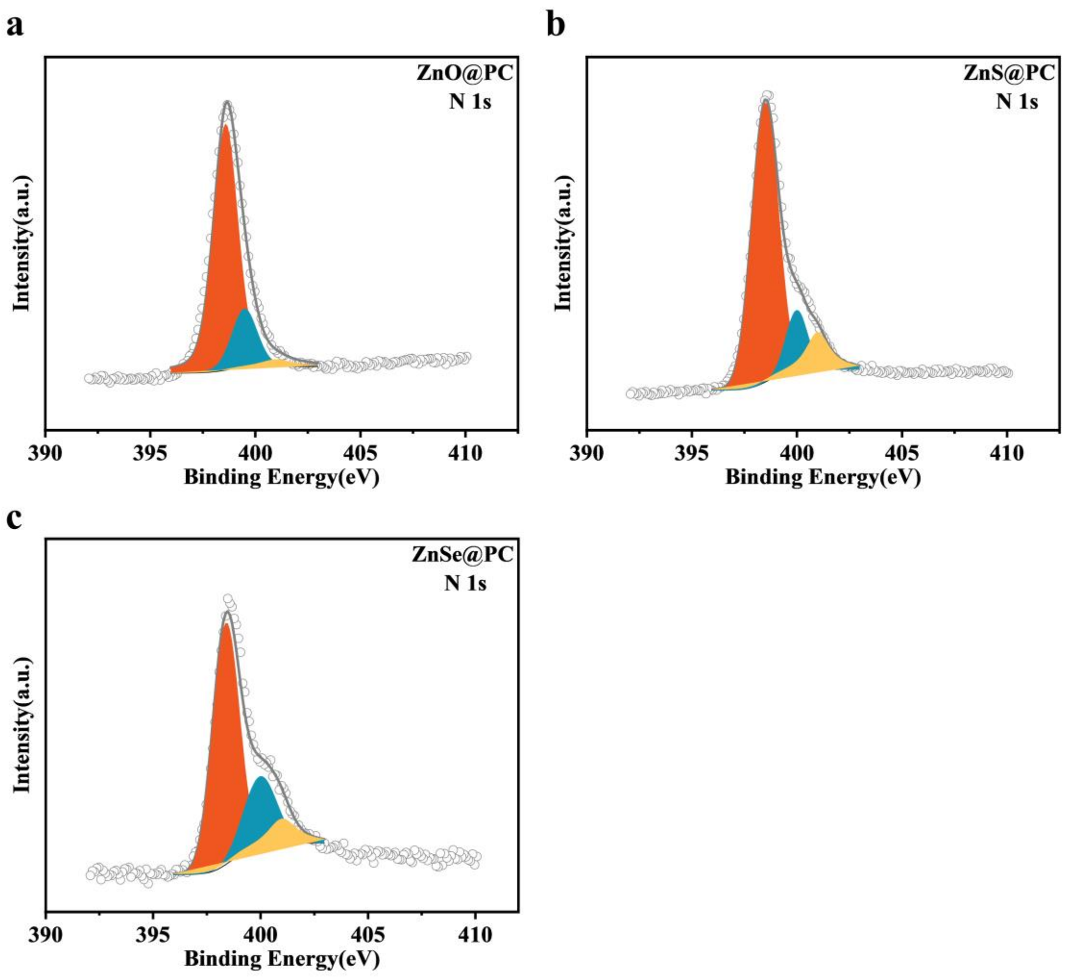

Figure S8. XPS spectrums of (a) ZnO@PC's N 1s, (b) ZnS@PC's N 1s and (c) ZnSe@PC's N 1s.

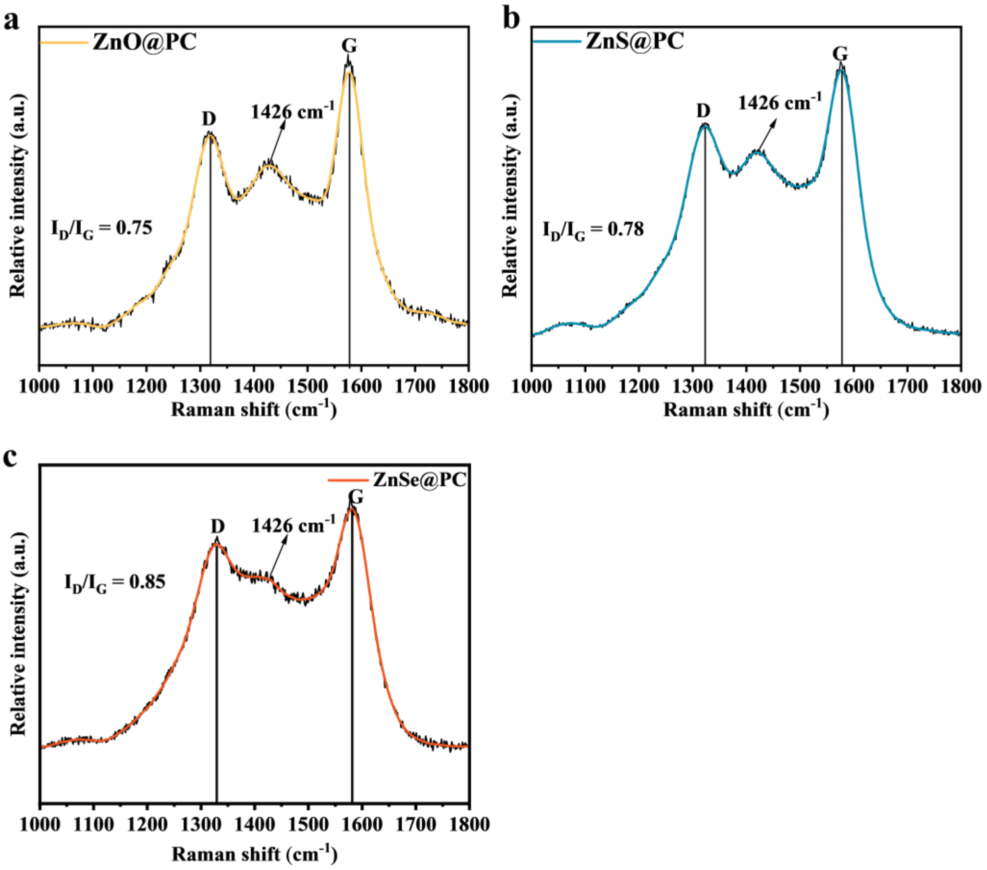

Figure S9. Raman spectra of (a) ZnO@PC, (b) ZnS@PC and (c) ZnSe@PC.

Raman spectra of both ZnO@PC, ZnS@PC and ZnSe@PC also exhibit a distinct broad peak at approximately 1426 cm^{-1}.[11] This feature is similarly attributed to the N-N stretching mode of the nitrogen-doped carbon matrix.

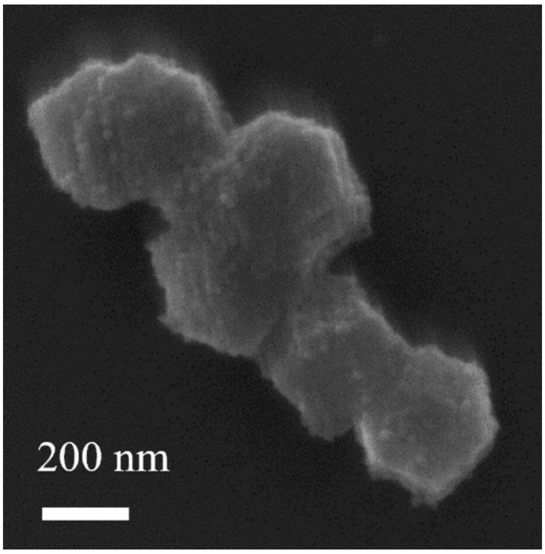

Figure S10. SEM image of ZnSe@PC.

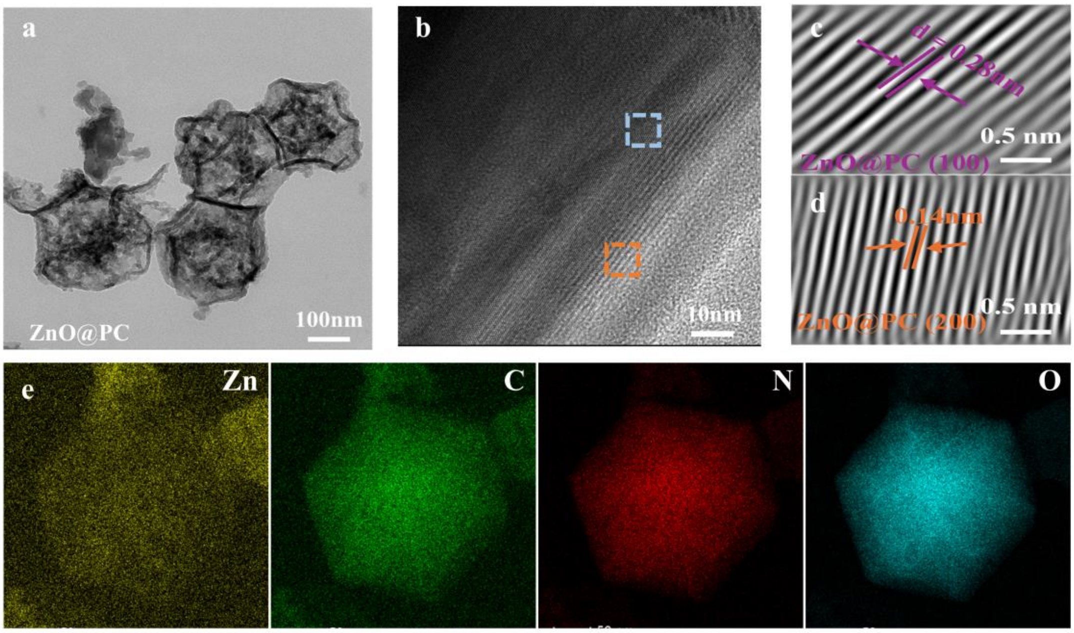

Figure S11. (a) SEM image of ZnO@PC. (b) TEM image of ZnO@PC. (c-d) HRTEM images of the lattices of ZnO@PC. (e) TEM image and EDX images of ZnO@PC.

TEM imaging (**Figure S11a, b**) reveals that ZnO nanoparticles are uniformly distributed along the internal walls of the nitrogen-doped porous carbon framework, with an average particle size of approximately 100 nm. HRTEM images (**Figure S11c, d**) display distinct lattice fringes with measured interplanar spacings of 0.28 nm and 0.14 nm, corresponding to the (100) and (200) planes of the cubic ZnO phase, respectively. Corresponding EDX elemental mapping (**Figure S11e**) further confirms the homogeneous distribution of Zn, O, C and N elements throughout the ZnO@PC composite, which aligns well with the observed material morphology.

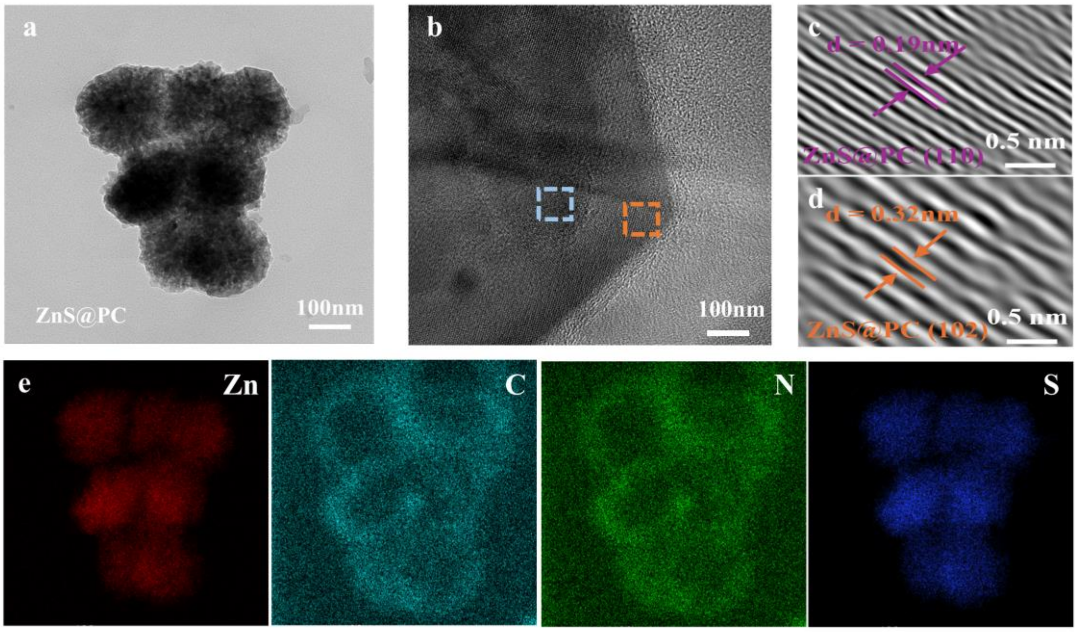

Figure S12. (a) SEM image of ZnS@PC. (b) TEM image of ZnS@PC. (c-d) HRTEM images of the lattices of ZnS@PC. (e) TEM image and EDX images of ZnS@PC.

TEM imaging (**Figure S12a, b**) clearly reveals the distribution of ZnS nanoparticles within the porous carbon framework. The particles exhibit uniform size and are homogeneously anchored to the internal walls of the nitrogen-doped porous carbon. This configuration effectively prevents ZnS agglomeration during cycling and provides continuous pathways for ion/electron transport. HRTEM images (**Figure S12c, d**) show distinct lattice fringes, confirming the high crystallinity of the ZnS nanocrystals. The measured interplanar spacings correspond well with the standard reference pattern for ZnS, unequivocally identifying its crystal phase. Corresponding EDX elemental mapping (**Figure S12e**) visually illustrates the spatial distribution of C, N, Zn and S elements within the composite. The highly overlapping and uniform signals of Zn and S confirm the successful formation of ZnS. The C signal outlines the three-dimensional porous skeleton of the entire composite. Importantly, the uniform distribution of the N signal indicates successful nitrogen doping into the carbon framework, which enhances

the electrical conductivity and surface chemistry of the carbon material. The high consistency in the spatial distribution of all elements demonstrates the successful construction of a compositionally uniform ZnS@PC composite.

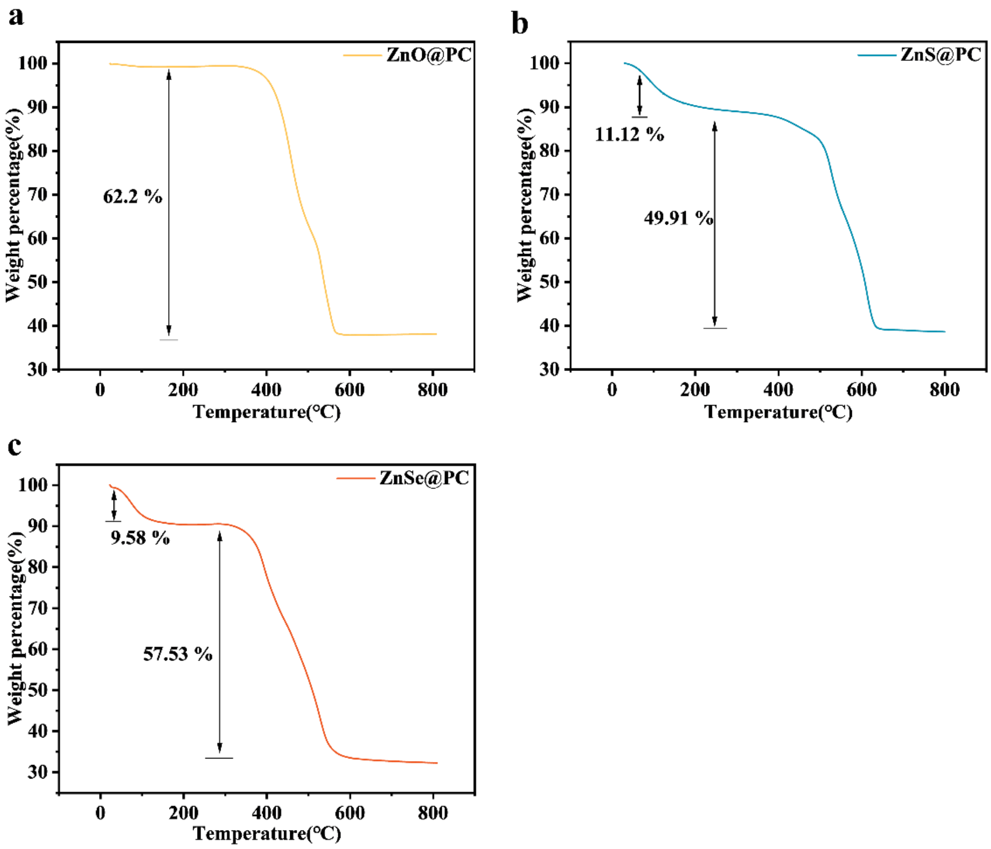

Figure S13. TGA curves of (a) ZnO@PC composite, (b) ZnS@PC composite and (c) ZnSe@PC composite.

When the composite was heated from 40°C to 800°C at air atmosphere, the ZnS@PC/ZnSe@PC would turn to ZnO, according to the following chemical reactions: C (s) + O_2 (g) → CO_2 (g); ZnSe (s) + O_2 (g) → ZnO (s) + SeO_2 (s); ZnS (s) + O_2 (g) → ZnO (s) + SO_2 (g).[12] The weight loss before 100°C was related to the evaporation of water in the samples.[12] Thus, the carbon content in the ZnS@PC and ZnSe@PC composites was calculated as:

$$\text{ZnS@PC:}\ \ \text{C wt\%} = \frac{1-\frac{M_{ZnS}}{M_{ZnO}}\times 49.91\%}{1-11.12\%} = 45.3\ \text{wt\%}$$

$$\text{ZnSe@PC:}\ \ \text{C wt\%} = \frac{1-\frac{M_{ZnSe}}{M_{ZnO}+M_{SeO2}}\times 57.53\%}{1-9.58\%} = 62.8\ \text{wt\%}$$

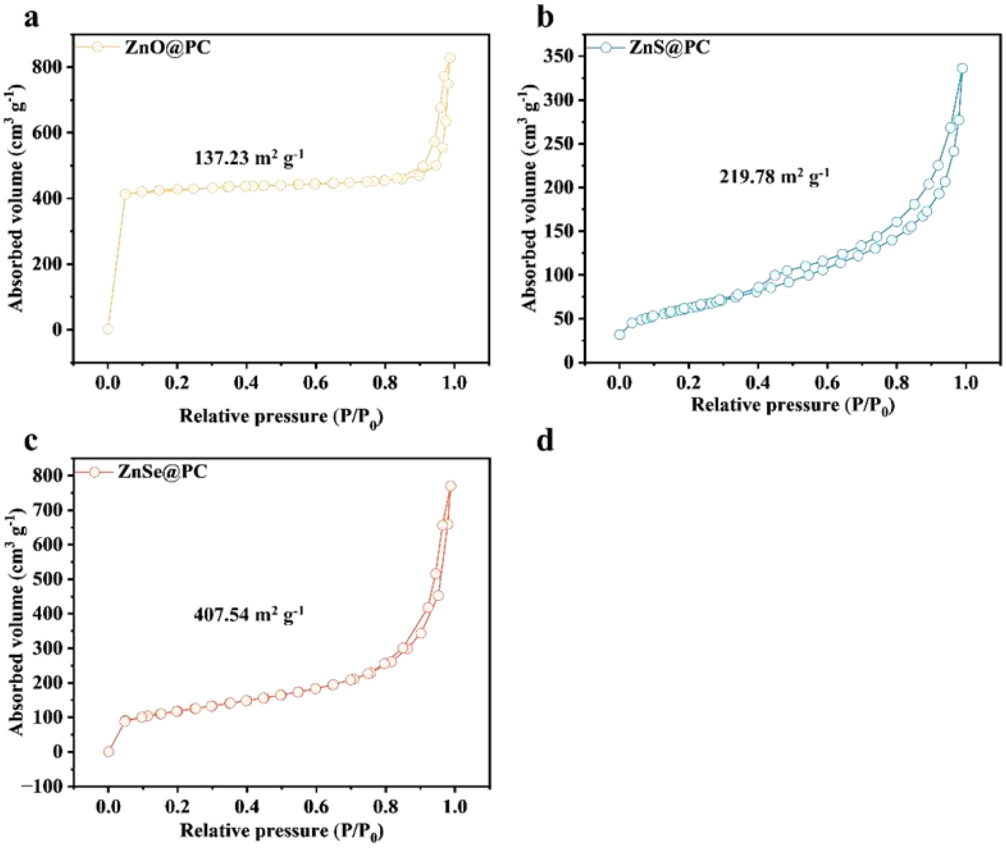

Figure S14. Nitrogen adsorption-desorption isotherms of (a) ZnO@PC, (b) ZnS@PC, (c) ZnSe@PC.

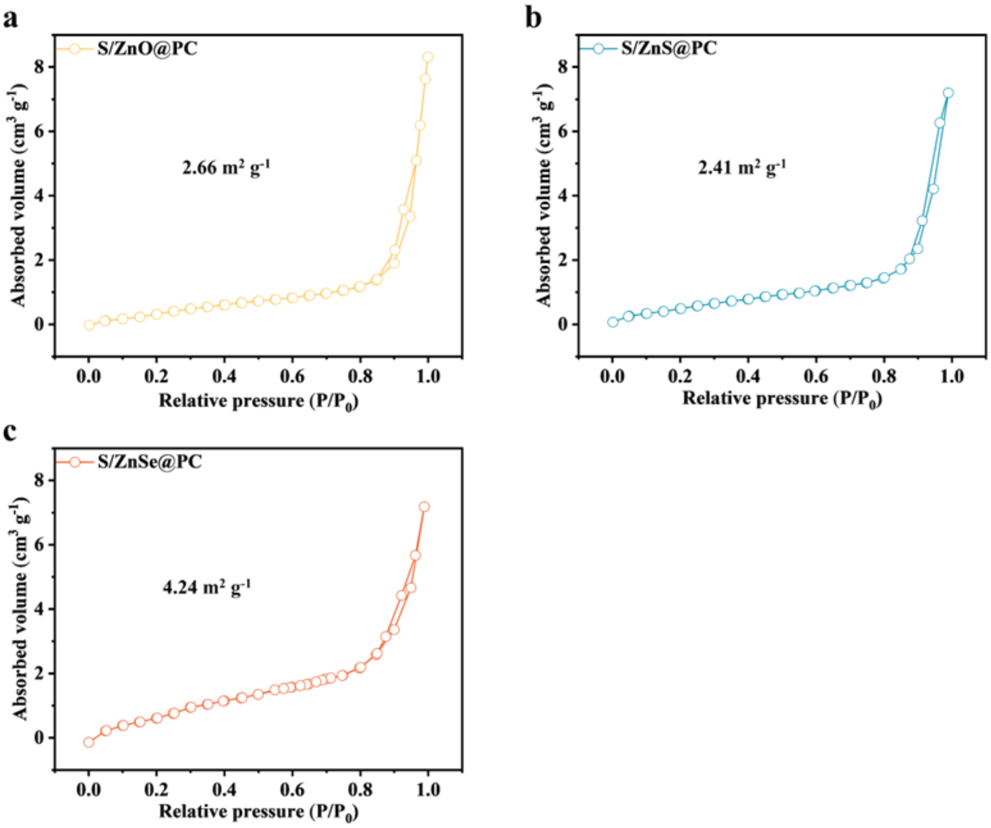

Figure S15. Nitrogen adsorption-desorption isotherms of (a) S/ZnO@PC, (b) S/ZnS@PC, (c) S/ZnSe@PC.

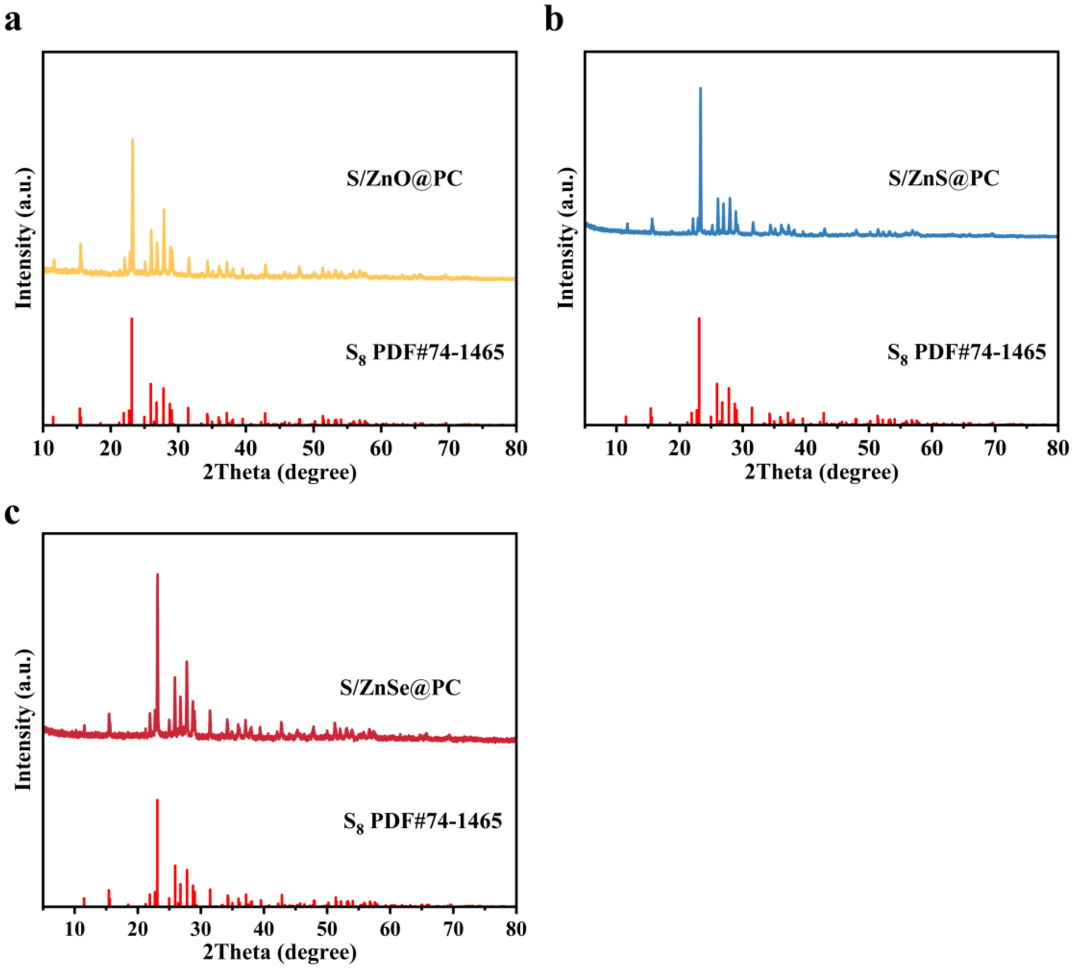

Figure S16. XRD patterns of (a) S/ZnO@PC composite, (b) S/ZnS@PC composite and (c) S/ZnSe@PC composite.

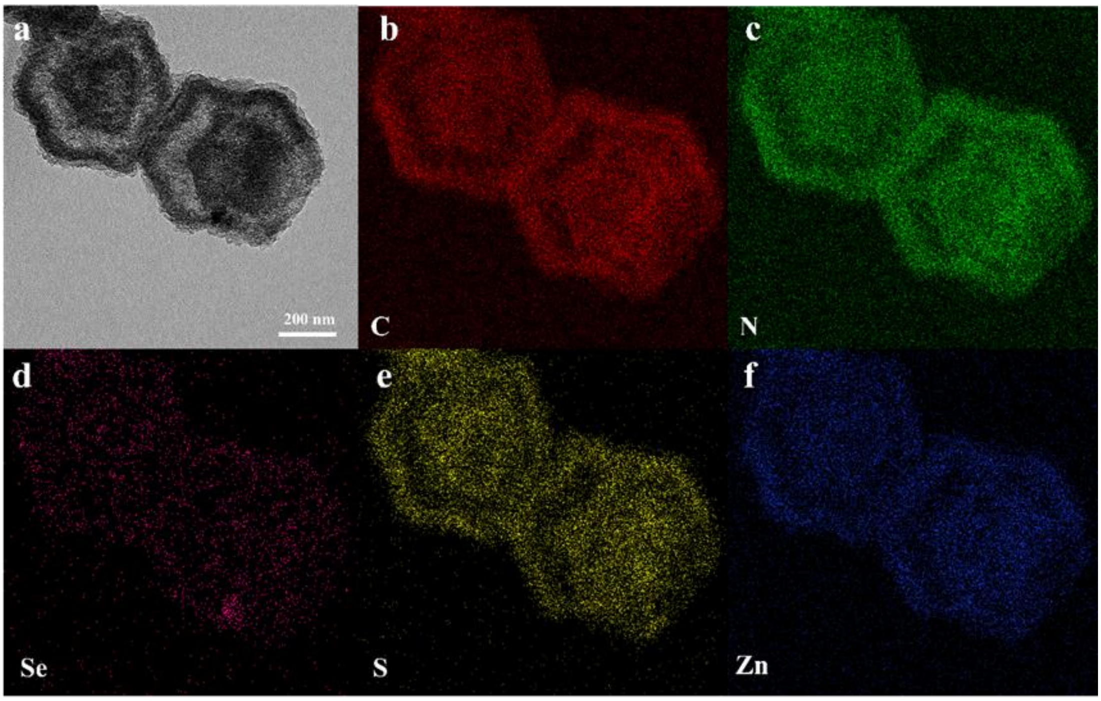

Figure S17. Microstructural Characterization of S/ZnSe@PC: (a) TEM and (b, c, d, f, g, h) EDS images.

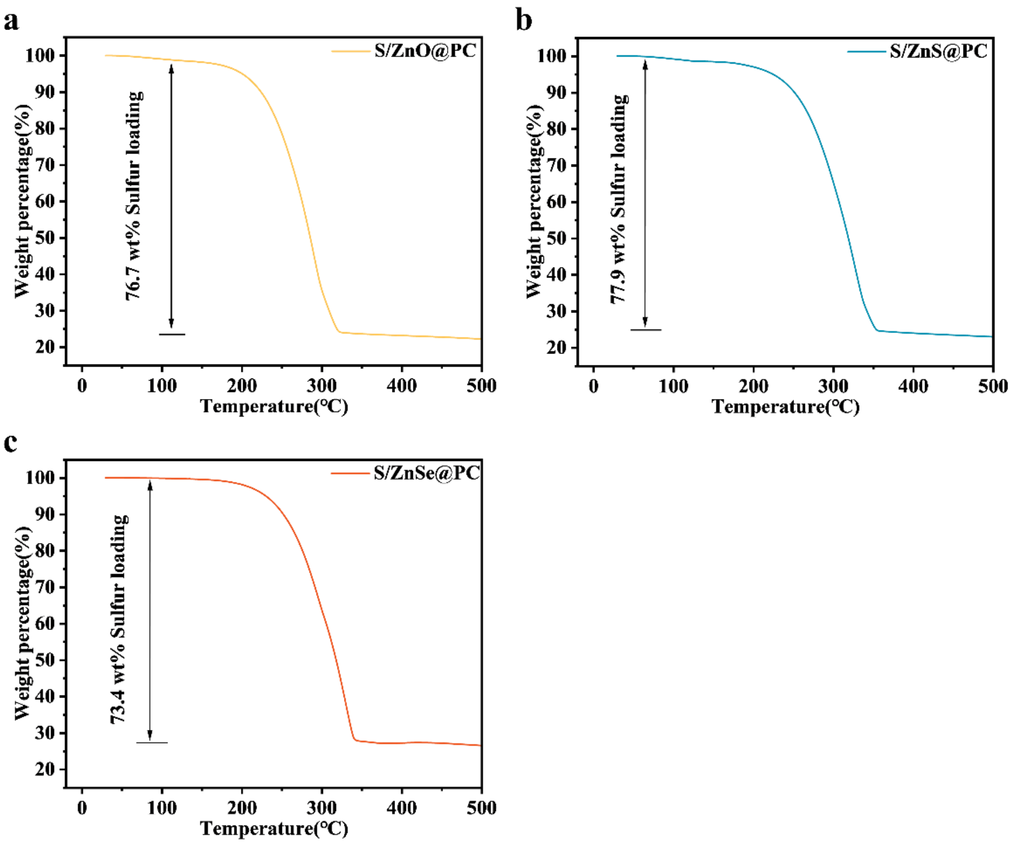

Figure S18. TGA curves of (a) S/ZnO@PC composite, (b) S/ZnS@PC composite and (c) S/ZnSe@PC composite.

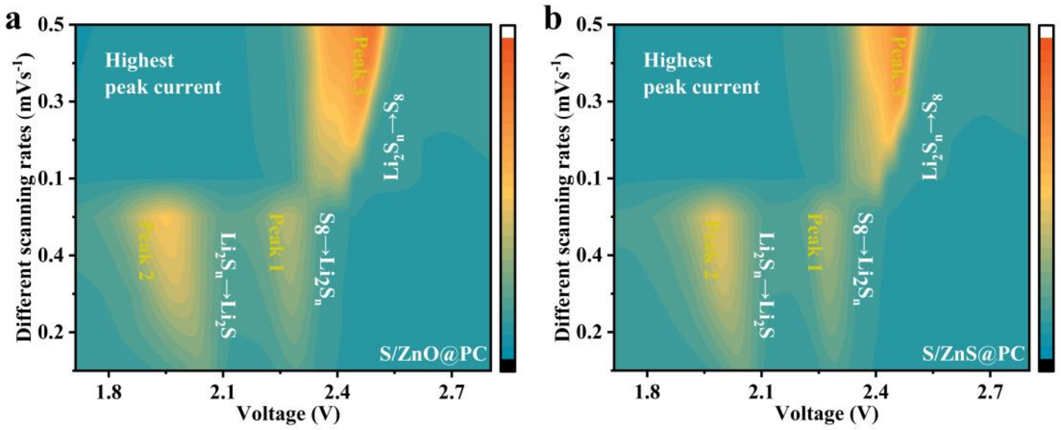

Figure S19. Contour plots of (a) CV patterns for S/ZnO@PC, (b) CV patterns for S/ZnS@PC.

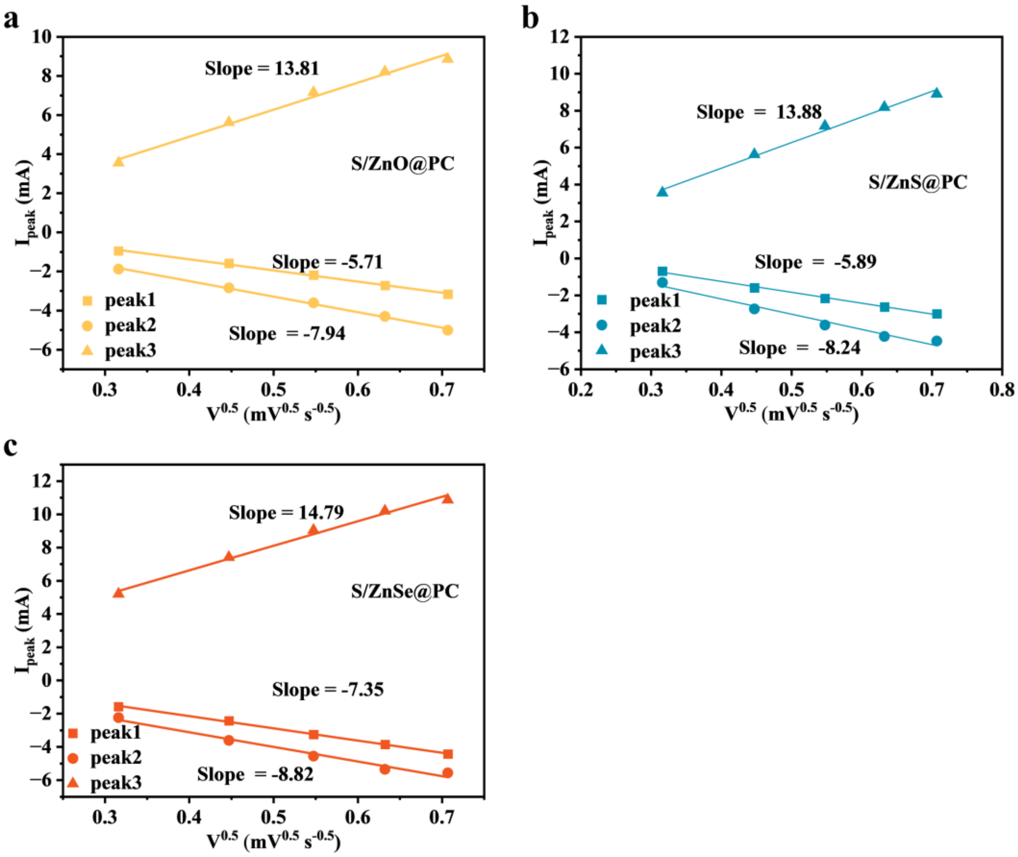

Figure S20. Linear relationship between I_p and $\nu^{1/2}$ for (a) S/ZnO@PC, (b) S/ZnS@PC and (c) S/ZnSe@PC.

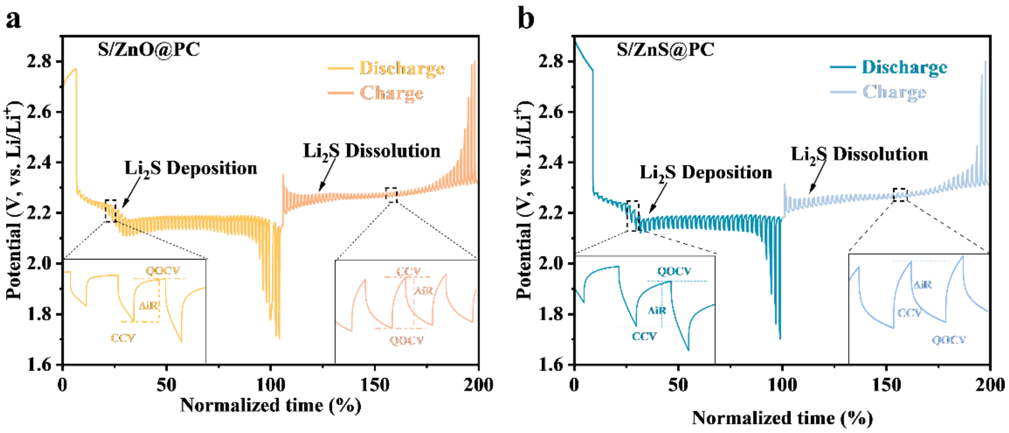

Figure S21. GITT curves of (a) S/ZnO@PC and (b) S/ZnS@PC.

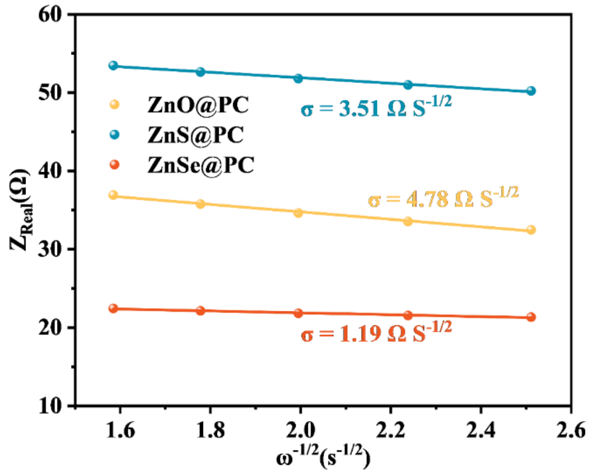

Figure S22. Relationships between Z' and $\omega^{-1/2}$ of S/ZnO@PC, S/ZnS@PC and S/ZnSe@PC.

Note : The Li^+ diffusion coefficient exhibits a linear relationship with the reciprocal of the Warburg coefficient, which can be calculated using the following equation:

$$D_{Li^+} = (R^2T^2)/(2A^2n^4F^4c^2\sigma^2)$$

Here, A, n, F, c, R, and T are constants representing the cathode surface area, the number of electrons transferred, Faraday's constant, Li^+ concentration, the gas constant, and the absolute temperature, respectively. D_{Li^+} and σ denote the Li^+ diffusion coefficient and the Warburg coefficient, respectively. The stress (σ) can be calculated using the following formula:

$$Z' = R_s + R_f + R_{ct} + \sigma\omega^{-0.5}$$

In this context, Z′ represents the real component of the total impedance, R_s denotes the bulk resistance, and ω is the angular frequency in the low-frequency region. As shown

in **Figure S22**, the value of σ can be determined, and D_{Li^+} is inversely proportional to σ.

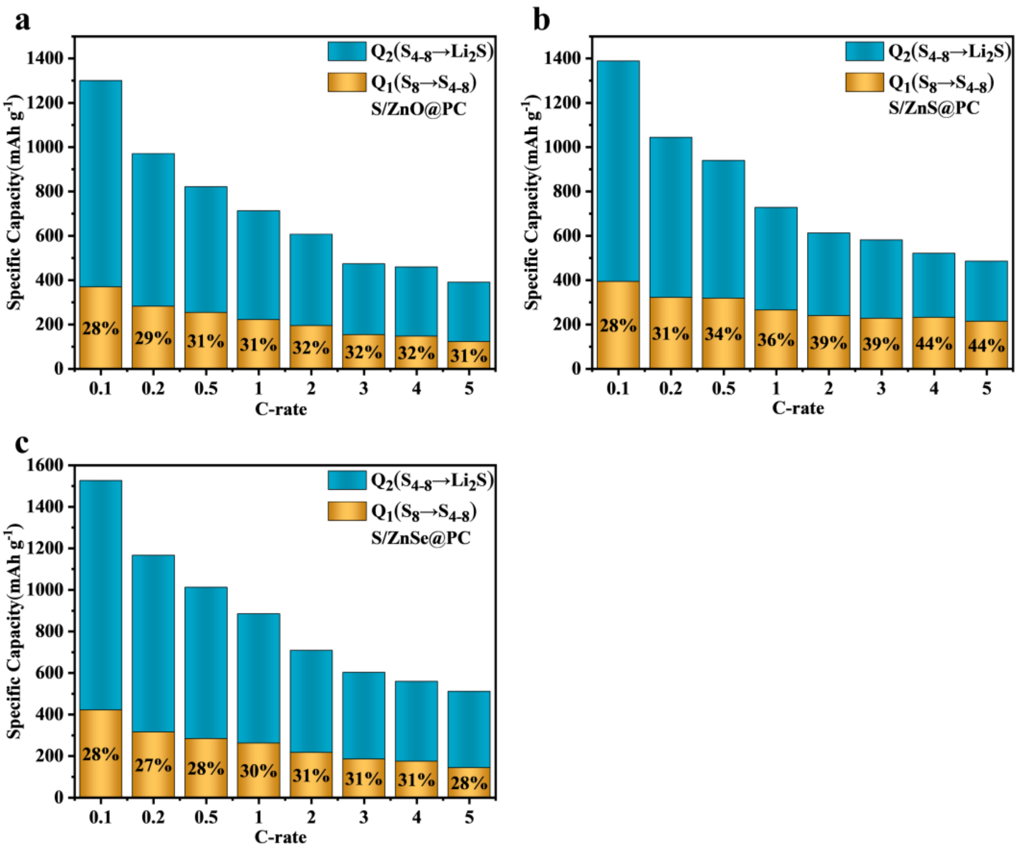

Figure S23. Q_2/Q_1 values for different samples of (a) S/ZnO@PC, (b) S/ZnS@PC and (c) S/ZnSe@PC.

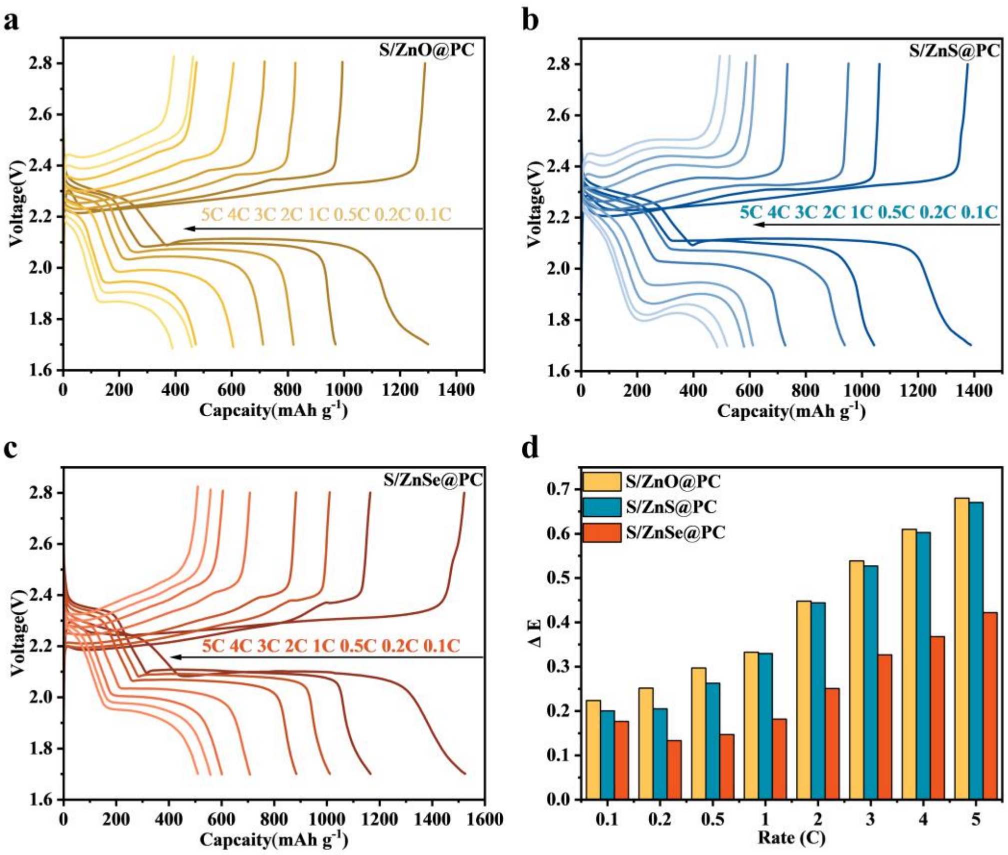

Figure S24. Discharge-charge curves of (a) S/ZnO@PC, (b) S/ZnS@PC and (c) S/ZnSe@PC at different multiplication rates. (d) The histogram of polarization potential of the batteries assembled with S/ZnO@PC, S/ZnS@PC and S/ZnSe@PC at different multiplication rates.

Analysis of charge-discharge profiles and corresponding polarization voltages at varying rates reveals significant differences in the electrochemical performance of the three cathode materials, which strongly correlates with their polysulfide anchoring and conversion capabilities. As shown in **Figure S24a**, the S/ZnO@PC electrode exhibits steep charge-discharge plateaus and rapid capacity degradation with increasing rates, accompanied by the highest polarization voltage. This indicates sluggish reaction kinetics and severe ohmic, concentration and electrochemical polarization. This primarily results from ZnO's limited ability to adsorb and convert polysulfides, failing to effectively suppress the shuttle effect, which leads to active material loss and reduced reaction reversibility. The S/ZnS@PC electrode (**Figure S24b**) shows improved

performance compared to S/ZnO@PC, with moderate polarization voltage, suggesting that ZnS provides some chemical adsorption of polysulfides and partially enhances reaction kinetics. However, significant capacity decay at high rates indicates that its catalytic conversion capability remains insufficient for high-rate cycling demands. Notably, the S/ZnSe@PC electrode (**Figure S24c**) demonstrates superior electrochemical performance, with the longest and flattest charge-discharge plateaus, high capacity retention across rates and significantly lower polarization voltage than the other two materials. The flat plateaus indicate more complete and stable sulfur species conversion ($Li_2S_8/Li_2S_6 \rightarrow Li_2S_4 \rightarrow Li_2S_2/Li_2S$), while the minimal polarization voltage confirms excellent reaction kinetics and rapid charge transfer. This is attributed to stronger chemical interactions between ZnSe@PC and polysulfides, which not only anchor LiPSs robustly but also efficiently catalyze the liquid-to-solid conversion process, thereby greatly suppressing shuttle effects, reducing polarization and enabling high specific capacity and outstanding rate capability. In summary, the electrochemical results are fully consistent with prior analysis: the S/ZnSe@PC cathode exhibits the best electrochemical performance due to its unique structure and superior adsorption-catalysis synergy toward polysulfides.

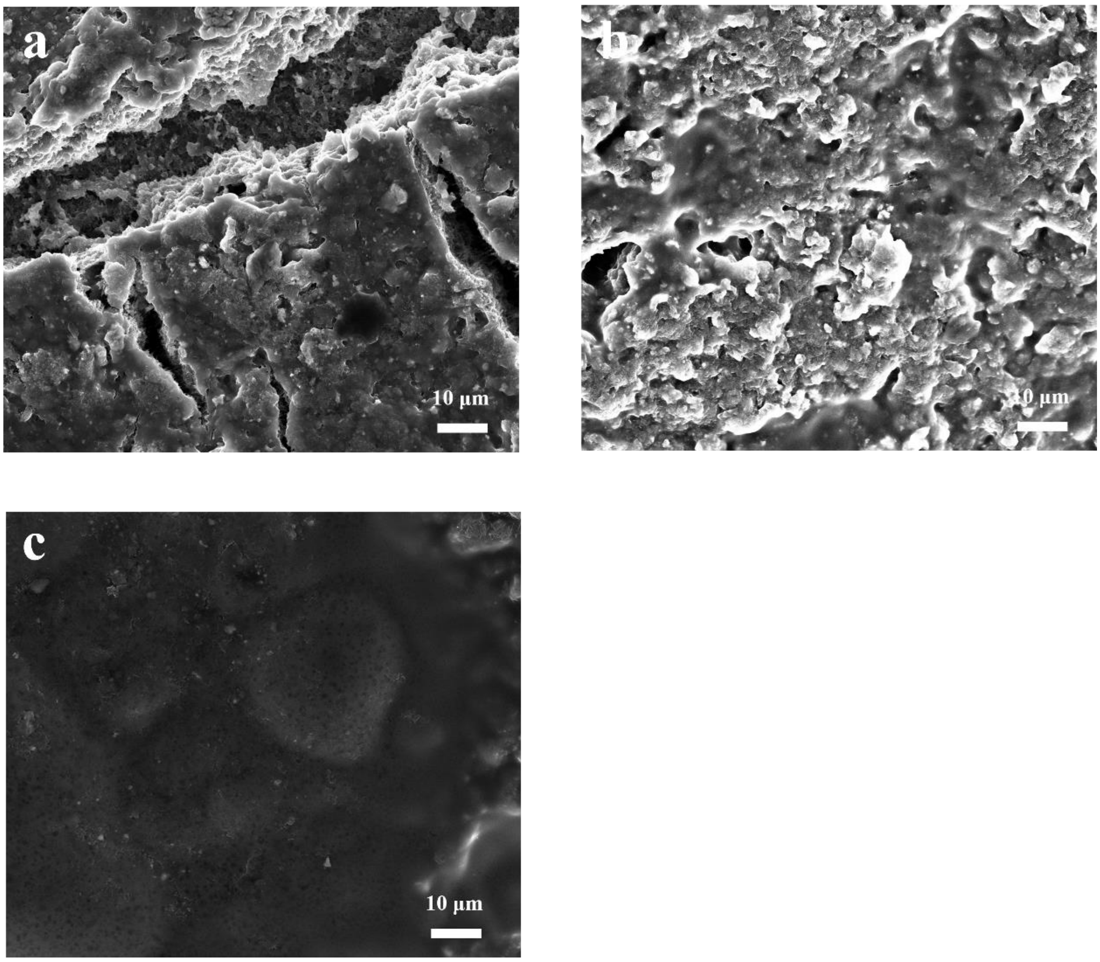

Figure S25. (a-c) Post-cycling SEM images of coated on the anode electrode utilizing carbon-coated aluminum foil: (a) S/ZnO@PC, (b) S/ZnS@PC and (c) S/ZnSe@PC.

Post-cycling SEM characterization of cathode electrodes reveals significant differences in structural stability influenced by the modification materials. As shown in the **Figure S25** electrodes with S/ZnO@PC (a) and S/ZnS@PC (b) exhibit rough and porous surface morphologies with visible cracks, indicating structural degradation caused by active material volume changes during cycling. This deterioration accelerates polysulfide shuttle effects and active material loss. In contrast, the S/ZnSe@PC electrode (c) demonstrates an exceptionally smooth and dense surface morphology with preserved structural integrity. This confirms that ZnSe modification effectively suppresses polysulfide shuttle, buffers volume expansion and provides superior

electrode stability, which correlates well with its outstanding electrochemical performance.

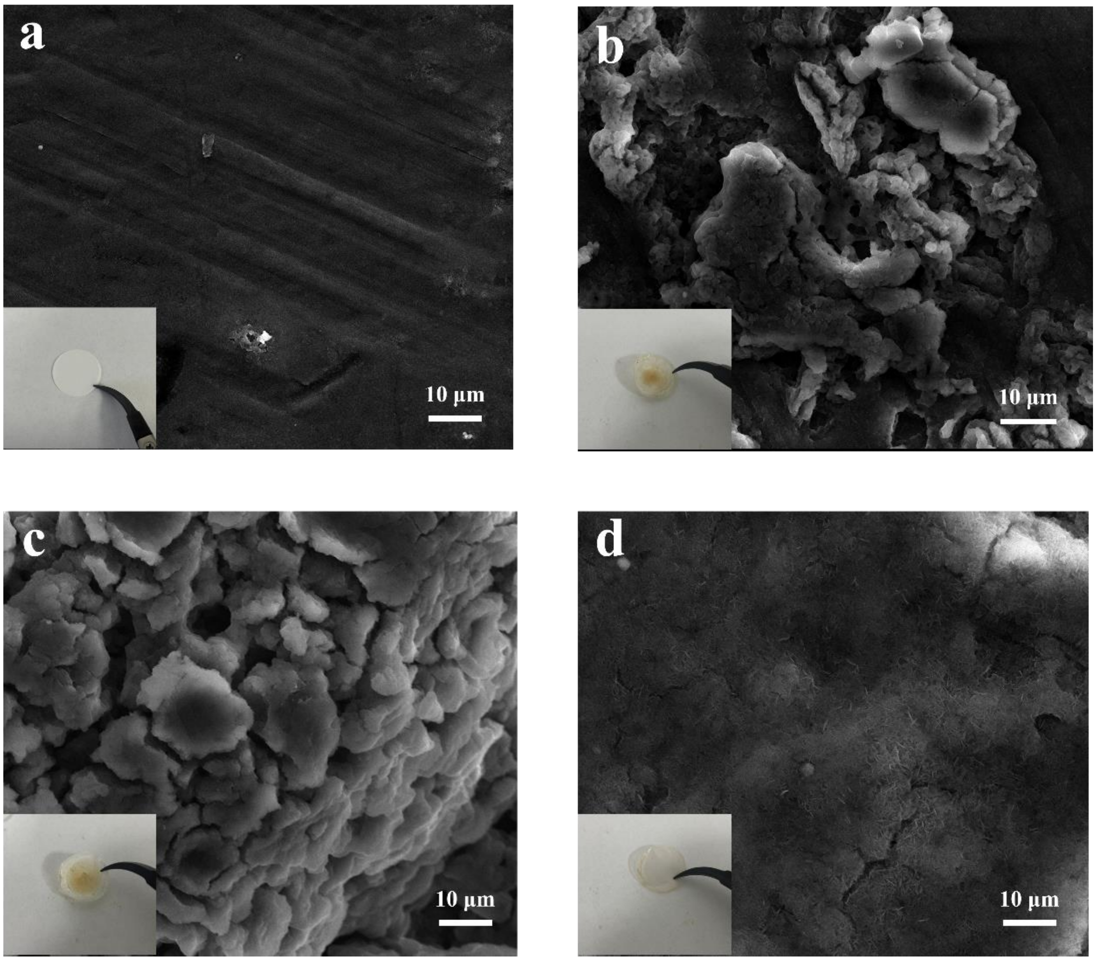

Figure S26. (a) SEM image and corresponding optical photograph of both the uncycled lithium electrode and separator. Post-cycling SEM images of Li anodes: (b) S/ZnO@PC, (c) S/ZnS@PC and (d) S/ZnSe@PC and the photographs of corresponding separators (inset).

SEM analysis of cycled lithium metal anodes, combined with optical images of corresponding separators, enables direct visual assessment of the polysulfide shuttle suppression capability afforded by different cathode materials. Compared to a pristine, uncycled electrode (**Figure S26a**), the lithium anode from the S/ZnO@PC cell (**Figure S26b**) exhibits substantial irregular, protruding deposits and a rough interface layer. This morphology indicates severe polysulfide shuttling, uncontrolled reductive deposition on the anode side, active material loss and interfacial degradation. The lithium anode paired with the S/ZnS@PC cathode (**Figure S26c**) shows finer, more

uniformly distributed deposits and an improved morphology compared to S/ZnO@PC. This suggests ZnS@PC possesses superior polysulfide anchoring capability over ZnO@PC, albeit insufficient to fully suppress shuttling. It can be reasonably inferred that the lithium anode from the S/ZnSe@PC cell (**Figure S26d**) would demonstrate the smoothest, cleanest surface with minimal deposits. This provides compelling evidence that the ZnSe-modified cathode most effectively suppresses polysulfide shuttling via an adsorption-catalysis mechanism, thereby protecting the lithium anode, maintaining interfacial stability and correlating with the superior cycling longevity of this cell group.

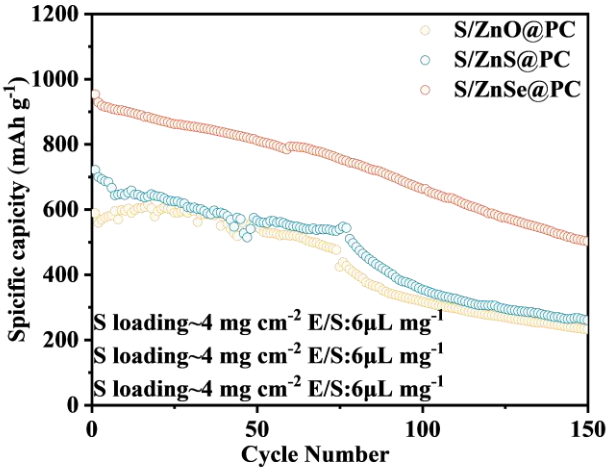

Figure S27. Comparison of high sulfur loading cycle performance of different electrodes at 0.2 C.

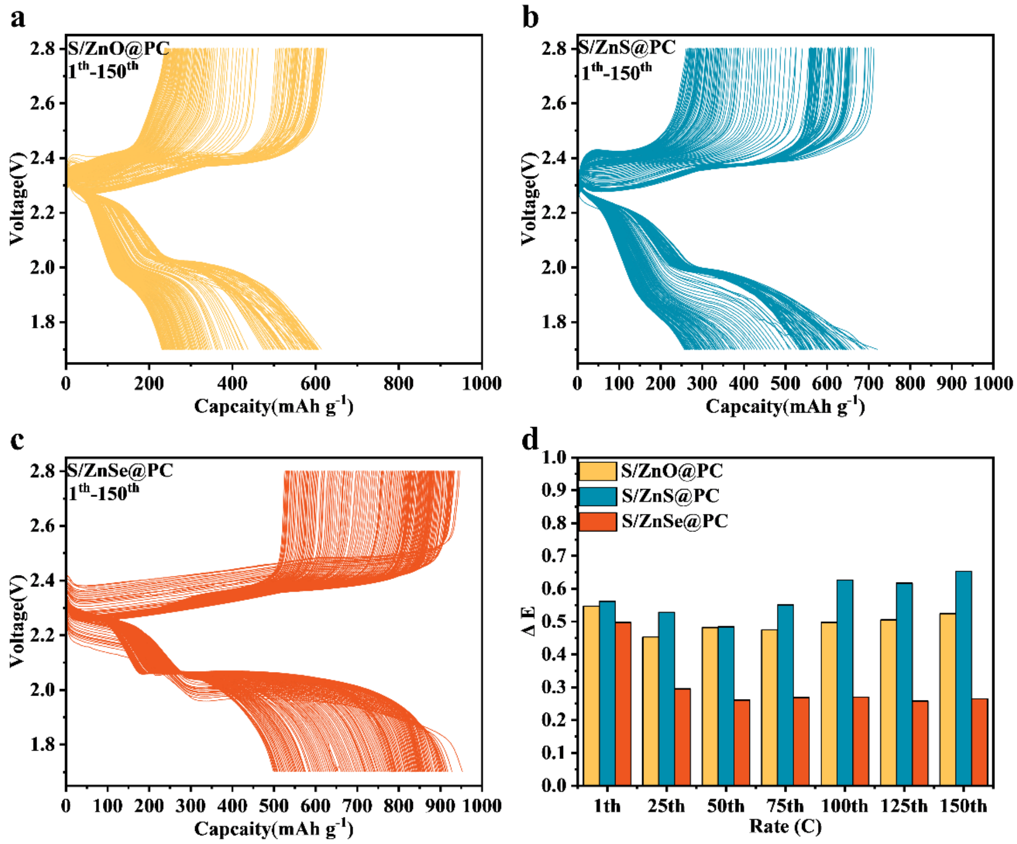

Figure S28. Discharge-charge curves demonstrating the cycling performance of (a) S/ZnO@PC, (b) S/ZnS@PC and (c) S/ZnSe@PC composites under high areal sulfur loading. (d) A comparison of the charge/discharge overpotential for batteries assembled with S/ZnO@PC, S/ZnS@PC and S/ZnSe@PC under high areal sulfur loading.

Detailed analysis of charge/discharge profiles and polarization voltage under high-loading conditions demonstrates that the S/ZnSe@PC electrode exhibits comprehensively superior electrochemical performance. It maintains the flattest and longest charge/discharge plateaus with minimal voltage decay throughout cycles 1-150, indicating the highest active material utilization and excellent reaction reversibility. This is primarily attributed to ZnSe's strong chemisorption and efficient catalytic conversion of lithium polysulfides (LiPSs), which effectively suppresses polysulfide dissolution and shuttling, ensuring stable conversion of active material during cycling. In contrast, S/ZnO@PC and S/ZnS@PC electrodes exhibit significant capacity fade and

increased plateau slope, indicating aggravated polarization, reduced active material utilization and rapid capacity degradation during cycling. This phenomenon is closely associated with polysulfide shuttling, insufficient conductivity and volume expansion effects. More importantly, the polarization voltage (ΔV) of S/ZnSe@PC is significantly lower than comparative samples under all tested conditions. The low polarization voltage indicates that ZnSe@PC effectively reduces ohmic, electrochemical and concentration polarization within the cell, markedly improving reaction kinetics. This benefit stems from the synergistic effect between ZnSe@PC and the porous carbon (PC) carrier: ZnSe provides strong adsorption and catalytic sites, while the porous carbon network ensures excellent electron conduction and ion diffusion, collectively facilitating rapid liquid-solid conversion (Li_2S_4 → Li_2S). These results fully demonstrate the significant application advantages of ZnSe@PC modified materials in high-loading lithium-sulfur battery cathodes, providing an effective material design strategy for achieving high energy density and long cycle life.

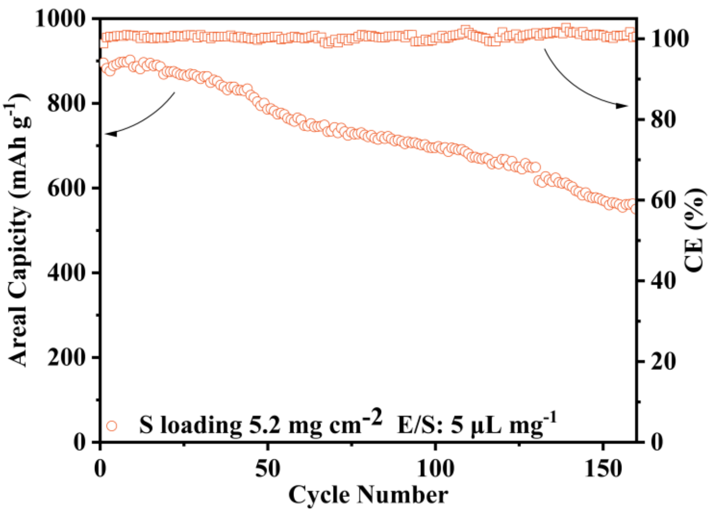

Figure S29. High sulfur loading cycle performance of ZnSe@PC/S electrode at 0.1C.

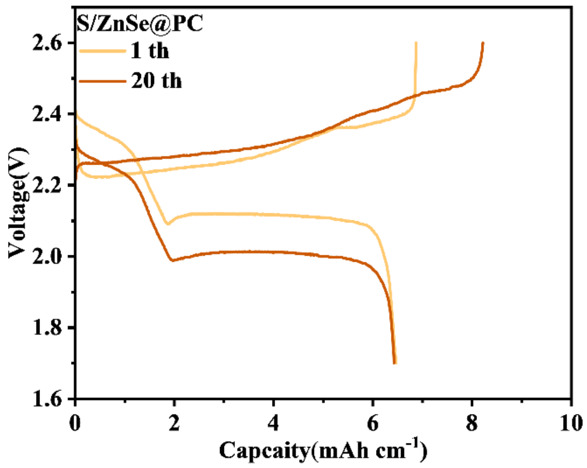

Figure S30. Constant-current charge/discharge curves for the 1st and 20th cycles.

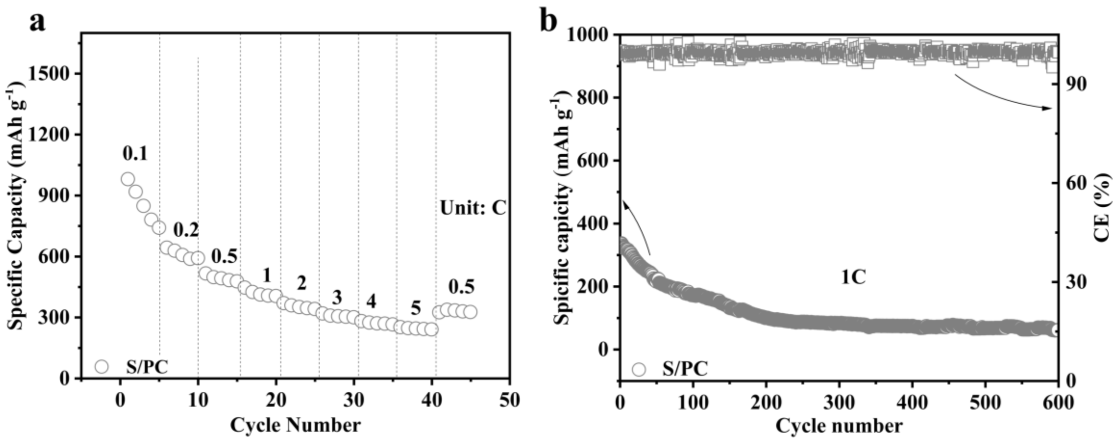

Figure S31. (a) Rate performance of S/PC, (b) Cycling performance at 1C of S/PC.

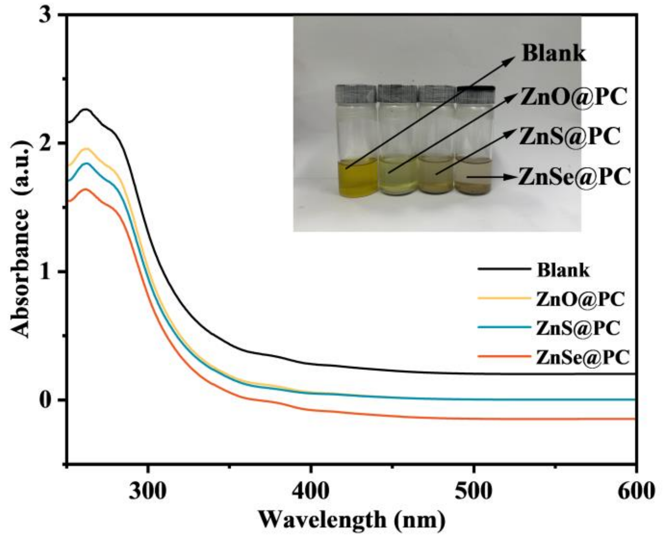

Figure S32. UV−vis spectra and photograph of Li_2S_6 solution with ZnX@PC (X = O, S, Se).

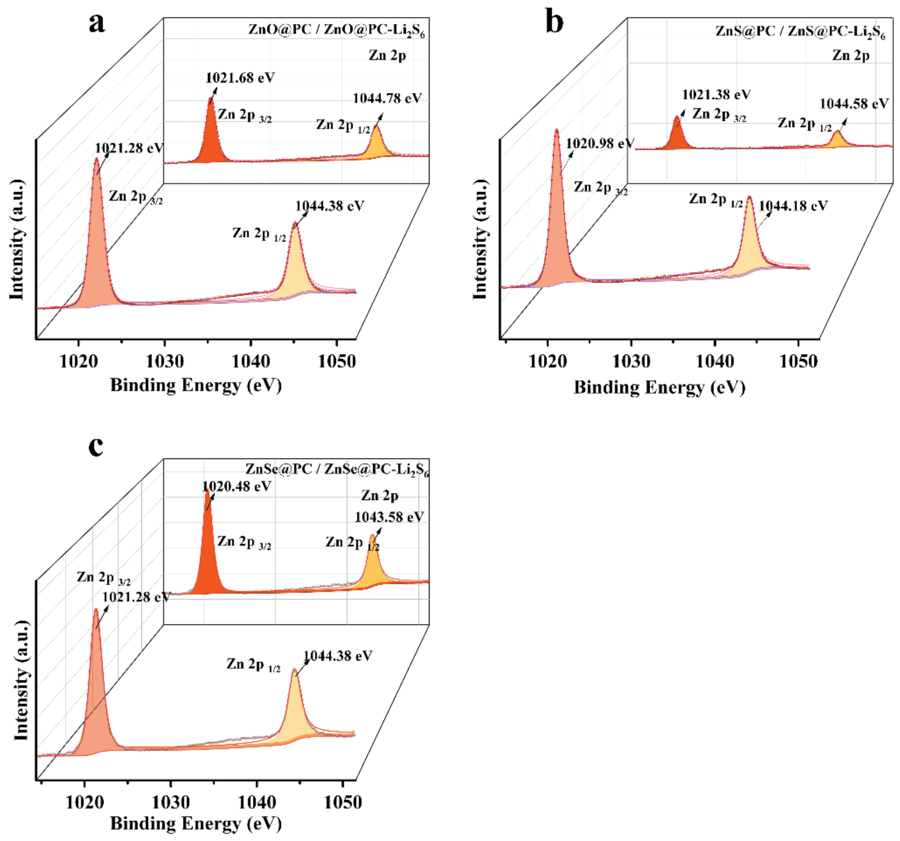

Figure S33. Zn 2p XPS spectras of (a) ZnO@PC, (b) ZnS@PC and (c) ZnSe@PC before and after adsorbing Li_2S_6.

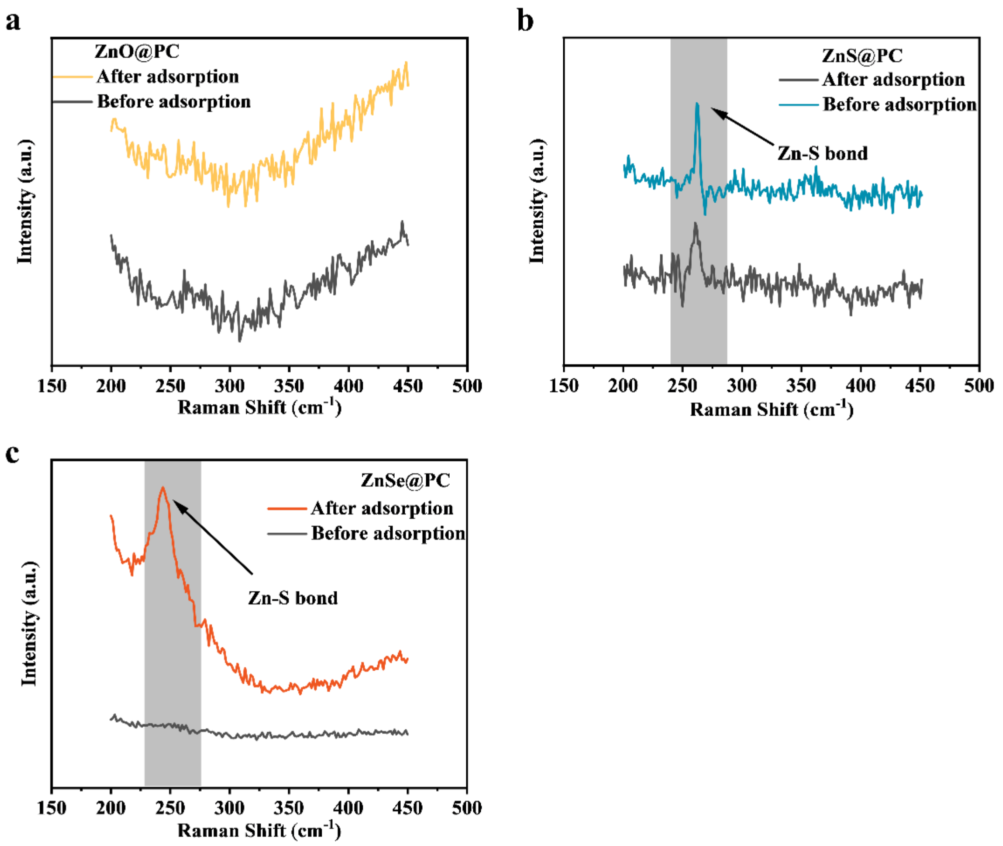

Figure S34. The Raman spectras of (a) ZnO@PC, (b) ZnS@PC and (c) ZnSe@PC powders before and after adsorbing Li_2S_6 solution.

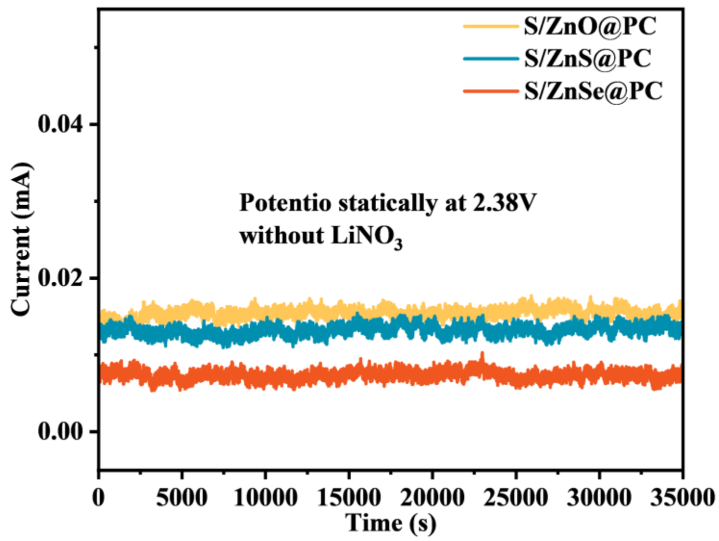

Figure S35. Shuttle current test of S/ZnO@PC, S/ZnS@PC and S/ZnSe@PC.

The cells were initially activated through three charge/discharge cycles at 0.1C, followed by a constant-current discharge to 2.38 V and subsequent transition to potentiostatic mode. The shuttle currents were recorded during potentiostatic hold proceee.

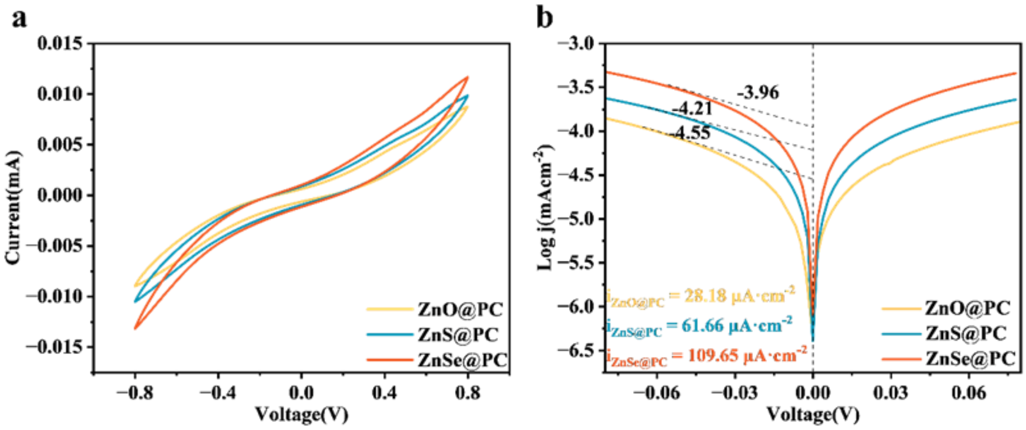

Figure S36. (a) CV curves of the symmetric cells with various electrodes at a scan rate of 5 mV s^{-1}. (b) Tafel plot of the symmetric cells with various electrodes.

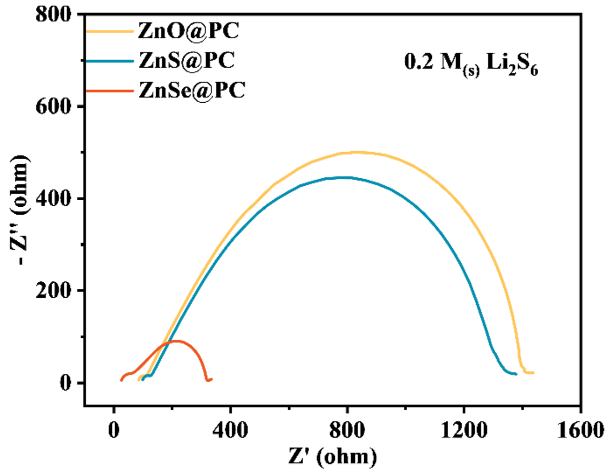

Figure S37. EIS results of Li_2S_6 symmetric cells.

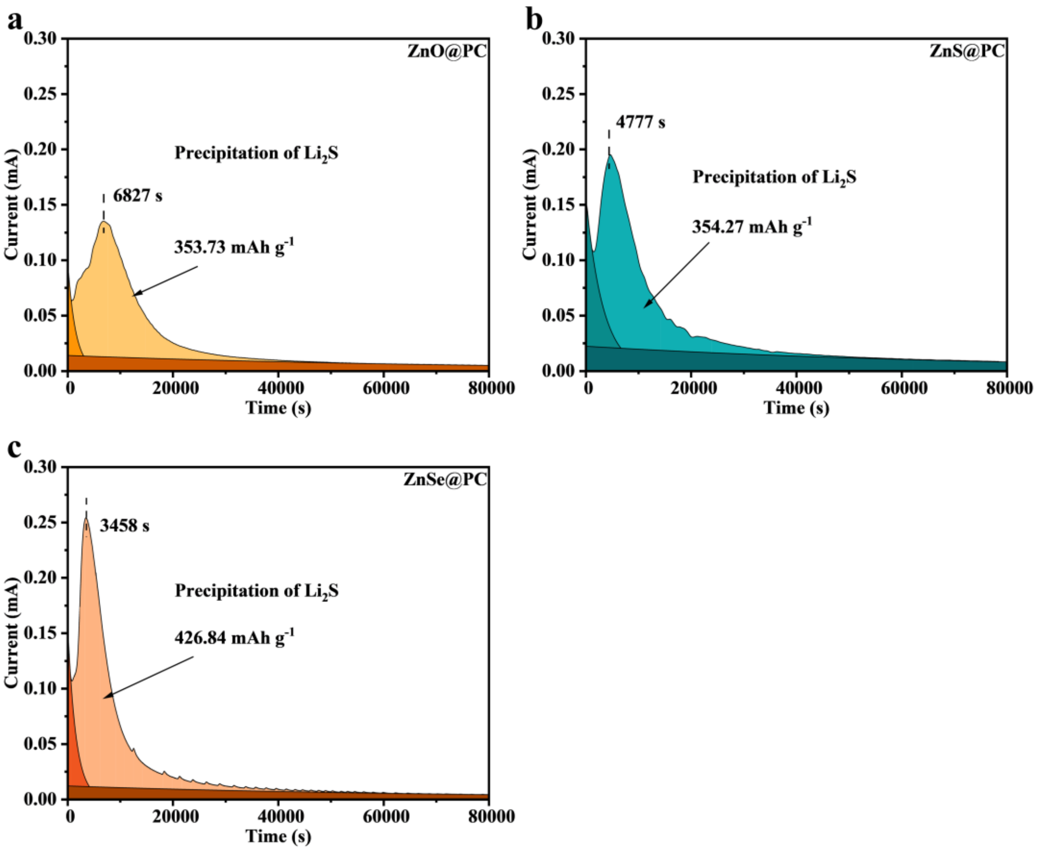

Figure S38. Li_2S deposition curves on (a) ZnO@PC, (b) ZnS@PC and (c) ZnSe@PC.

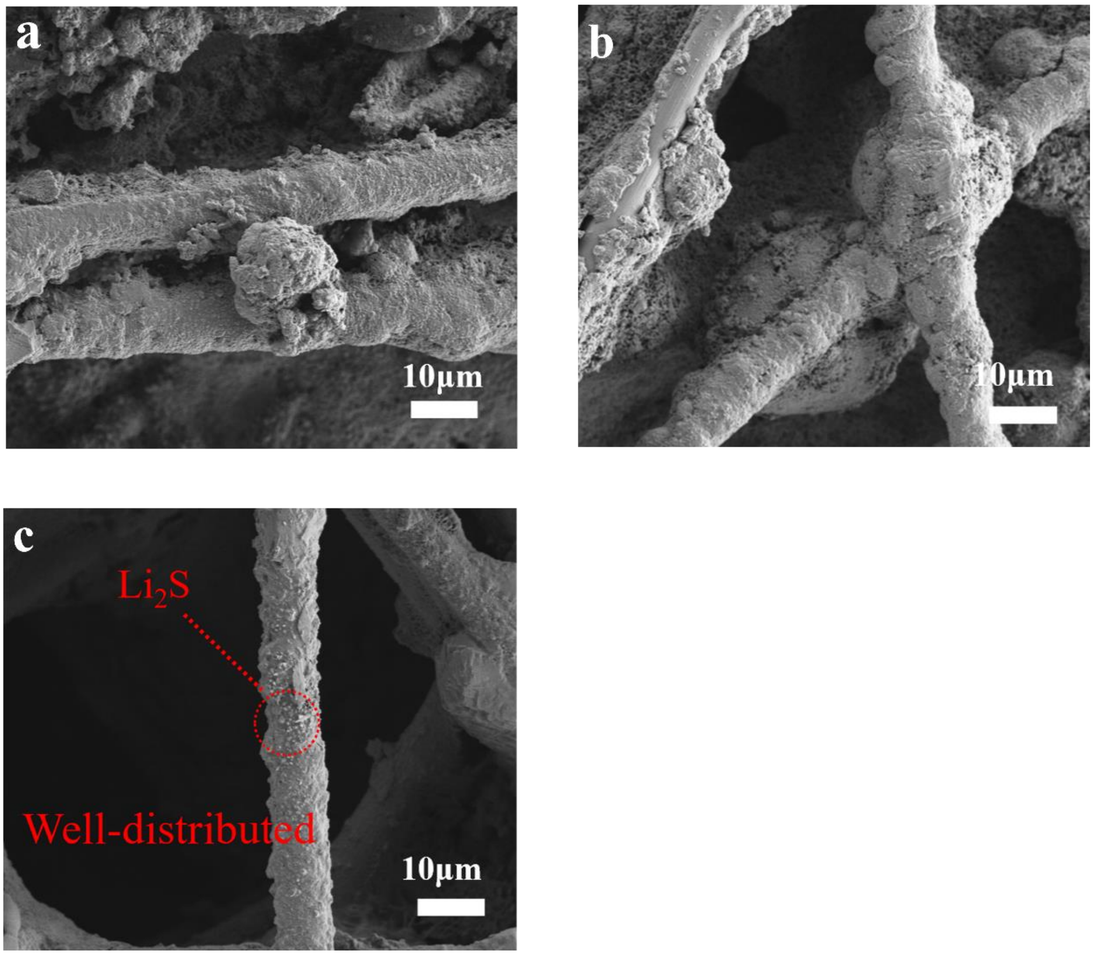

Figure S39. SEM images of the Li_2S nucleation and growth morphology on (a) ZnO@PC, (b) ZnS@PC and (c) ZnSe@PC.

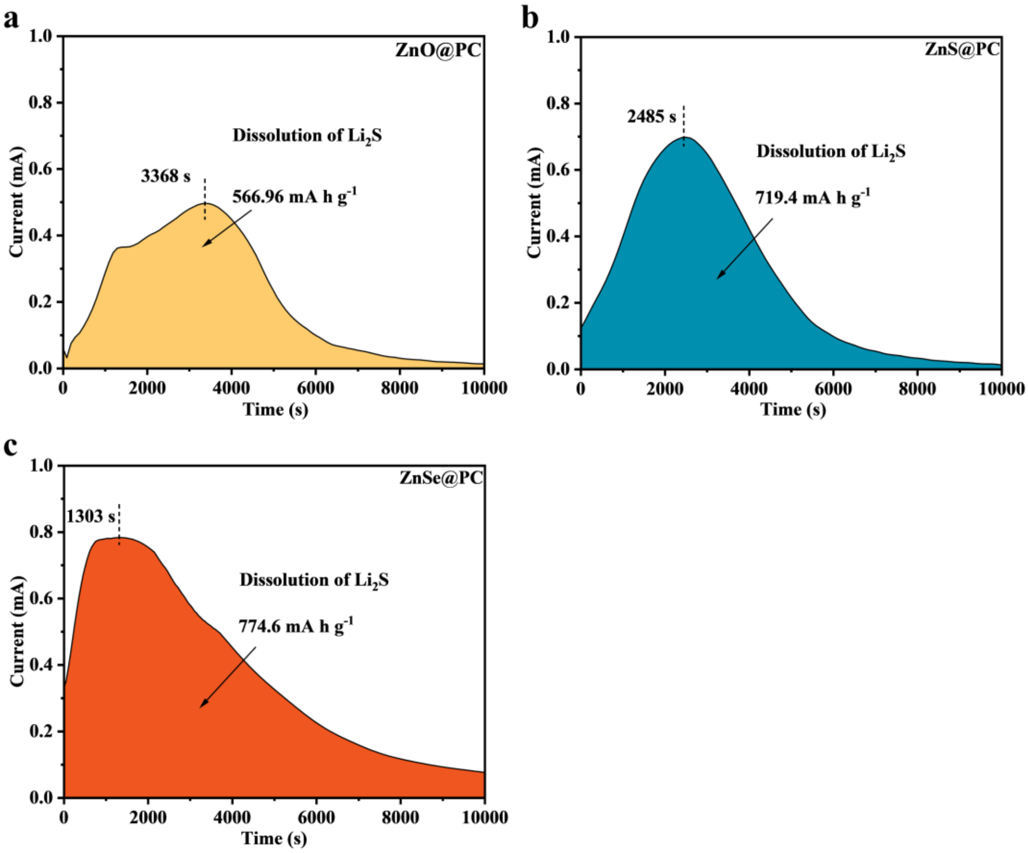

Figure S40. Potentiostatic charge profiles of Li_2S dissolution on (a) ZnO@PC, (b) ZnS@PC and (c) ZnSe@PC.

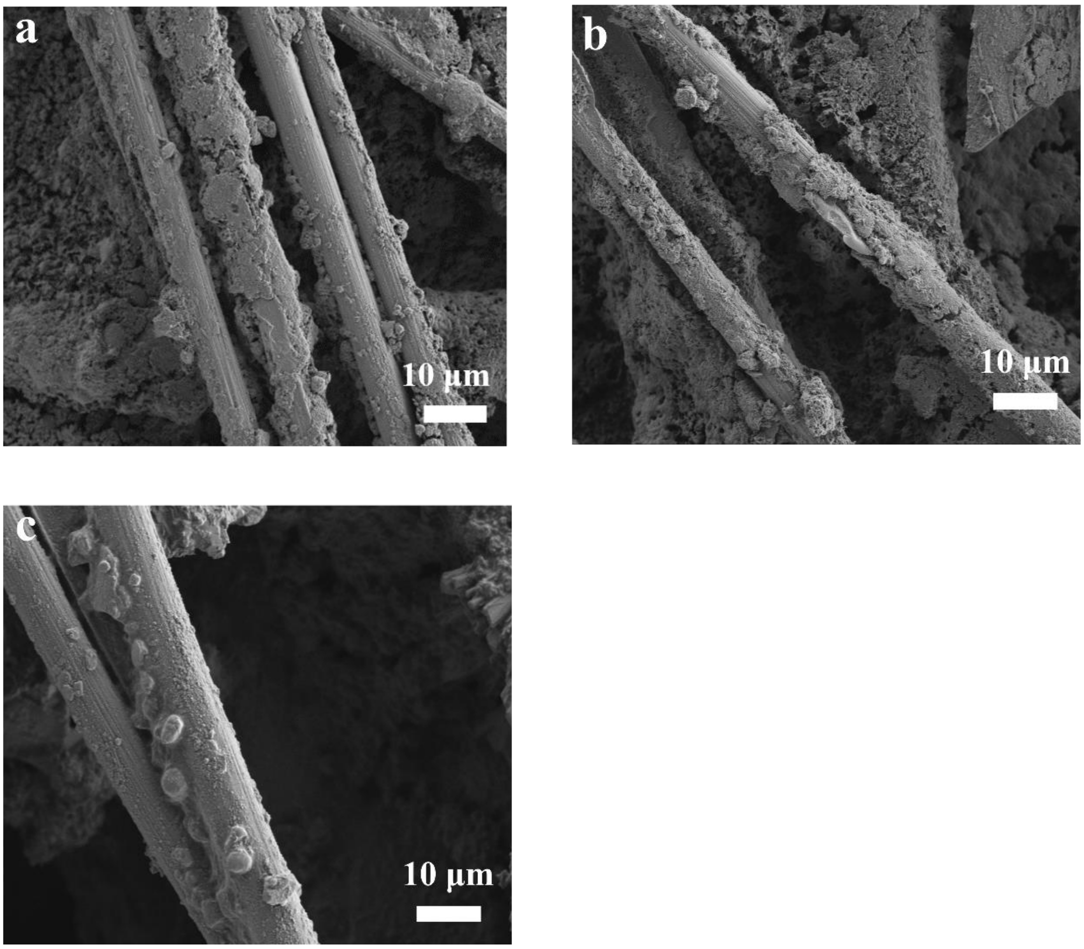

Figure S41. SEM images of the Li_2S dissolution and growth morphology on (a) ZnO@PC, (b) ZnS@PC and (c) ZnSe@PC.

In the Li_2S dissolution experiment, ZnSe@PC demonstrated the highest dissolution capacity and the fastest response time compared to ZnO@PC and ZnS@PC (**Figure S38**). SEM characterization further confirmed complete dissolution of Li_2S on the ZnSe@PC electrode, whereas ZnO@PC and ZnS@PC electrodes exhibited varying degrees of Li_2S aggregation. This indicates superior redox kinetics in the ZnSe@PC electrode.

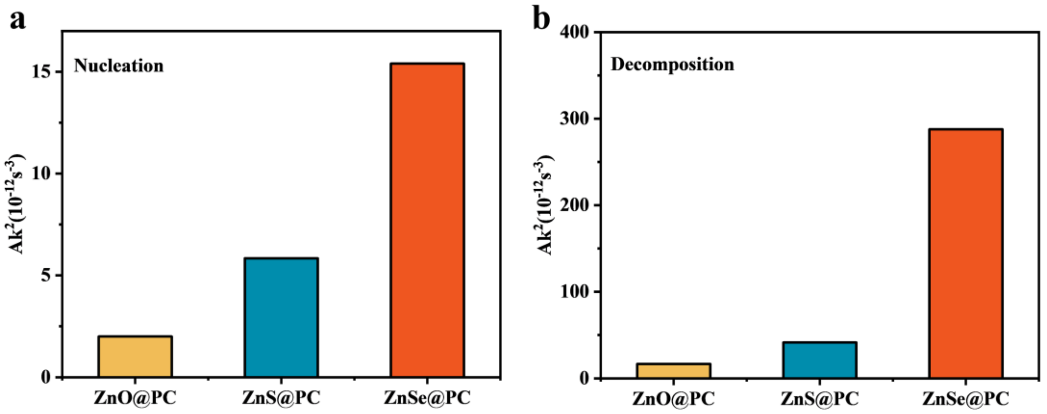

Figure S42. The calculated growth rate of (a) Li_2S nucleation and (b) Li_2S decomposition processes.

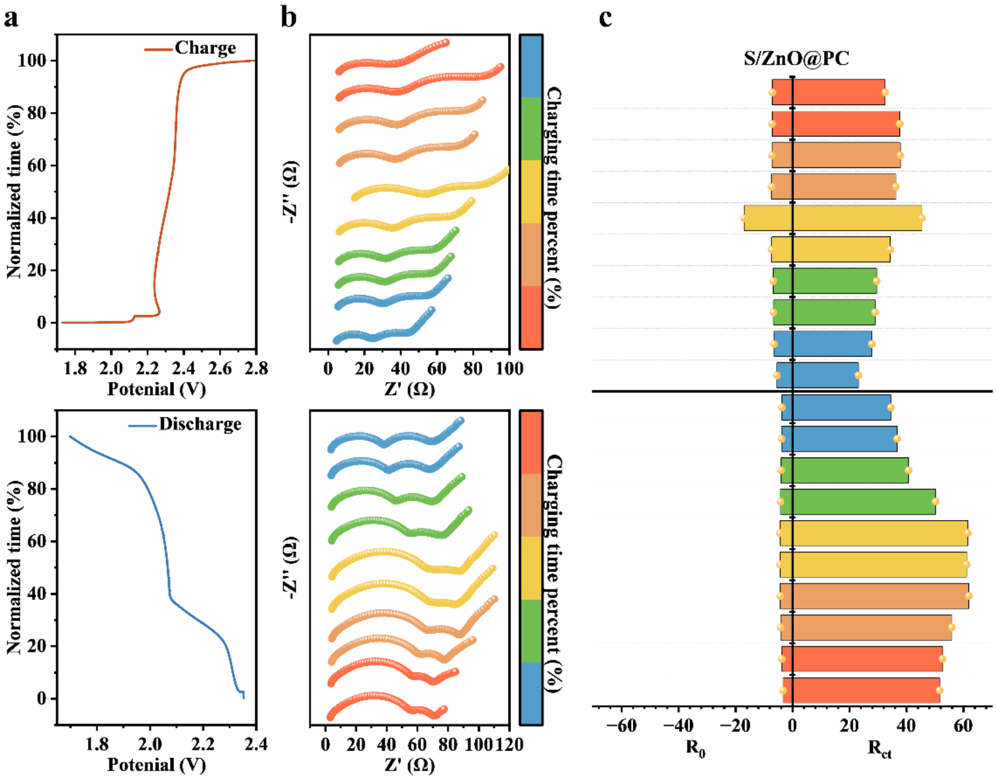

Figure S43. (a) Corresponding charge/discharge curves during in-situ electrochemical impedance spectroscopy. (b) ZnO@PC-based cell from the in-situ EIS measurements. (c) Variation in R_0 and R_{ct} of cells during discharging and charging process.

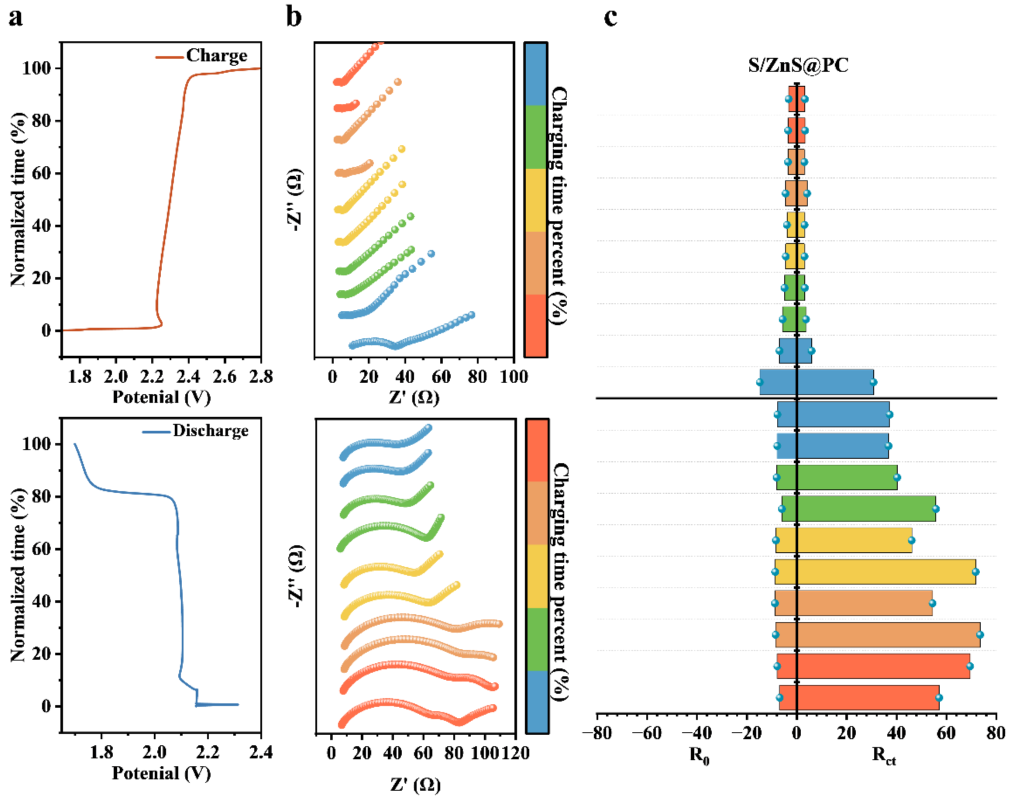

Figure S44. (a) Corresponding charge/discharge curves during in-situ electrochemical impedance spectroscopy. (b) ZnS@PC-based cell from the in-situ EIS measurements. (c) Variation in R_0 and R_{ct} of cells during discharging and charging process.

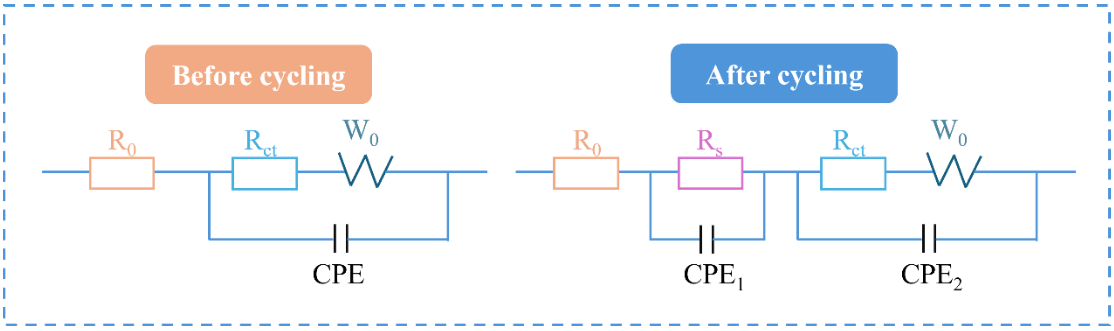

Figure S45. The fitted EIS equivalent circuits of Li-S cells (a) before cycling and (b) after cycles.

Note: R_0: The ohmic resistance R_0 is obtained by the high-frequency intercept on the real axis in the Nyquist plot, which can be used to reflect the equivalent series resistance (ESR) of the system; R_{int}: The electrolyte/electrode interfacial resistance; R_{ct}: The charge-transfer resistance; W_0: Warburg diffusion impedance.

Comparative analysis of the equivalent circuits reveals that the interfacial process in the lithium-sulfur battery evolves from a simple charge transfer mechanism to a more complex one characterized by two-time constants, corresponding to surface film impedance and charge transfer resistance, after cycling. The newly introduced R_s-CPE_1 parallel element directly reflects the formation of a solid electrolyte interphase (SEI) or an insoluble Li_2S deposition layer on the electrode surface after cycling. The formation of this interfacial layer serves as a key factor contributing to increased battery polarization and the evolution of reaction kinetics.

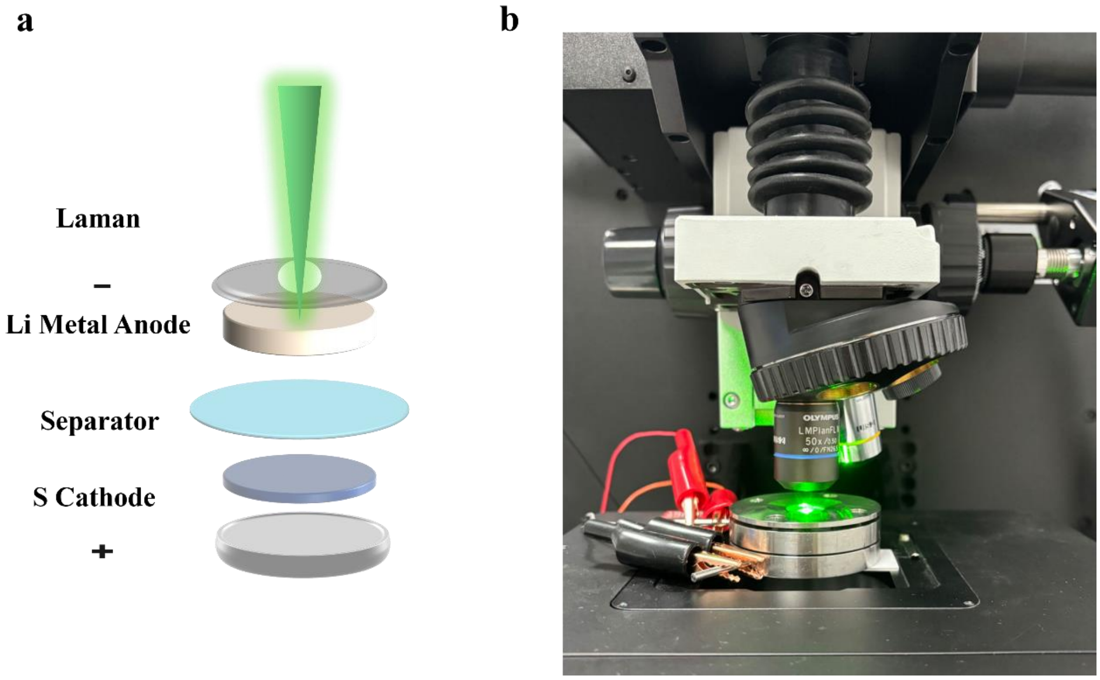

Figure S46. (a) Schematic illustration of in-situ Raman detection and (b) Actual photograph of in-situ Raman testing process.

In-situ Raman spectroscopy during battery cycling was conducted using a stainless steel mold cell. A perforation was designed on the lithium anode side, allowing the Raman laser to pass through the quartz glass window on the mold cell. This configuration bypassed interference from the lithium electrode and enabled direct, real-time monitoring of polysulfide species that had traversed the separator due to the shuttle effect, capturing their characteristic spectral signals. This approach directly revealed the underlying causes of sulfur species loss and related issues during the electrochemical reactions. Galvanostatic charge/discharge tests were performed at a rate of 0.2C.

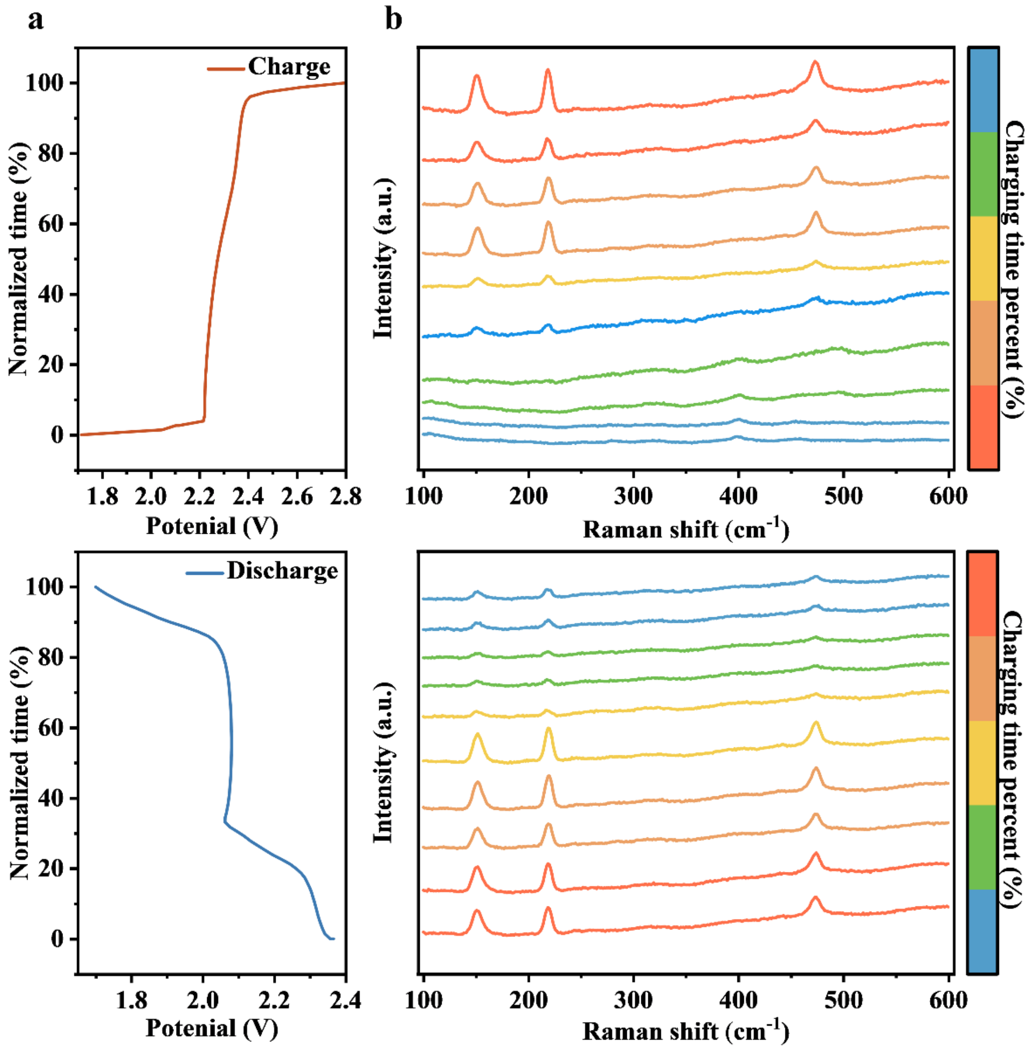

Figure S47. (a) Corresponding charge/discharge profiles during in-situ Raman spectroscopy testing. (b) Selected Raman spectroscopy of Li-S battery based on S/ZnO@PC.

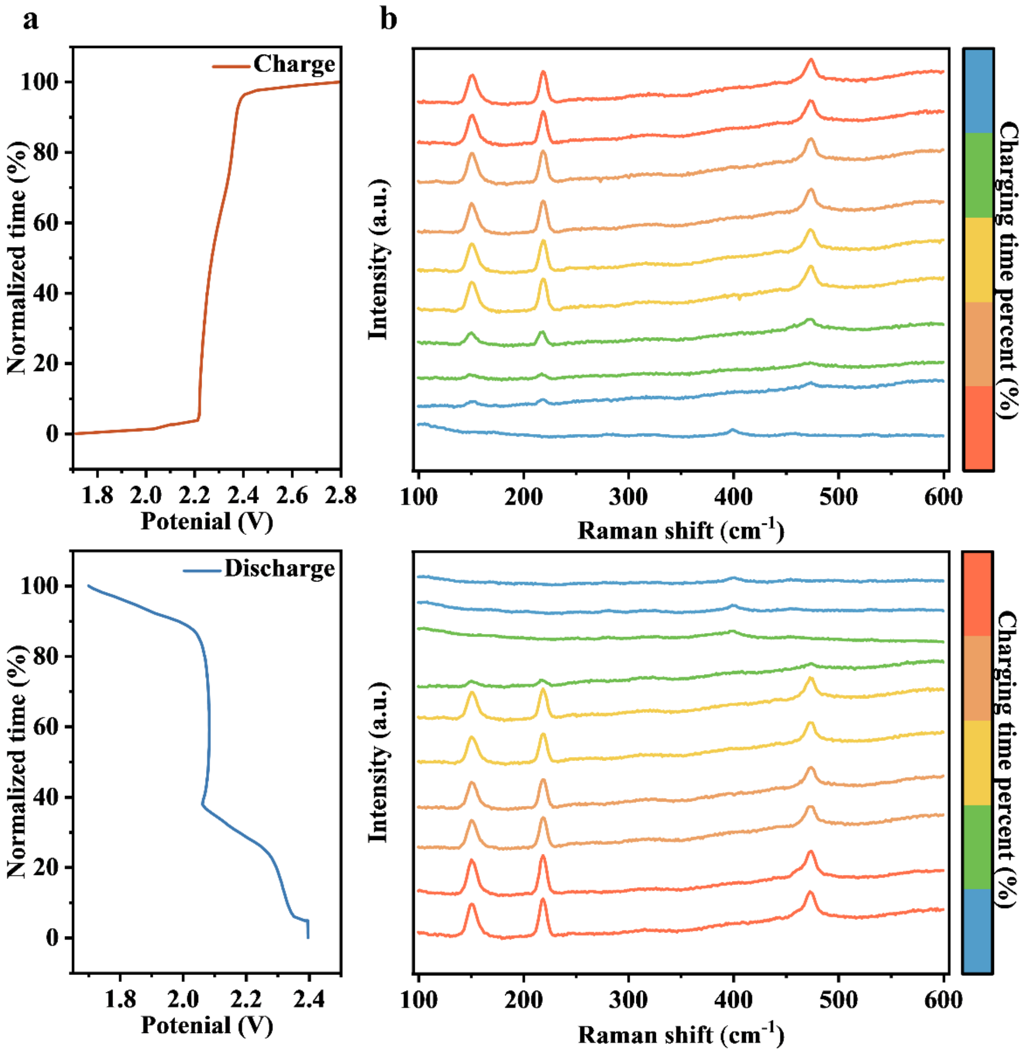

Figure S48. (a) Corresponding charge/discharge profiles during in-situ Raman spectroscopy testing. (b) Selected Raman spectroscopy of Li-S battery based on S/ZnS@PC.

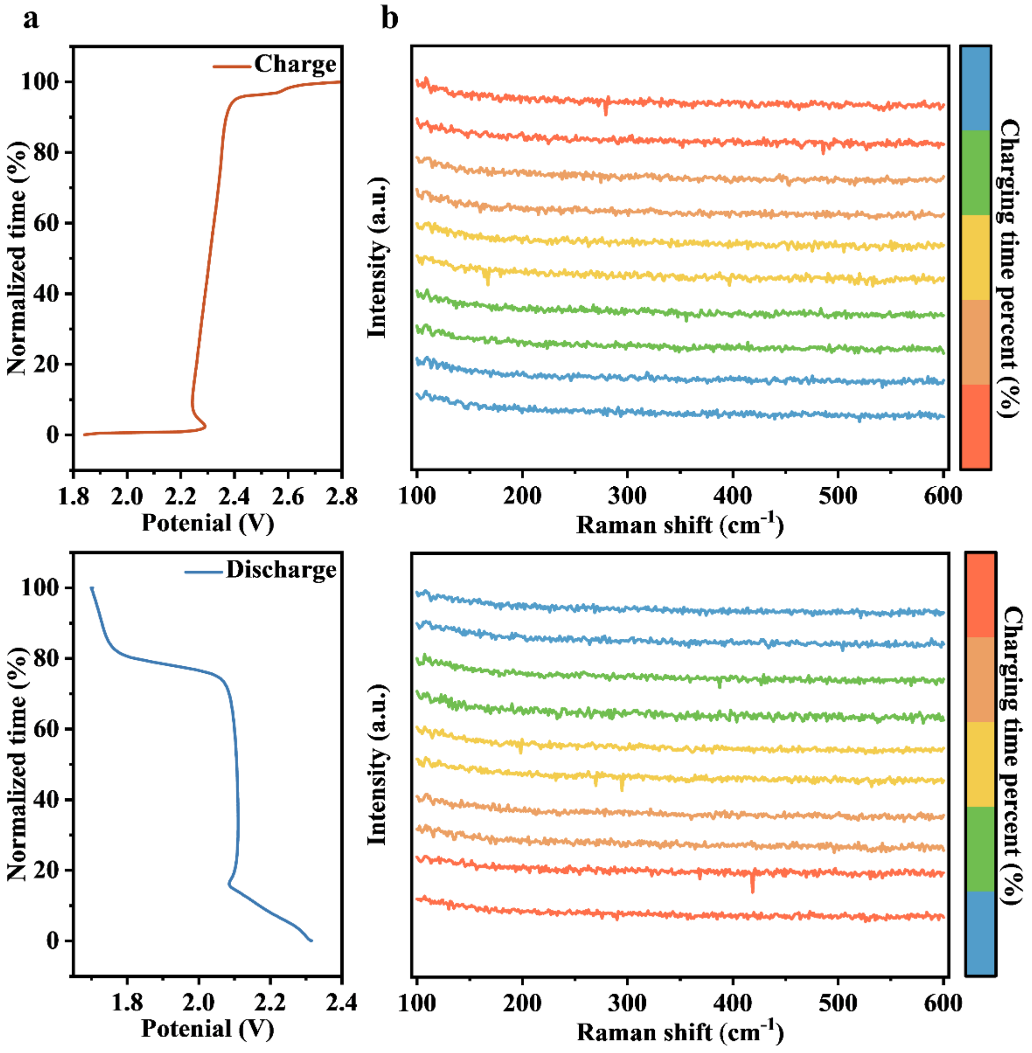

Figure S49. (a) Corresponding charge/discharge profiles during in-situ Raman spectroscopy testing. (b) Selected Raman spectroscopy of Li-S battery based on S/ZnSe@PC.

4. Supplementary tables

Table S1. The specific surface areas of ZnO@PC, ZnS@PC and ZnSe@PC

	**ZnO@PC**	**ZnS@PC**	**ZnSe@PC**
**Specific surface areas**	137.23 m^2 g^{-1}	219.78 m^2 g^{-1}	407.54 m^2 g^{-1}

Table S2. Detailed parameters and calculated Li diffusion coefficient (D_{Li^+}) values at different redox peak positions for different electrodes.

Electrode	Peak	n	A / cm^{-2}	D_{Li^+} / mol cm^{-3}	| Slope, $I_p/v^{0.5}$ |	D_{Li^+} / cm^{2}s^{-1}
S/ZnO@PC	1	2	1.13	0.0029	5.71	5.35×10^{-6}
	2				7.94	1.03×10^{-5}
	3				13.81	3.13×10^{-5}
S/ZnS@PC	1				5.28	4.57×10^{-6}
	2				6.70	7.37×10^{-6}
	3				15.21	3.46×10^{-5}
S/ZnSe@PC	1				7.35	8.87×10^{-6}
	2				8.82	1.27×10^{-5}
	3				14.79	3.59×10^{-5}

Table S3. Comparison of electrochemical performance of the Li-S battery in this study with those in the previously reported Li-S batteries.

Host	Capacity (Low rate) [mAh g^{-1}]	Capacity (High rate) [mAh g^{-1}]	Cycle number	Decay Rate Per cycle	Capacity retention	Sulfer loading (mg cm^{-2})	Areal Sulfur Capacity (mAh cm^{-2})	Reference
ZnSe@PC	1526 (0.1 C)	559.1 (5 C)	600 (1 C)	0.043%	58%	5.18	4.1	This work
0.25%-Cu-2-T	1013.9 (0.2 C)	660.7 (2 C)	100 (0.2 C)	0.23%	77%	6.5	5.1	13
MCG	1103.0 (0.2 C)	763.0 (2 C)	710 (1 C)	0.056%	60.2%	3.5	4.0	14
PCMs	941.0 (0.2 C)	480.0 (4 C)	900 (2 C)	0.060%	46%	4.3	4.0	15
Co@NHCMs	1089.0 (0.2 C)	692.0 (4 C)	500 (1 C)	0.08%	60%	5.1	50	16
Ni-NC(p)	924.7 (0.2 C)	706.2 (2 C)	600 (0.5 C)	0.078%	46.8%	N/A	N/A	17
ZnSe-$CoSe_2$	1127.8 (0.2 C)	403.6 (5 C)	400 (1 C)	0.126%	50.6%	6.1	4.2	18
HMCS/S@GO	976.0 (0.2 C)	626.0 (2 C)	300 (1 C)	0.203%	61%	N/A	N/A	19
HCMS	1162.0 (0.2 C)	360.0 (5 C)	900 (1 C)	0.040%	57.3%	4.8	4.0	20
Ni-NC(p)	924.7 (0.2 C)	706.2 (2 C)	600 (0.5 C)	0.078%	46.8%	N/A	N/A	17
Co/Co_3O_4 NHC	957.1 (0.2 C)	447.9 (5 C)	500 (1 C)	0.032%	84.1%	4.0	1.5	22
CoS_2@NGCNs	1003.8 (0.2 C)	525.3 (2 C)	300 (1 C)	0.075%	77%	N/A	N/A	23
Fe_xN@C	1148.7 (0.2 C)	858.1 (2 C)	500 (1 C)	0.095%	76.7%	N/A	N/A	24
CNT/$NiSe_2$	1175.1 (0.2 C)	870.6 (1 C)	900.2 (1 C)	0.103%	69.0%	8.2	7.5	25
Cu-2	1121.1 (0.2 C)	808.6 (2 C)	868.2 (2 C)	0.076%	69.5%	7.7	7.8	26
Al_2O_3@mG	1307.5 (0.2 C)	515.6 (3 C)	766.8 (1 C)	0.032%	48.8%	5.1	4.7	27

Table S4. Comparison of pouch cell cycling performance between this work and other reported studies.

Host	Sulfer loading (mg cm^{-2})	E/S (μL mg^{-1})	Capacity (mAh g^{-1})	Cycle number	Capacity retention	Reference
ZnSe@PC	7	4	953.3	20	96.2%	This work
GSCC	5	7	1022.6	32	83.8%	[28]
BTT	N/A	2.6	1021.7	18	77.9%	[29]
P-$RuSe_2$/NC	3.89	11.4	998.1	60	84.3%	[30]
CoTPyP-Mn	N/A	8	700	30	91.0%	[31]
Ni-NG	4	5	892.8	40	72.0%	[32]

Table S5. The nucleation and dissolution capacity of ZnO@PC, ZnS@PC and ZnSe@PC

	**ZnO@PC**	**ZnS@PC**	**ZnSe@PC**
Nucleation capacity	353.73 mAh g^{-1}	254.27 mAh g^{-1}	426.84 mAh g^{-1}
Dissolution capacity	566.96 mAh g^{-1}	719.40 mAh g^{-1}	774.60 mAh g^{-1}

Table S6. Fitted result of R_0 of Li-S battery with various cathodes during in-situ EIS measurement.

Stage	ZnO@PC	ZnS@PC	ZnSe@PC
DOD~10%	3.23	6.72	1.99
DOD~20%	3.68	7.77	2.14
DOD~30%	4.10	8.42	2.31
DOD~40%	4.25	8.65	2.46
DOD~50%	4.32	8.54	2.4
DOD~60%	4.37	8.28	1.92
DOD~70%	4.15	5.87	2.29
DOD~80%	3.94	7.99	2.22
DOD~90%	3.79	7.77	2.16
DOD~100%	3.67	7.71	2.10
SOC~10%	4.62	11.04	4.31
SOC~20%	5.44	5.14	3.71
SOC~30%	5.66	4.20	3.10
SOC~40%	5.78	3.63	2.77
SOC~50%	6.23	3.22	2.69
SOC~60%	14.55	2.89	2.64
SOC~70%	6.27	3.28	2.58
SOC~80%	6.11	2.56	2.54
SOC~90%	6.09	2.53	2.50
SOC~100%	6.03	2.37	2.51

Table S7. Fitted result of R_{ct} of Li−S battery with various cathodes during in-situ EIS measurement.

**Stage**	**ZnO@PC**	**ZnS@PC**	**ZnSe@PC**
DOD~10%	51.74	57.22	51.13
DOD~20%	52.71	69.61	50.26
DOD~30%	56.04	73.65	49.38
DOD~40%	62.01	54.58	37.70
DOD~50%	61.22	71.86	51.85
DOD~60%	61.65	46.21	41.75
DOD~70%	50.34	55.92	30.08
DOD~80%	40.86	40.38	25.46
DOD~90%	36.85	36.91	22.84
DOD~100%	34.59	37.39	20.65
SOC~10%	19.88	23.16	5.96
SOC~20%	23.99	4.53	3.67
SOC~30%	24.92	2.86	3.04
SOC~40%	25.41	2.47	3.15
SOC~50%	29.53	2.37	3.33
SOC~60%	39.02	2.38	3.60
SOC~70%	31.16	3.18	3.70
SOC~80%	32.52	2.36	3.67
SOC~90%	32.34	2.55	3.26
SOC~100%	27.97	2.51	3.17

References

1. G. Kresse and J. Hafner, Ab initio molecular dynamics for liquid metals, *Physical Review B*, 1993, **47**, 558-561.
2. G. Kresse and J. Hafner, Ab initio molecular-dynamics simulation of the liquid-metal--amorphous-semiconductor transition in germanium, *Physical Review B*, 1994, **49**, 14251-14269.
3. J. P. Perdew, K. Burke and M. Ernzerhof, Generalized Gradient Approximation Made Simple, *Physical Review Letters*, 1996, **77**, 3865-3868.
4. G. Kresse and D. Joubert, From ultrasoft pseudopotentials to the projector augmented-wave method, *Physical Review B*, 1999, **59**, 1758-1775.
5. S. Grimme, S. Ehrlich and L. Goerigk, Effect of the damping function in dispersion corrected density functional theory, *Journal of Computational Chemistry*, 2011, **32**, 1456-1465.
6. M. Jia, Y. Jin, C. Zhao, P. Zhao and M. Jia, High electrochemical sodium storage performance of ZnSe/CoSe@N-doped porous carbon synthesized by the in-situselenization of ZIF-8/67 polyhedron, *Applied Surface Science*, 2020, **518**, 146259.
7. W. Zhang, D. Hong, Z. Su, S. Yi, L. Tian, B. Niu, Y. Zhang and D. Long, Tailored ZnO-ZnS heterostructure enables a rational balancing of strong adsorption and high catalytic activity of polysulfides for Li-S batteries, *Energy Storage Materials*, 2022, **53**, 404-414.
8. C. Wei, B. Xi, P. Wang, Y. Liang, Z. Wang, K. Tian, J. Feng and S. Xiong, In Situ Anchoring Ultrafine ZnS Nanodots on 2D MXene Nanosheets for Accelerating Polysulfide Redox and Regulating Li Plating, *Advanced Materials*, 2023, **35**, 2303780.
9. X. Zhao, Y. Guan, X. Du, G. Liu, J. Li and G. Li, Ordered macroporous V-doped ZnO framework impregnated with microporous carbon nanocages as multifunctional sulfur reservoir in lithium-sulfur batteries, *Chemical Engineering Journal*, 2022, **431**, 134242.
10. Z. Shen, X. Jin, J. Tian, M. Li, Y. Yuan, S. Zhang, S. Fang, X. Fan, W. Xu, H. Lu, J. Lu and H. Zhang, Cation-doped ZnS catalysts for polysulfide conversion in lithium–sulfur batteries, *Nature Catalysis*, 2022, **5**, 555-563.
11. Y. He, L. Wang, C. Dong, C. Li, X. Ding, Y. Qian and L. Xu, In-situ rooting ZnSe/N-doped hollow carbon architectures as high-rate and long-life anode materials for half/full sodium-ion and potassium-ion batteries, *Energy Storage Materials*, 2019, **23**, 35-45.
12. Y. Li, F. Wu and S. Xiong, Embedding ZnSe nanoparticles in a porous nitrogen-doped carbon framework for efficient sodium storage, *Electrochimica Acta*, 2019, **296**, 582-589.
13. Q. Yang, S. Shen, Z. Han, G. Li, D. Liu, Q. Zhang, L. Song, D. Wang, G. Zhou and Y. Song, An Electrolyte Engineered Homonuclear Copper Complex as Homogeneous Catalyst for Lithium–Sulfur Batteries, *Advanced Materials*, 2024, **36**, 2405790.
14. P. Wang, B. Xi, Z. Zhang, N. Song, W. Chen, J. Feng and S. Xiong, Dual-Functional MgO Nanocrystals Satisfying Both Polysulfides and Li Regulation toward Advanced Lithium−Sulfur Full Batteries, *Small*, 2021, **17**, 2103744.
15. S. Liu, T. Zhao, X. Tan, L. Guo, J. Wu, X. Kang, H. Wang, L. Sun and W. Chu, 3D

pomegranate-like structures of porous carbon microspheres self-assembled by hollow thin-walled highly-graphitized nanoballs as sulfur immobilizers for Li–S batteries, *Nano Energy*, 2019, **63**, 103894.

16. L. Su, J. Zhang, Y. Chen, W. Yang, J. Wang, Z. Ma, G. Shao and G. Wang, Cobalt-embedded hierarchically-porous hollow carbon microspheres as multifunctional confined reactors for high-loading Li-S batteries, *Nano Energy*, 2021, **85**, 105981.
17. Y. Li, Y. Zeng, Y. Chen, D. Luan, S. Gao and X. W. Lou, Mesoporous N-rich Carbon with Single-Ni Atoms as a Multifunctional Sulfur Host for Li-S Batteries, *Angewandte Chemie International Edition*, 2022, **61**, e202212680.
18. L. Feng, P. Yu, X. Fu, Z.-M. Zhang, K. Davey, Y. Wang, Z. Guo and W. Yang, Regulating Polysulfide Diffusion and Deposition via Rational Design of Core–Shell Active Materials in Li–S Batteries, *ACS Nano*, 2022, **16**, 7982-7992.
19. R. Zhe, T. Zhu, X. Wei, Y. Ren, C. Qing, N. Li and H.-E. Wang, Graphene oxide wrapped hollow mesoporous carbon spheres as a dynamically bipolar host for lithium–sulfur batteries, *Journal of Materials Chemistry A*, 2022, **10**, 24422-24433.
20. X. Zhou, J. Tian, Q. Wu, J. Hu and C. Li, N/O dual-doped hollow carbon microspheres constructed by holey nanosheet shells as large-grain cathode host for high loading Li-S batteries, *Energy Storage Materials*, 2020, **24**, 644-654.
21. C. Zhao, G.-L. Xu, Z. Yu, L. Zhang, I. Hwang, Y.-X. Mo, Y. Ren, L. Cheng, C.-J. Sun, Y. Ren, X. Zuo, J.-T. Li, S.-G. Sun, K. Amine and T. Zhao, A high-energy and long-cycling lithium–sulfur pouch cell via a macroporous catalytic cathode with double-end binding sites, *Nature Nanotechnology*, 2021, **16**, 166-173.
22. Y. Jeon, J. Lee, H. Jo, H. Hong, L. Y. S. Lee and Y. Piao, Co/Co_3O_4-embedded N-doped hollow carbon composite derived from a bimetallic MOF/ZnO Core-shell template as a sulfur host for Li-S batteries, *Chemical Engineering Journal*, 2021, **407**, 126967.
23. S.-D. Seo, D. Park, S. Park and D.-W. Kim, “Brain-Coral-Like” Mesoporous Hollow CoS_2@N-Doped Graphitic Carbon Nanoshells as Efficient Sulfur Reservoirs for Lithium–Sulfur Batteries, *Advanced Functional Materials*, 2019, **29**, 1903712.
24. D. Xie, Y. Xu, Y. Wang, X. Pan, E. Härk, Z. Kochovski, A. Eljarrat, J. Müller, C. T. Koch, J. Yuan and Y. Lu, Poly(ionic liquid) Nanovesicle-Templated Carbon Nanocapsules Functionalized with Uniform Iron Nitride Nanoparticles as Catalytic Sulfur Host for Li–S Batteries, *ACS Nano*, 2022, **16**, 10554-10565.
25. Z. Han, R. Gao, T. Wang, S. Tao, Y. Jia, Z. Lao, M. Zhang, J. Zhou, C. Li, Z. Piao, X. Zhang and G. Zhou, Machine-learning-assisted design of a binary descriptor to decipher electronic and structural effects on sulfur reduction kinetics, *Nature Catalysis*, 2023, **6**, 1073-1086.
26. Q. Yang, J. Cai, G. Li, R. Gao, Z. Han, J. Han, D. Liu, L. Song, Z. Shi, D. Wang, G. Wang, W. Zheng, G. Zhou and Y. Song, Chlorine bridge bond-enabled binuclear copper complex for electrocatalyzing lithium–sulfur reactions, *Nature Communications*, 2024, **15**, 3231.
27. J. Gu, Z. Shi, Y. Mu, Y. Wu, M. Tian, Z. Chen, K. Chen, H. Gu, M. Lu, L. Zeng, Y. Song, Q. Zhang and J. Sun, Sustaining vacancy catalysis via conformal graphene overlays boosts practical Li–S batteries, *Energy & Environmental Science*, 2025, **18**, 5940-5951.

28. W. Jiang, X. Jin, B. Li, Y. Qu, L. Wang, C. Song, M. Pei, T. Zhang, X. Jian and F. Hu, Wide temperature range adaptable electric field driven binder for advanced lithium-sulfur batteries, *Nature Communications*, 2025, **16**, 7860.
29. W. Guo, W. Zhang, Y. Si, D. Wang, Y. Fu and A. Manthiram, Artificial dual solid-electrolyte interfaces based on in situ organothiol transformation in lithium sulfur battery, *Nature Communications*, 2021, **12**, 3031.
30. B. Li, P. Wang, J. Yuan, N. Song, J. Feng, S. Xiong and B. Xi, P-doped $RuSe_2$ on Porous N-Doped Carbon Matrix as Catalysts for Accelerated Sulfur Redox Reactions, *Angewandte Chemie International Edition*, 2024, **63**, e202408906.
31. Y. Zhao, Z. Shang, M. Feng, H. Zhong, Y. Du, W. Chen, Y. Wang, J. Zou, Y. Chen, H. Wang, Y. Wang, J.-N. Zhang and G. Qu, Oriented Assembly of 2D Metal-Pyridylporphyrinic Framework to Regulate the Redox Kinetics in Li−S Batteries, *Advanced Materials*, 2025, **37**, 2501869.
32. X. Kong, Y. Li, G. Cai, W. Liu, J. Xu, C. Liu, G. Zhang, Y. Wang, Z. Lu, J. Zhang, X. Wu, D. Zhang, H. Luo, S. Jin and H. Ji, Asymmetric Coordinated Single-Atom Catalysts Offering Zero-Order Sulfur Redox Kinetics for High Performance Li–S Batteries, *Angewandte Chemie International Edition*, 2025, **n/a**, e202510212.
